# Lysosomal Targeted Cyclometallic Iridium(Ⅲ) Salicylaldehyde-Coumarin Schiff Base Complexes and Anticancer Application

**DOI:** 10.3389/fchem.2022.906954

**Published:** 2022-05-10

**Authors:** Ruixi Xu, Yuting Wu, Zhe Liu, Jinfeng Liu, Xicheng Liu

**Affiliations:** ^1^ Institute of Anticancer Agents Development and Theranostic Application, School of Chemistry and Chemical Engineering, Qufu Normal University, Qufu, China; ^2^ College of Life Sciences, Qufu Normal University, Qufu, China

**Keywords:** Iridium(Ⅲ) complexes, Schiff base, coumarin, anticancer, lysosomal targeted

## Abstract

Natural coumarin derivatives and cyclometallic iridium (Ⅲ) (Ir^Ⅲ^) complexes have attracted much attention in the field of anticancer. In this study, six coumarin-modified cyclometallic Ir^Ⅲ^ salicylaldehyde Schiff base complexes ([(ppy)_2_Ir(O^N)]/[(ppy-CHO)_2_Ir(O^N)]) were designed and synthesized. Compared with coumarin and Ir^Ⅲ^ complex monomers, target complexes exhibited favorable cytotoxic activity toward A549 and BEAS-2B cells. These complexes could induce extensive apoptosis of A549 cell (late apoptosis), which was represented by the disturbance of cell cycle (G_1_-phase) and the accumulation of intracellular reactive oxygen species, exhibiting an anticancer mechanism of oxidation. With the help of suitable fluorescence of these complexes, no conflict with the probes, confocal detection confirmed that complexes showed an energy-dependent cellular uptake mechanism and triggered lysosome-mediated apoptosis in A549 cell line. Above all, our findings reveal the design of a lysosomal targeting cyclometallic Ir^Ⅲ^ Schiff base complexes and provide a new idea for the design of integrated drugs for diagnosis and treatment.

## 1 Introduction

Recently, organometallic iridium (Ⅲ) complexes have been reported to show favorable anticancer potential because of their different anticancer mechanism from cisplatin (a widely used drug in clinical), including the effect on cell REDOX and protein with strong inhibitory activity, good biocatalytic performance. Platinum drugs can be activated in cells by hydrolysis, and then act on DNA in cells, affect DNA cross-linking, block DNA strand replication, and make cells die. Iridium complexes exert their anticancer effects through a variety of different mechanisms, such as catalyzing cell oxidation, inhibiting protein activity and so on (Barry and Sadler 2012; Cutillas et al., 2013; Liu and Sadler 2014; He et al., 2015; Li et al., 2017; Chen et al., 2020). Numerous studies have demonstrated that the Ir^Ⅲ^ complex can be used as a potential substitute for platinum-based drugs to overcome a series of shortcomings (drug resistance, nephrotoxicity etc.) (Li et al., 2020). Fluorescent cyclometallic Ir^Ⅲ^ complexes have been widely used in bioimaging and biosensing applications because of their excellent photophysical properties, including flexible absorption/emission wavelength, large Stokes shifts, and high quantum yields, among others. (Kang et al., 2014; Lo 2015; Lo and Tso 2015; Wang et al., 2016). Compared with half-sandwich iridium complexes ([(*η* ^5^-Cp*)Ir (L^L)Cl]PF_6_), cyclometallic Ir^Ⅲ^ complex have appropriate fluorescence and does not conflict with the commonly used tissue probes, facilitating the study of their tissue targeting. Classical cyclometallic Ir^Ⅲ^ complexes are usually expressed as [(ppy)_2_Ir (L^L)]^0/+^, where ppy is 2-phenylpyridine ligand, and L^L represents all kinds of chelating ligands. Reports have confirmed that cyclometallic Ir^Ⅲ^ complexes are sensitive to the change of microenvironment, e.g., viscosity, oxygen, and pH value. Aided by careful structural adjustment and molecular design, cyclometallic Ir^Ⅲ^ complexes can accumulate in specific subcellular organs, including lysosomes, mitochondria, cytoplasm, endoplasmic reticulum, etc., to achieve anticancer activity and bioimaging applications at the subcellular levels.

Lysosome are dynamic polymorphic organelles that contain a large number of histones and acid hydrolase (pH: 4.5–5.5), which can degrade and decompose biomacromolecules that enter cells through phagocytosis, autophagy, and endocytosis and maintain intracellular cholesterol and energy stability (Luzio et al., 2007; Saftig and Klumperman 2009). The lysosomal membrane is an integrating glycoprotein, a physiological barrier between the matrix and cytoplasm of the lysosome, which can prevent cell self-degradation (Eriksson et al., 2020). Once damaged, hydrolytic enzymes in lysosome are released, leading to cell death or apoptosis (Goddard and Reymond 2004; Kwon et al., 2005; Beija et al., 2009; Quang and Kim 2010; Wang and Wong 2011; Serrano-Puebla and Boya 2016; Ma et al., 2018; Ma et al., 2019a; Ma et al., 2019b). Moreover, the lysosomal content is higher in tumor tissue. Therefore, lysosomes are considered potential targets for the selective killing of tumor cells, and thus, the vigorous development of such drugs has become one of the main areas of cancer research. In addition, it has been reported that heteroatoms containing lone electron pairs are introduced into the peripheral ligands of Ir^Ⅲ^ complexes, such as benzimidazole (Wang et al., 2017), morpholine (Qiu et al., 2016), ferrocene (Ge et al., 2019), rhodamine (Ma et al., 2019a) and imine-*N*-heterocyclic carbine (Yang et al., 2019), facilitate the lysosomal target, which laied a basis for the design of lysosomal targeted cyclometallic Ir^Ⅲ^ complexes.

**FIGURE 1:**
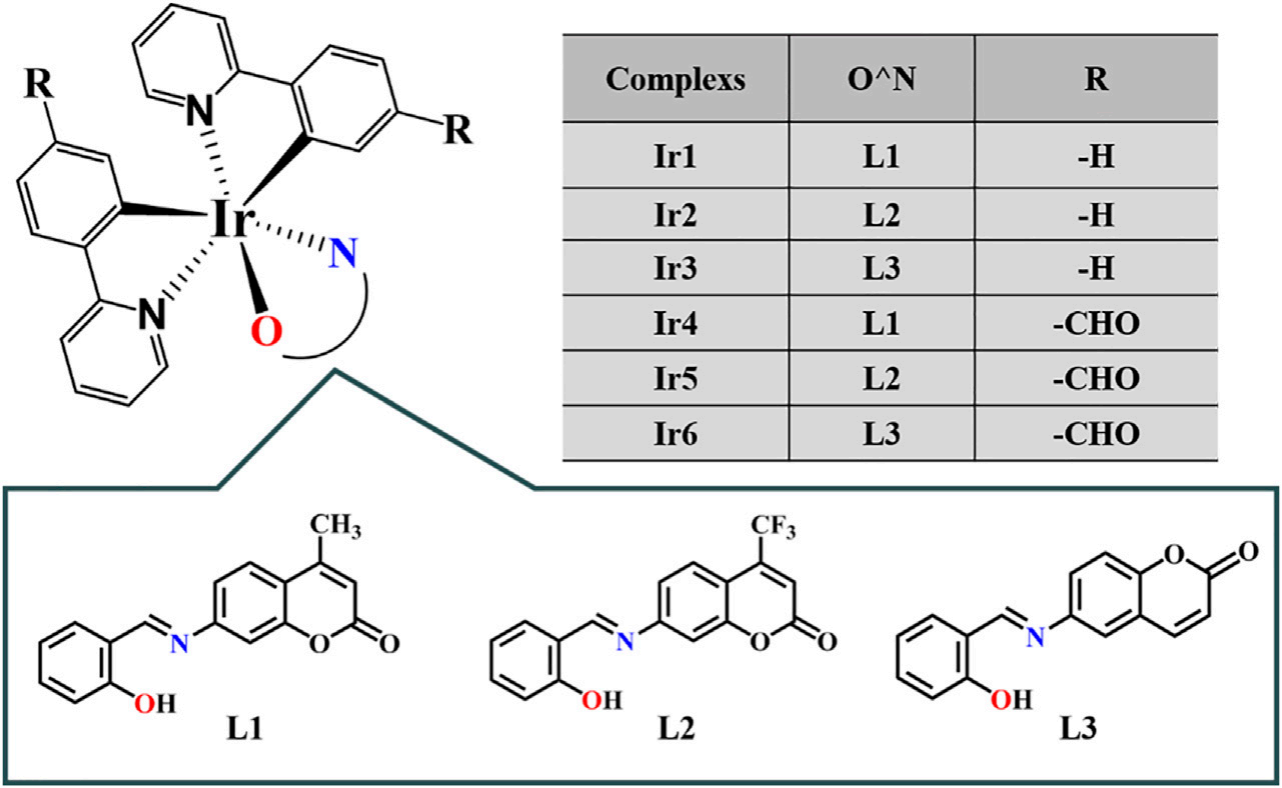
Structures of coumarin-modified cyclometallic Ir^Ⅲ^ Schiff base complexes.

Coumarin, widely found in nature, can interact with various enzymes and receptors, such as kinases, telomerase, aromatase, sulfatase, monocarboxylic acid transporters, and carbonic anhydrase, among others, thus acting as an anticoagulant (Poole and Poole 1994), antibacterial (Rosselli et al., 2009), antioxidant (Whang et al., 2005), anti-inflammatory (Piller 1975), and anticancer drugs (Luo et al., 2011). In addition, coumarin derivatives have various feasible cancer treatment targets, including mitosis, reactive oxygen species regulation, cell cycle arrest, telomerase, and angiogenesis (Bedair et al., 2000; Thati et al., 2009). Coumarin can selectively treat tumors by regulating various cellular signaling pathways (Finn et al., 2001; Finn et al., 2002). Schiff base compounds have a wide range of biological activities, such as immune regulation and antiviral, antibacterial, and antitumor effects (Bian et al., 2020; Sun et al., 2021). In view of the application prospect of coumarin derivatives and cyclometallic Ir^Ⅲ^ complexes in the field of anticancer, series of coumarin-appended cyclometallic Ir^Ⅲ^ salicylaldehyde Schiff base complexes ([(ppy)_2_Ir(O^N)] (Ir1-Ir3)/[(ppy-CHO)_2_Ir(O^N)] (Ir4-Ir6), Figure 1) were designed and synthesized. Compared with coumarin and Ir^Ⅲ^ complex monomers, target complexes show the better anticancer activity, especially for Ir2. We demonstrate that Ir2 can target lysosomes and trigger lysosome-mediated apoptosis in A549 cell line. Compared with BEAS-2B cells, we found that the cell cycle arrest, the intracellular ROS levels and the proportion of mitochondrial membrane depolarized cells are more obvious for A549 cells after incubated with Ir2, and eventually inducing more apoptosis. These results explain why these complexes are selective between A549 and BEAS-2B cells.

## 2 Results and Discussion

### 2.1 Synthesis and Characterization

**SCHEME 1:**
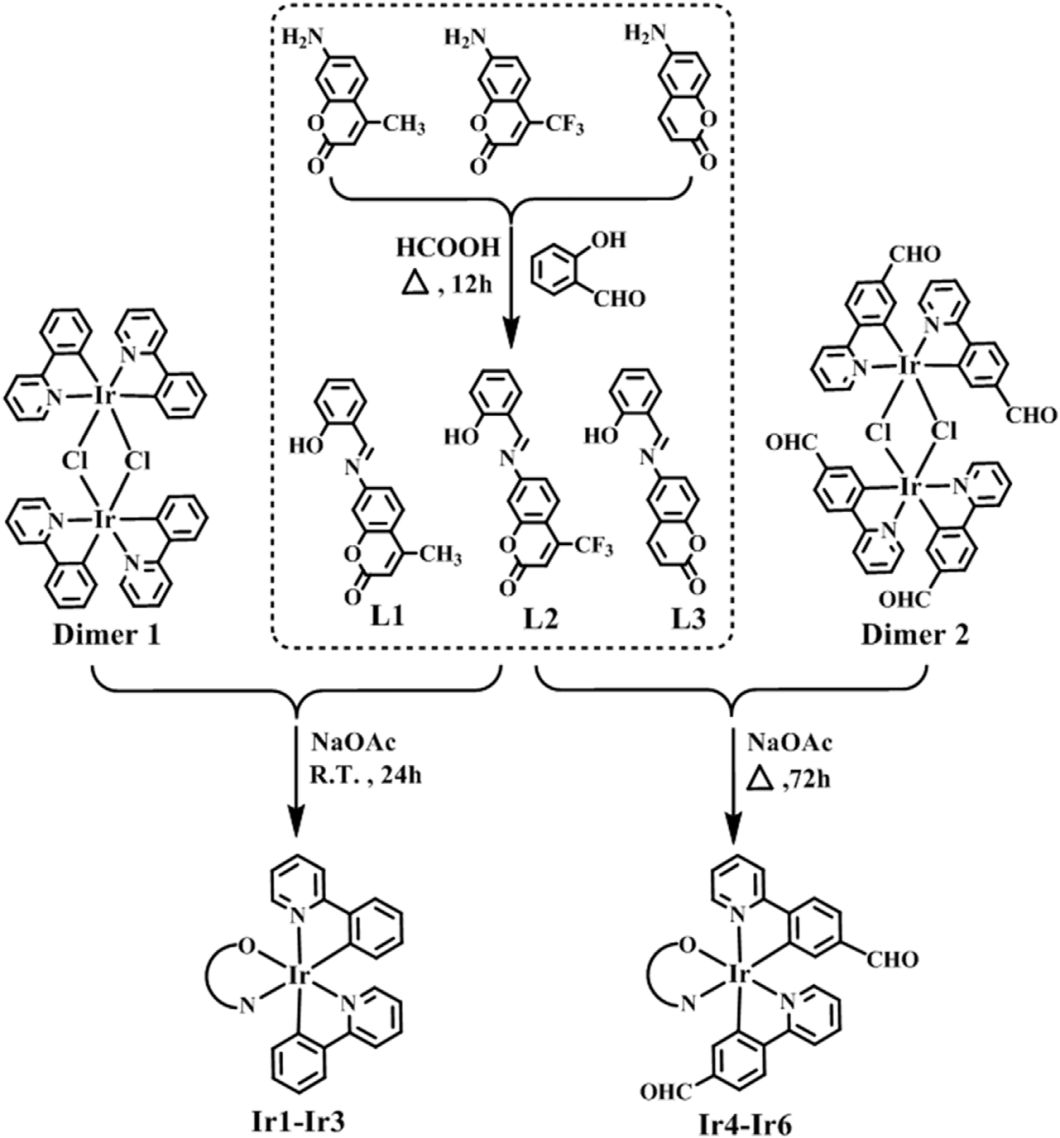
Synthesis process of coumarin-appended cyclometallic Ir^III^ complexes (Ir1-Ir6).

Coumarin-modified neutral cyclometallic Ir^Ⅲ^ salicylaldehyde Schiff base complexes [(ppy)_2_Ir(O^N)] (Ir1-Ir3)/[(ppy-CHO)_2_Ir(O^N)] (Ir4-Ir6) were obtained by the reaction of dinuclear dichloro-bridged Ir^Ⅲ^ precursors [(ppy)_2_IrCl]_2_/[(ppy-CHO)_2_IrCl]_2_ (ppy: 2-phenylpyridine, ppy-CHO: 4-(2-pyridine)-benzaldehyde) with coumarin-appended salicylaldehyde Schiff base chelating pro-ligands (O^N, L1-L3) through room temperature (R.T.) reaction and reflux reaction, respectively (Scheme 1). Schiff base pro-ligands were obtained by the condensation reaction of salicylaldehyde with 6-aminocoumarin or 7-aminocoumarin derivatives. Ir1-Ir6 are newly synthesized and obtained in 81–87% yields, and the structures are also established by spectroscopic and analytical methods, including FT-IR spectrum (Fourier transform-infrared), ^1^H, ^13^C and ^19^F NMR (nuclear magnetic resonance spectrum), ESI-MS (electrospray ionization mass spectrometry) and elemental analysis (Supporting Information). The tensile vibration absorption peaks of C=O and C=N were listed in 1720–1750 cm^−1^ and 1,600 cm^−1^ respectively. Deuterated reagent DMSO (2.50 ppm) and CDCl_3_ (7.26 ppm) were utilized as the solvent for NMR spectra. The peak positions of hydrogen atoms for methyl on coumarin unit (Ir1 and Ir4) are at ∼2.3 ppm, 6.0–9.0 ppm for the aromatic nucleus (coumarin and benzene), and ∼9.6 ppm for the aldehyde hydrogen atoms (Ir4-Ir6) in ^1^H NMR. The carbon on coumarin’s methyl group (Ir1 and Ir4) is at 18.58 and 18.56 ppm, 110–169 ppm for the benzene ring and coumarin, and ∼190.3 ppm for the aldehyde carbon atoms (Ir4-Ir6) in ^13^C NMR spectra. The fluorines on trifluoromethyl (Ir2 and Ir5) were listed in –64.9 ppm in ^19^F NMR spectra. The results of ESI-MS are consistent with the conclusions of theoretical calculations (combined with Na^+^).

**FIGURE 2:**
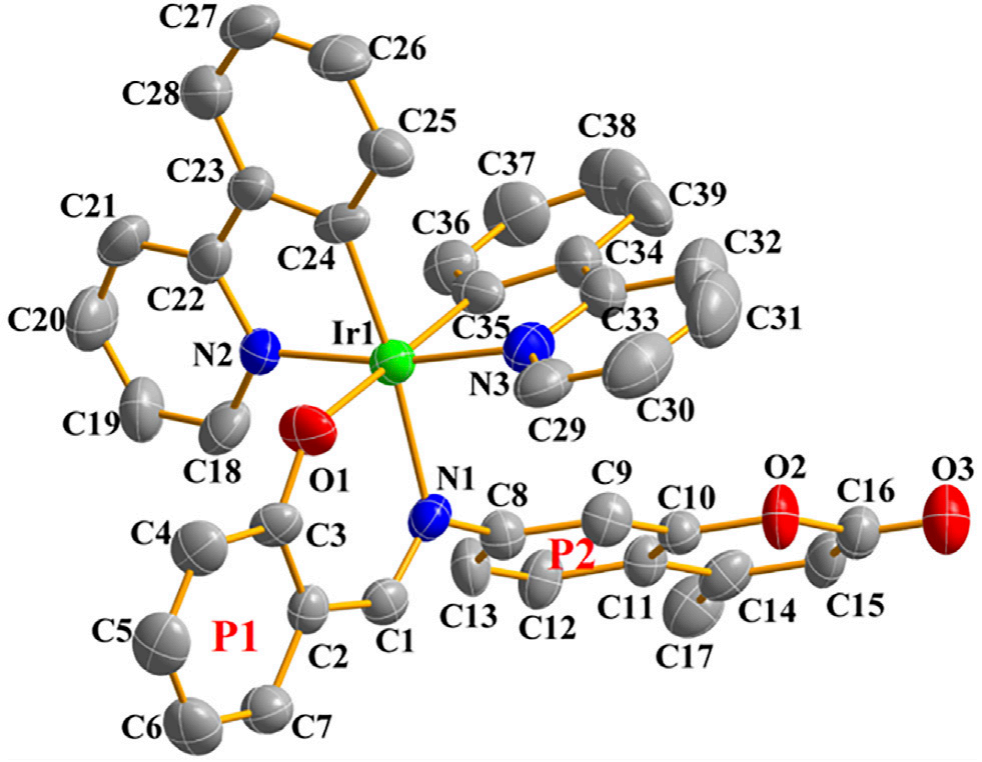
Single-crystal ORTEP diagram of Ir1 (50% thermal ellipsoids, hydrogen atoms were omitted for clarity). CCDC number 2158102.

Single crystals suitable for X-ray diffraction analysis were obtained by solvent diffusion method: *n*-Hexane slowly diffused into a dichloromethane solution of Ir1. The crystal structure and the specific crystal parameters are shown in Figure 2 and Supplementary Tables S1, S2. Ir1 crystallizes as a monoclinic system and P2(1)/c space group. The central iridium (III) ion shows a pseudo-octahedral coordination mode with the angles of O1-Ir1-N1, O1-Ir1-N2 and N1-Ir1-N3 changed from 86.6° to 91.9°, and the terminal coumarin unit exhibiting a classical planar configuration. The distance of Ir1-O1 (2.151 Å), Ir1-N1 (2.141 Å), Ir1-N3 (2.035 Å) and Ir1-N2 (2.045 Å) are almost the same. Salicylaldehyde group (P1) is almost coplanar with Schiff base unit (C=N) with a minor dihedral angle of 8.51°, however, which exhibits a large angle (63.55°) with coumarin unit (P2), indicating the poor coplanarity of whole Schiff base chelating ligand because of the large steric hindrance.

### 2.2 Cell Cytotoxicity Assay

MTT assay was utilized to evaluate the anticancer activity of Ir1-Ir6 toward A549 cells (human non-small cell lung cancer cells, one of the deadliest types of cancer in both developing and developed countries) along with cisplatin (metal anticancer drug widely used in clinic) as a control and compared with the cytotoxicity against noncancerous BEAS-2B cells (epithelial cells isolated from pathological sections of normal bronchial epithelium from non-cancerous individuals). The half maximal inhibitory concentration (IC_50_) values are shown in Table 1. Ir1-Ir6 show favorable anticancer activity compared to coumarin-unappended cyclometallic Ir^Ⅲ^ salicylaldehyde Schiff base complex (Ir7, Supplementary Scheme S1) and salicylaldehyde-coumarin Schiff base pro-ligands (L1-L3, IC_50_: >100 *μ*M), which is comparable to or significantly better than that of cisplatin, especially for Ir2, confirming the excellent anticancer effect of Ir^Ⅲ^ complex and coumarin. Additionally, the substitution sites of coumarin (6- and 7-) have little effect on these complexes, however, the peripheral substituents show a significant effect on the structure−activity. Among them, the introduction of electron-absorbing group (formyl) on phenylpyridine (ppy) group obviously weakens the activity, however, the anticancer activity is effectively improved by the introduction of trifluoromethyl (electron-absorbing group) on coumarin compared with methyl (electron-donating group), which providing a structural basis for the optimal design of such complexes. Even more, Ir2 and Ir5, exhibiting the optimal bioactivity among these complexes, possess less cytotoxicity to BEAS-2B cells compared to cisplatin, which revealing the specificity of these complexes. In addition, the data is even better than the reported traditional cyclometallic Ir^III^ complexes (usually depicted as [(ppy)_2_Ir (L^L)], L^L = N^N, C^N, C^O, O^N, P^P, etc., Supplementary Table S3).

**TABLE 1:** IC_50_ (*μ*M) values of the complexes toward selected cells for 24 h.

Complexes	IC_50_ (*μ*M)	
	A549 Cells	BEAS-2B Cells
Ir1	54.24 ± 5.35	—
Ir2	15.45 ± 0.14	78.14 ± 2.41
Ir3	37.84 ± 10.78	—
Ir4	72.18 ± 2.54	—
Ir5	20.99 ± 1.41	83.23 ± 4.11
Ir6	40.38 ± 0.28	—
Ir7	>100	—
Cisplatin	21.3 ± 1.7	38.4 ± 2.8

The water solubility of drugs is a necessary condition for their absorption, transport and diffusion *in vivo* (Kallinteri and Antimisiaris 2001). Generally, cyclometallic Ir^Ⅲ^ complexes possess low solubility and good stability in aqueous solutions. The stability of Ir2 and Ir5 in a DMSO/H_2_O (1:4, v/v) solution was detected based on its UV-vis absorption spectra over 8 h at 298 K, and the fractions of DMSO ensured the dissolution of the targeted complexes (Supplementary Figure S6). The absorption of Ir2 revealed a significant change over time compared to that with Ir1 and Ir5, indicating obvious hydrolytic properties. The time-dependent hydrolysis conformed to pseudo-first-order kinetics with a hydrolysis rate constant and half-life of 0.0036 min^−1^ and 192.54 min, respectively. Considering the optimal activity of Ir2, its excellent hydrolysis performance has a great effect on the anticancer activity of these complexes.

### 2.3 Protein Binding Study

**FIGURE 3:**
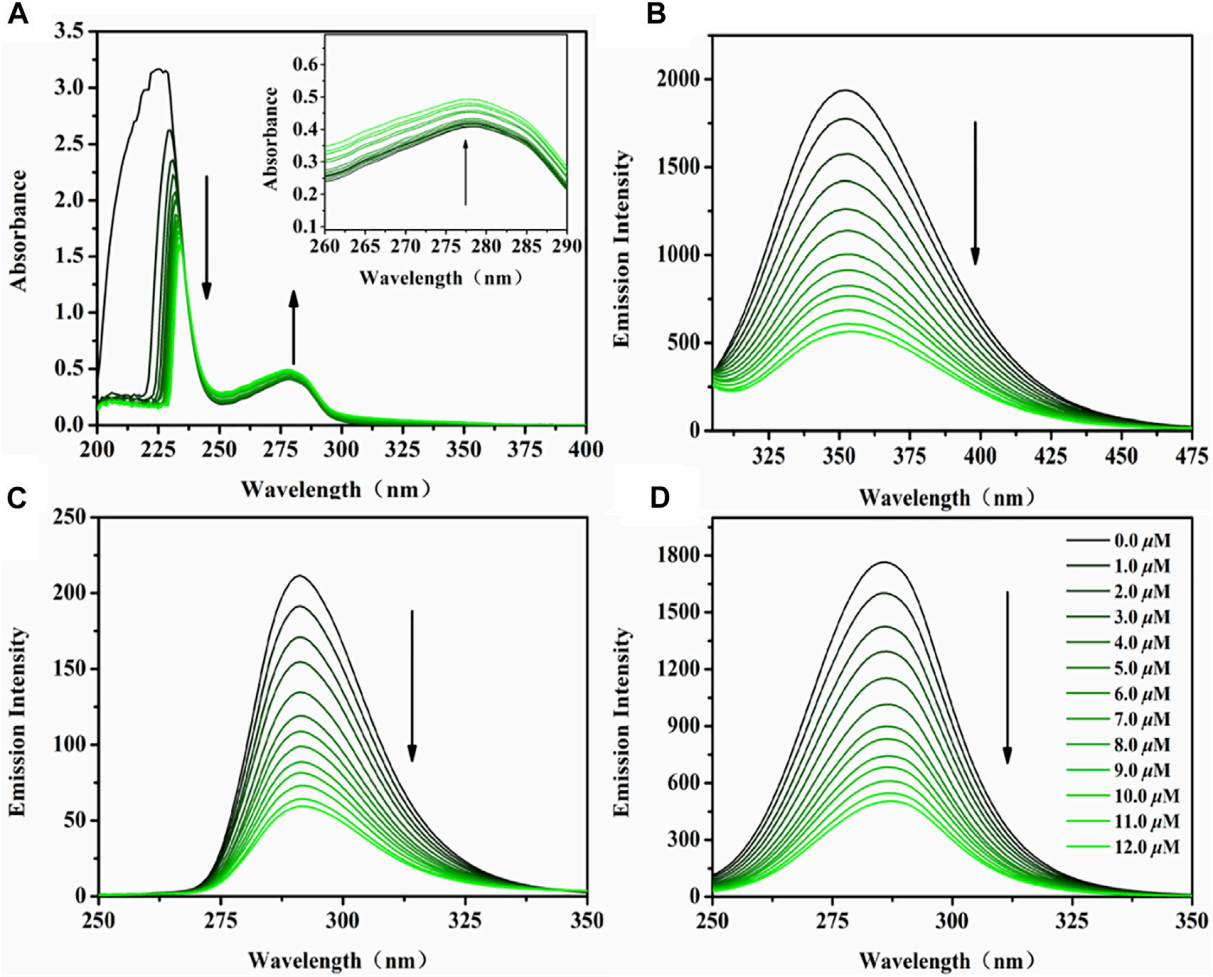
**(A)** UV-vis spectra of BSA (10 *μ*M) hatched with Ir2 (0–12 *μ*M) in Tris-HCl solution. Inset: Wavelength from 260 to 290 nm; **(B)** The emission spectra of BSA (10 *μ*M) hatched with Ir2 (0–12 *μ*M) in Tris-HCl solution; Synchronous fluorescence spectra of BSA (10 *μ*M) hatched with Ir2 (0–12 *μ*M) at a wavelength interval of 15 nm **(C)** and 60 nm **(D)**; Arrow shows the intensity change upon increasing Ir2 concentration.

Serum albumin (SA), the main protein component of serum total protein, is the main carrier of various metal ions, drugs and nutrients. Its combination with anticancer drugs is an important parameter to evaluate the drug utilization in organisms (Doweiko and Nompleggi 1991). It has been reported that a variety of drugs can be transported to the target location after binding with serum albumin in the blood, so as to exert their efficacy (Dömötör et al., 2013). Bovine serum albumin (BSA), the most extensively studied serum albumin protein because of its structural homology with human serum albumin (HSA), was utilized as a model to investigate the protein-binding mechanism in this study. The UV-vis spectra of BSA before and after the addition of the targeted complexes in Tris-HCl buffer solution (pH ∼7.2) are shown in Figures 3A, Supplementary Figure S7. A marked reduction was observed at 227 nm (the absorption of BSA) accompanied by a slight red shift (∼7 nm, induced by water) after the addition of Ir1-Ir6, which indicates the conformational change of BSA, reduction of *α*-helix structure content, and loosening of the protein conformation (Wei et al., 2020). Meanwhile, the intensity at 278 nm showed a slight increase, which reflects the changes in the BSA microenvironment (three major aromatic acids residues: tryptophan, phenylalanine and tyrosine) after hatching with these Ir^III^ complexes. Fortunately, tyrosine and tryptophan residues were reflected through synchronous fluorescence spectra based on the wavelength interval (*Δλ*, 15 and 60 nm, respectively), Figures 3C,D, Supplementary Figures S8, S9 (Pacheco and Bruzzone 2013). After the addition of Ir1-Ir6, the fluorescence intensity decreased significantly accompanied by a minor blue shift (∼2 nm) at *Δλ* = 15 nm, however, no changes occurred at the wavelength interval of 60 nm. The data indicate a conformational change in BSA, and the polarity around the tyrosine residues was increased (He et al., 2018).

The interaction between BSA and Ir1-Ir6 were studied by fluorescence measurement at room temperature (Zhang et al., 2008). Fluorescence spectra were recorded in the range of 290–450 nm upon excitation at 280 nm, Figures 3B, Supplementary Figure S10. A significant decrease of the fluorescence intensity of BSA at 351 nm was found upon increasing complexes concentration. The binding number (*n*), binding constant (K_b_) and quenching rate constant (*K*_*q*_) between BSA and complexes are obtained by Scatchard equation (Log[(F_0_-F)/F] = LogK_b_ + nlog[*Q*]) and Stern–Volmer equation (F_0_/F = 1+K_sv_[*Q*]= 1+K_q_τ_0_[*Q*]) fitting (Supplementary Figures S11, S12 and Table 2) (Cheng 2012). The values of *K*_*q*_ are nearly one order of magnitude higher than that of a pure dynamic quenching mechanism (<2.0 × 10^12^ M^−1^ s^−1^), which indicating a static quenching mechanism between Ir1-Ir6 and BSA (Esteghamat-Panah et al., 2017). Binding number (*n*) is almost the same (∼1), which corresponds to the conclusion that these complexes can only influence the polarity around the tyrosine residues in BSA. Above all, these studies suggest that SA can be used as a preeminent carrier for these Ir^III^ complexes in plasma, then affecting the anticancer activity.

**TABLE 2:** Values of K_b_, K_q_ and n for complexes Ir1-Ir6 at 310 K.

Complexes	K_b_ (10^4^ M^−1^)	*N*	*K*_*q*_ (10^13^ M^−1^ S^−1^)
Ir1	9.87	1.29	2.34
Ir2	9.00	1.03	1.08
Ir3	8.89	1.03	1.04
Ir4	9.54	1.29	2.26
Ir5	8.64	1.08	1.13
Ir6	6.25	1.28	1.33

### 2.4 Cellular Uptake and Localization Investigation

To determine the cellular uptake mechanism and target sites, suitable luminescence properties for drugs are necessary. UV-vis and emission spectra of Ir2 and Ir5 (selected complexes, which showing the best activity among these complexes) in 20% dimethyl sulfoxide (DMSO) and 80% phosphate buffered solution (PBS, pH ∼7.2) were investigated, Supplementary Figure S13. Ir2 and Ir5 exhibit a ligand absorption bands (π-π*) at 250–350 nm and a metal-ligand charge transfer band at 400–500 nm induced by π*→π charge transition. Interestingly, although Ir2 and Ir5 show almost the similar main emission peaks (∼505 nm) using the same excitation wavelength (405 nm), but the fluorescence intensity of Ir5 is much higher than that of Ir2 because of the introduction of the formyl group on phenylpyridine (ppy) ligands, including Ir5 exhibiting the longer fluorescence lifetime (1.768 *μ*s), Supplementary Figure S13C. In addition, except for the main emission peak (508 nm), Ir2 also has a relatively weak fluorescence peak at 642 nm in DMSO/PBS solution, however, only a fluorescence peak (507 nm) in pure DMSO, which further confirming the hydrolytic nature of Ir2.

**FIGURE 4:**
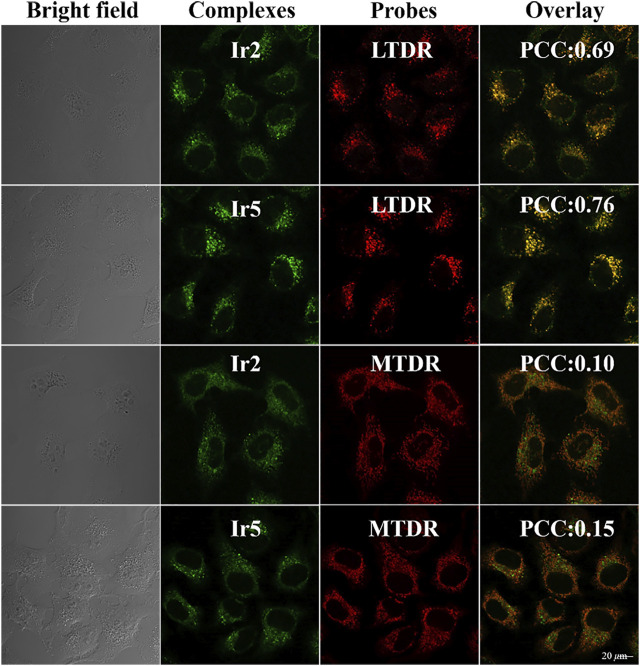
Confocal microscopy images of A549 cells hatcheded with Ir2 or Ir5 (10.0 *μ*M, 1 h), LTDR (75 nM, 0.5 h) and MTDR (500 nM, 0.5 h) at 37 °C (Ir2 and Ir5 excitation at 405 nm; Emission: 430–580 nm; LTRD and MTDR excitation at 594 and 644 nm; Emission: 630 ± 30 nm and 690 ± 30 nm).

Based on these favorable fluorescence properties, the subcellular localization of Ir2 and Ir5 in A549 cells was detected using confocal microscopy. Lyso Tracker Red DND-99 (LTDR) and Mito Tracker^®^ Red CM-H2XRos (MTDR) were used as lysosomal and mitochondrial fluorescent probe, respectively (Humpert et al., 2015; Raha et al., 2020). As shown in Figure 4, lysosomes were the main target sites of complexes after 1 h of incubation, and Pearson’s colocalization coefficient (PCC) for Ir2 and Ir5 were 0.69 and 0.76, respectively. However, the PCC for the mitochondria was only 0.10 and 0.15. Therefore, after effectively penetrating the A549 cells, these complexes were mainly localized in the cytoplasm and specifically targeted lysosomes. Moreover, there was no immediate abnormal cell death after incubated with Ir2 or Ir5, which facilitated the timely tracking of intracellular lysosomal morphological changes.

**FIGURE 5:**
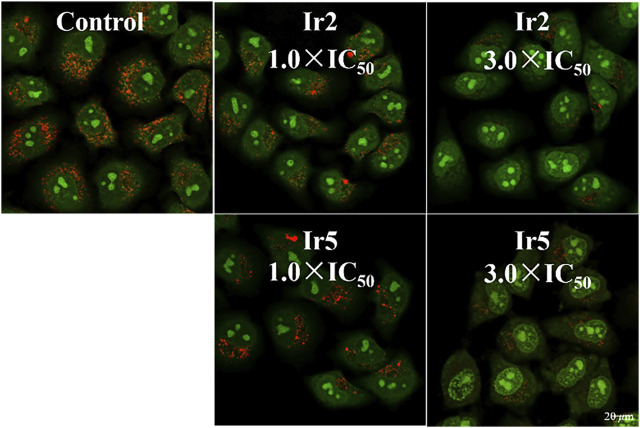
Confocal microscopy images of A549 cells stained with AO (5 *μ*M, 15 min) after hatched with Ir2 or Ir5 at 310 K. AO excitation at 488 nm; Emission: 510 ± 20 nm (green) and 625 ± 20 nm (red).

Lysosomes are acidic intracellular organelles that can break down biological macromolecules, including proteins, nucleic acids, and polysaccharides. Once the damages amounts to some extent, hydrolase is be released, digesting the whole cell and promoting apoptosis (KÅGedal et al., 2001). Acridine orange (AO) is always utilized to detect the integrity of lysosomal membrane, which emits a red fluorescence in an intact lysosome, and a green fluorescence incytoplasm or nucleus (Qian et al., 2013). In this study, A549 cells were stained with AO (5 *μ*M, 15 min) after hatched with Ir2 or Ir5 (10 *μ*M) for 6 h, and to investigate the lysosomal membrane permeabilization (LMP), Figure 5. The red fluorescence decreased after treatment with Ir2 or Ir5 (1.0 × IC_50_) for A549 cells compared to that with the control. Significant lysosomal damage occurred at a high concentration (3.0 × IC_50_), which could be observed by the naked eye. These results confirmed that these complexes mainly target the intracellular lysosomes, damage the integrity of lysosome, induce cell death, and exhibit bioactivity.

It is well-known that a favorable cell-penetrating ability is closely related to the antitumor activity of drugs. In general, there are two main cellular uptake mechanisms for organometallic drugs: non-energy-dependent mechanism (passive transport and free diffusion) and energy-dependent mechanism (active transport and endocytosis). In this study, A549 cells were incubated with chloroquine (endocytosis regulator, 50 *μ*M) and chlorocyanochlorobenzene (CCCP, metabolic inhibitor, 50 *μ*M) to determine the cellular uptake mechanism after hatching with Ir2 or Ir5 at 277 K or 310 K for 1 h, respectively (Supplementary Figure S14). As shown, except for the incubation at 277 K, other groups demonstrated that complexes could enter A549 cells smoothly, indicating an energy-dependent mechanism for these complexes.

### 2.5 Anticancer Mechanism Assay

**FIGURE 6:**
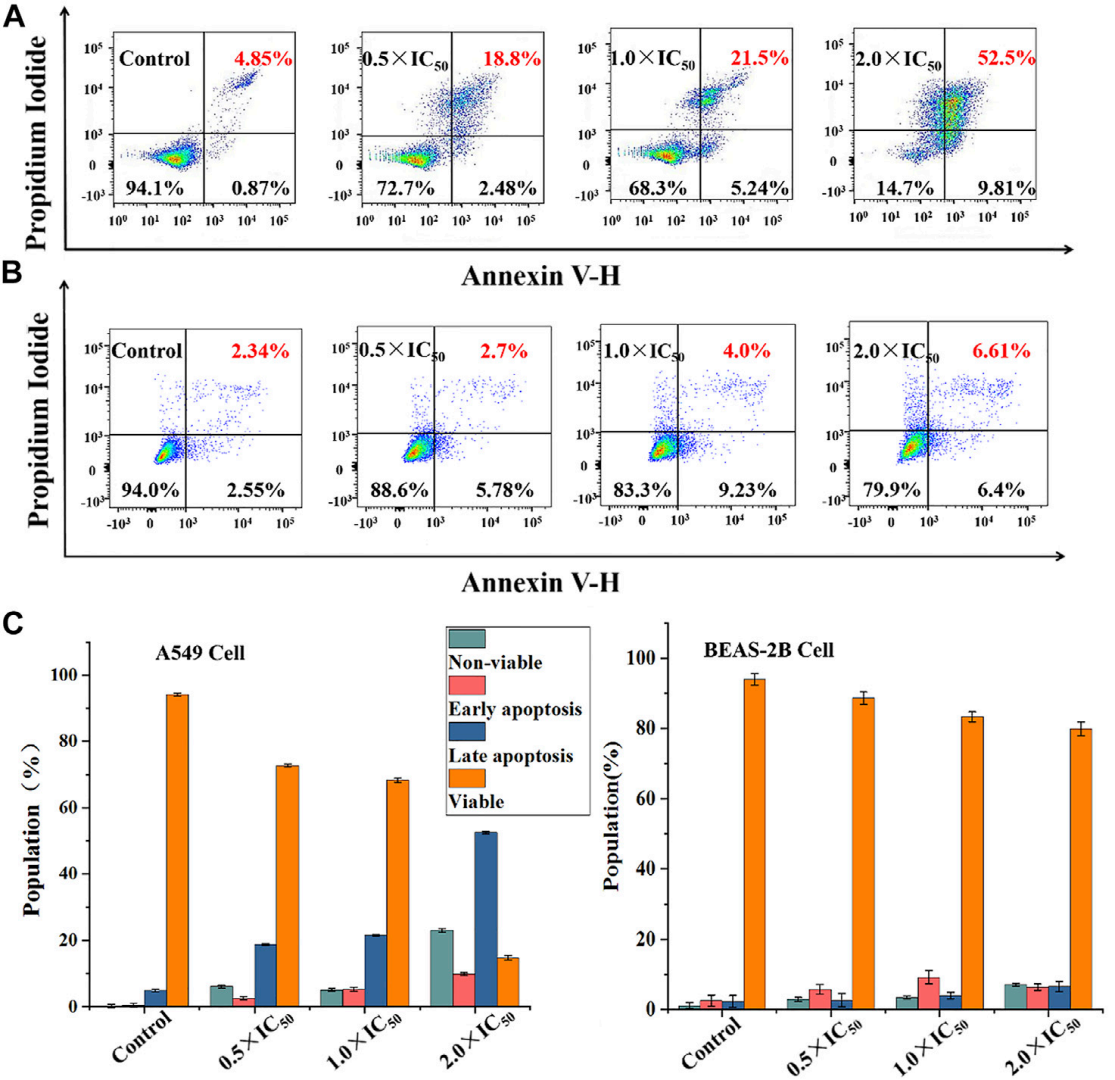
The apoptosis assay was evaluated after A549 cells **(A)** and BEAS-2B cells **(B)** hatched with Ir2 for 24 h, and the color dot plots mean that the cells were stained with Annexin V-FITC/PI; **(C)** The population of viable, apoptotic (early + late) and non-viable cells.

To better maintain the stability of intracellular microenvironment, apoptosis, the spontaneous and orderly death of cell controlled by genes, is a common pathway, and most metallic anticancer complexes can eliminate tumor cells through it (Novohradsky et al., 2014). Generally, apoptosis exhibits a temporal sequence, which is characterized by the destruction of mitochondrial membrane potential, nuclear rupture and condensation. Considering the extensive and important role of apoptosis in biology and medicine (Schwieger et al., 2001; Jan and Chaudhry 2019), the fluorescein labeled Annexin V-FITC as probe and propyl iodide (PI) as nucleic acid dye was used to detect apoptotic cells and dead cells using flow cytometry. As shown in Figure 6, Supplementary Tables S4, S5, when A549 cells were treated with Ir2 using a gradient concentration, the proportion of late apoptotic cells increased from 18.8 to 52.5%. The survival rate was 94.1% for the control under the same conditions, confirming the anticancer effect of apoptosis in these complexes. Additionally, the proportion of apoptotic BEAS-2B cells, including early apoptosis and late apoptosis, only increased by 8.12% when the concentration of Ir2 was changed from 0.5 × IC_50_ to 2.0 × IC_50_. This result corresponds to the data of the cytotoxicity evaluation; specifically, Ir2 showed selectivity between A549 cancer cells and BEAS-2B normal cells.

**FIGURE 7:**
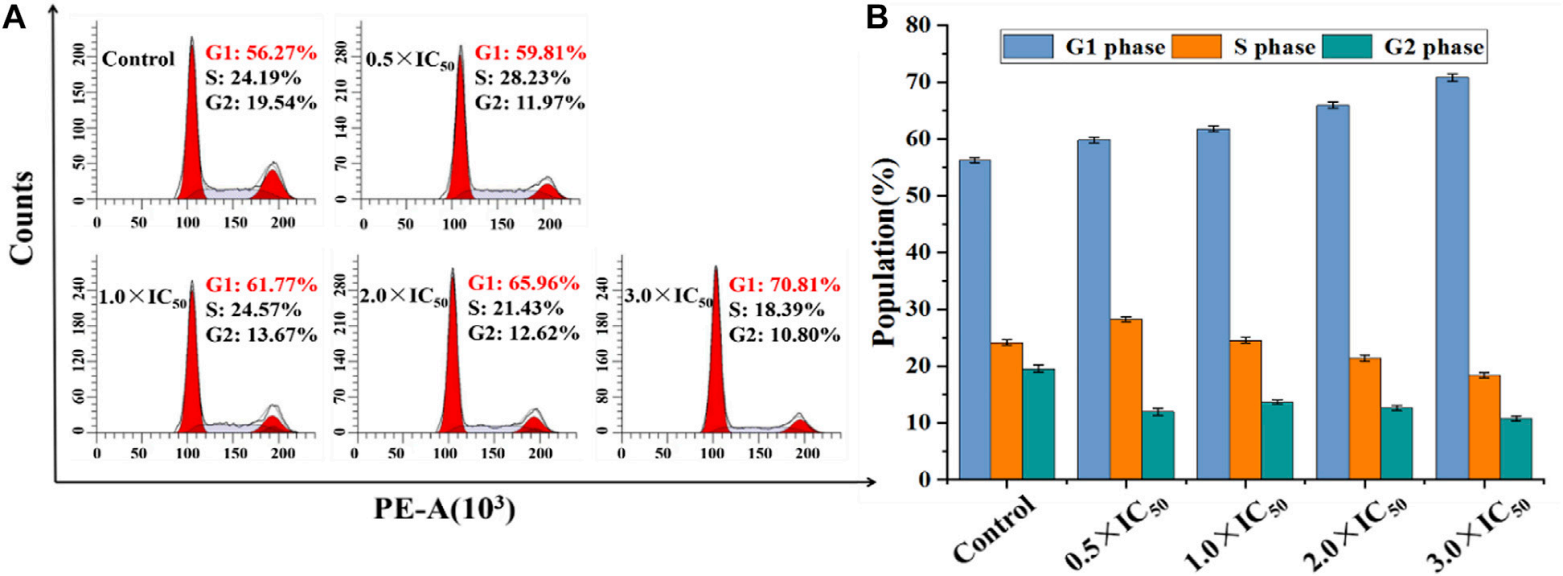
**(A)** Distribution of cell cycle of A549 cells hatched with Ir2 for 24 h. **(B)** Histograms of cell cycle data at each stage for A549 cells after hatched with Ir2.

Generally, organometallic anticancer complexes can induce cell dysfunction by destroying the cell cycle, eventually leading to apoptosis (Wang et al., 2014). A549 and BEAS-2B cells were stained by PI/RNase and treated with Ir2 at a gradient concentrations for 24 h to analyze the cell cycle arrest, and the data were listed in Supplementary Tables S6, S7. As shown in Figure 7, Supplementary Figure S15, the A549 cell arrest rate increased by 11.0% in G_1_ phase when the concentration of Ir2 changed from 0.5 × IC_50_ to 3.0 × IC_50_, however, this change was not evident in BEAS-2B cells. This confirms that these complexes might disrupt cell cycle (G_1_-phase) of A549 cancer cells, leading to apoptosis.

**FIGURE 8:**
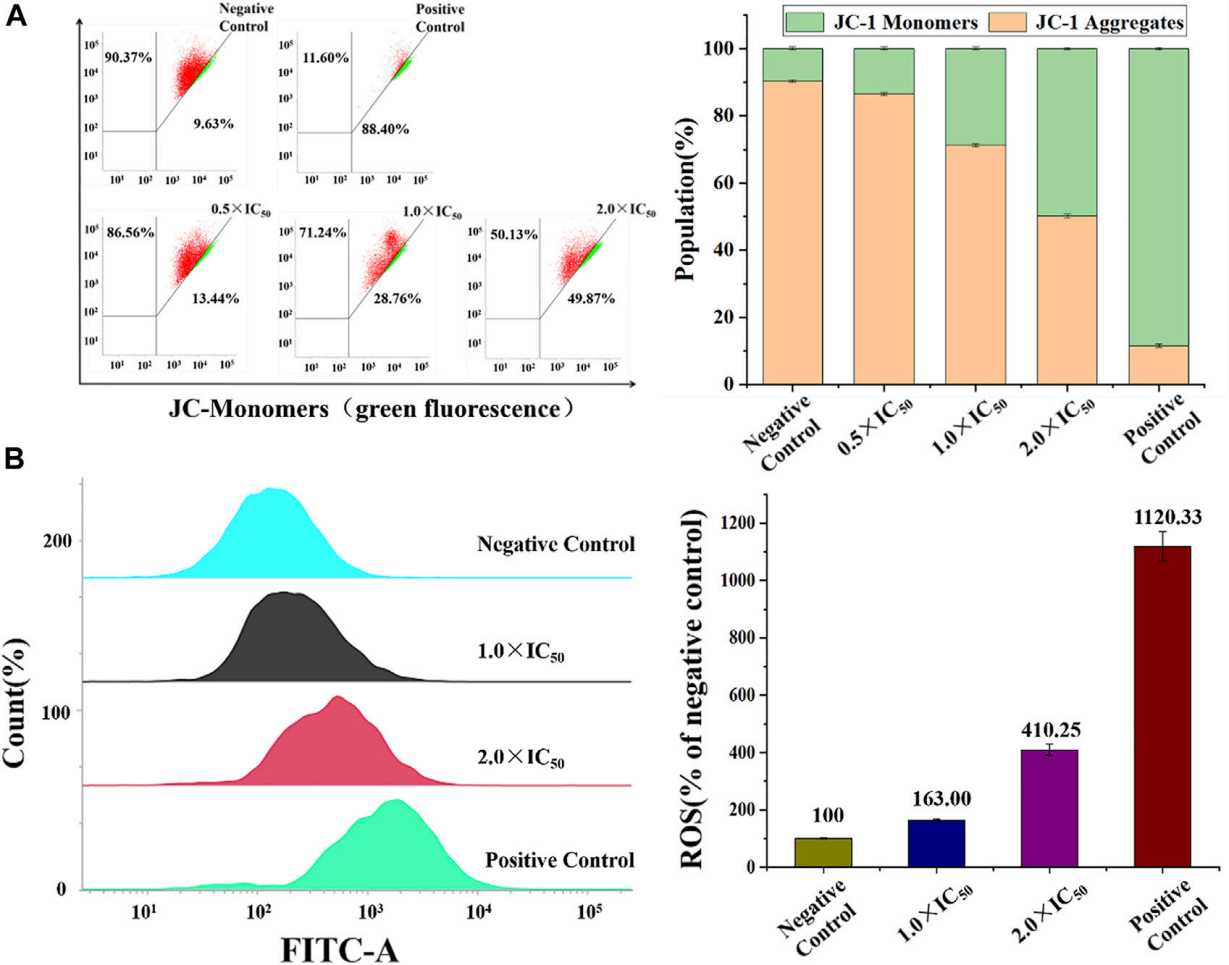
**(A)** The percentage of A549 cells (red) and depolarized A549 cells (green) in A549 cells after induced by Ir2 and stained with JC-1. **(B)** ROS induction of A549 cells hatched with Ir2 at the concentration of 1.0 × IC_50_ and 2.0 × IC_50_ for 24 h. Data are quoted as mean ± SD of two replicates.

Mitochondria, the main intracellular energy source, play an important role in the process of apoptosis. Disruption of mitochondrial membrane potential (MMP) is usually identified as the early signals of cell apoptotic. JC-1, a liphile cationic fluorescent dye, is generally used to detect the change of MMP, and it emits strong red fluorescence in the normal mitochondrial matrix and green fluorescence after depolarization activation in apoptotic cells (Antonicka et al., 2017). In this study, flow cytometry was utilized to analyze this change after the treatment of Ir2 at a gradient concentration. As shown in Figure 8A, Supplementary Tables S8, S9, the number of depolarized cells (green fluorescence) in A549 cells increased by 36.43% when the concentration increased from 0.5 × IC50 to 3.0 × IC50, suggesting a concentration-dependent mode. However, the number of BEAS-2B cells increased by only 20.89% (Supplementary Figure S16). These results indicate that complexes could lead to mitochondrial dysfunction and induce apoptosis. Considering the accumulation of these complexes in lysosomes, the existence of a lysosomal-mitochondria anticancer pathway is suggested. In addition, intracellular reactive oxygen species (ROS) is mainly produced and stored in mitochondria, and the accumulation of intracellular ROS can lead to apoptosis (Romero-Canelón et al., 2015). A549 and BEAS-2B cells were stained with DCFH-DA after incubated with Ir2 (1.0 × IC_50_ and 2.0 × IC_50_) for 24 h (Figure 8B). The intracellular ROS levels increased in a dose-dependent manner. Compared to those in the control, the value of ROS level increases by 310% at 2.0 × IC_50_ in A549 cells. ROS levels also increased in BEAS-2B normal cells, but this was not as obvious, as in A549 cells (Supplementary Figure S17).

**FIGURE 9:**
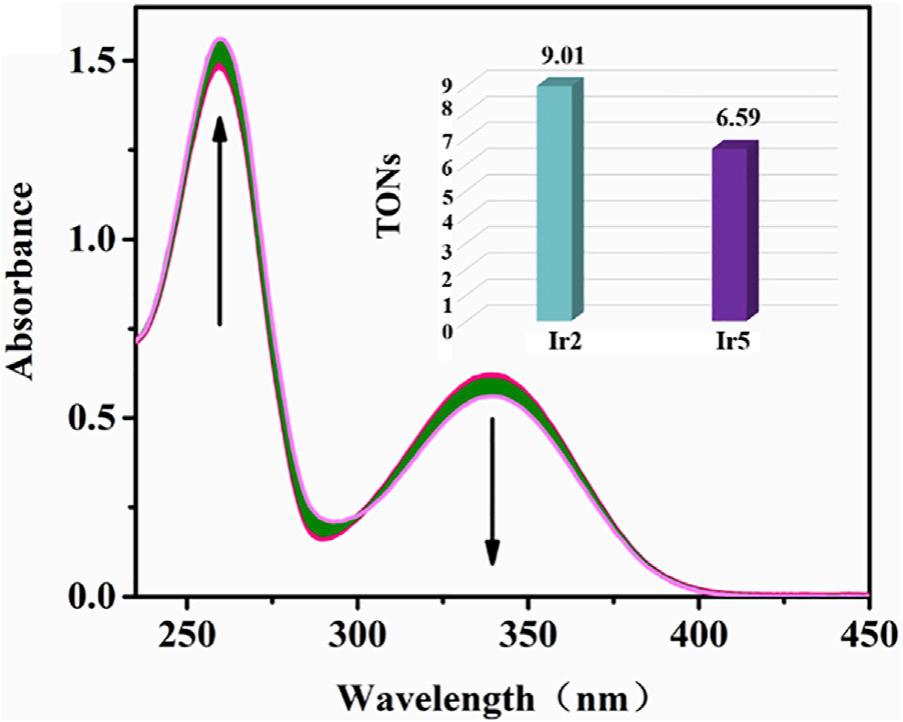
Reaction of Ir2 (1 *μ*M) and NADH (100 *μ*M) in 10% MeOH/90% H_2_O (v/v) solution detected by UV-vis spectra over 8 h. The arrow show the change of intensity over time; Inset: TONs of Ir2 and Ir5.

NADH, the reduced state of nicotinamide adenine dinucleotide, plays a key role in the regulation of energy production (Santidrian et al., 2013). The REDOX state of NADH is a control marker of the mitochondrial energy production chain, which is the best parameter for characterizing mitochondrial function *in vivo*. Reports have verified that organometallic complexes, including Ir(III), Rh(III), Os(II), and Ru(II), etc., could catalytic accelerate the conversion of NADH to NAD^+^ (oxidation state of NADH) and induce the increase of intracellular ROS levels (Millett et al., 2015). The catalytic efficiency of Ir2 or Ir5 (1 *μ*M) toward NADH (100 μM) in 10% MeOH/90% H_2_O (v/v) was measured by UV−vis absorption spectroscopy at 298 K, Figure 9, Supplementary Figures S10, S18. The absorption of 339 nm (the maximum absorbance of NADH) decreases gradually after the addition of Ir2 and Ir5, accompanied by the enhance of 259 nm (the absorbance of NAD^+^) (Liu et al., 2013). The catalytic efficiency can be intuitively expressed using the NADH oxidation turnover number (TON) change ratio at 339 nm Ir2 has the better biocatalytic performance with a TON value of 9.00 relative to that of Ir5, and this result was consistent with the trend shown by the MTT assay, confirming the relationship between intracellular ROS levels and the anticancer activity of these complexes. Especially, cyclometallic Ir^Ⅲ^ salicylaldehyde-Schiff base complexes can disturb the cell cycle, decrease MMP, accelerate the oxidation of NADH, lead to an increase in intracellular ROS levels, and eventually induce apoptosis.

## 3 Conclusion

In conclusion, we have synthesized a series of coumarin-modified cyclometallic Ir^Ⅲ^ salicylaldehyde Schiff base complexes. Compared with coumarin and Ir^Ⅲ^ complex monomers, target complexes had the better anticancer activity. Interestingly, these complexes exhibited selectivity towards A549 cells and BEAS-2B cells. The effect of peripheral substituents on the activity was evaluated. Compared to phenylpyridine, the introduction of an electron-withdrawing group (-CF_3_) on coumarin was more conducive to improving the anticancer activity of these complexes, which provided a basis for further structural optimization. Additionally, in contrast to the stability of the traditional cyclometallic Ir^Ⅲ^ complex, target complexes showed favorable hydrolytic properties and could bind to serum proteins in a static quenching mode for plasma transport. Additionally, cell cycle arrest, the levels of intracellular ROS, and the decrease in MMP in A549 cells were more obvious than those in BEAS-2B cells after incubated with Ir2, and this eventually induced apoptosis to a greater extent. These results explain why these complexes are selective between cancer and normal cells. Owing to suitable fluorescence, confocal detection confirmed that these complexes followed an energy-dependent cellular uptake mechanism, accumulated in lysosomes, and induced lysosomal membrane permeabilization, eventually leading to apoptosis. Coumarin-modified cyclometallic Ir^Ⅲ^ salicylaldehyde Schiff base complexes are thus potential organometallic anticancer drug and merit further study.

## 4 Experimental Section

### 4.1 Materials

Aniline, formic acid, 7-amino-4-trifluoromethylcoumarin, 7-amino-4-methylcoumarin, 6-aminocoumarin, 2-phenylpyridine, iridium trichloride hydrate (IrCl_3_·3H_2_O), crystalline sodium acetate, salicylaldehyde, 4-(2-pyridine)-benzaldehyde, and organic solvents (2-ethoxyethanol, methanol, ethanol, acetone, ethyl ether, dichloromethane, *n*-hexane etc.) were prepared through Rhea biotechnology Co. Ltd. (Xi’an). Fluorescent dye and related assay kits were purchased from Beyotime Biotechnology (Shang Hai). A549 and BEAS-2B cells were obtained from Shanghai Institute of Biochemistry and Cell Biology (SIBCB). Basic dimers of iridium ((ppy)_2_IrCl]_2_/[(ppy-CHO)_2_IrCl]_2_) and salicylaldehyde-coumarin Schiff base pro-ligands (L1-L3) and coumarin-unappended cyclometallic Ir^Ⅲ^ salicylaldehyde Schiff base complex (Ir7) were prepared using the literature procedures, and the specific synthesis methods and characterization data were shown in supporting information.

### 4.2 Synthesis of [(ppy)_2_Ir(O^N)] (Ir1-Ir3)

The general synthetic process is as follows: [(ppy)_2_IrCl_2_]_2_ (54 mg, 0.05 mmol), salicylaldehyde Schiff base ligands (L1/L2/L3, 28 mg/34 mg/27 mg, 0.10 mmol) and crystalline sodium acetate (33 mg, 0.40 mmol) were dissolved in methanol (40 ml) and stirred overnight at room temperature. The reaction was detected by thin layer chromatography (TLC, dichloromethane:methanol = 50:1 v/v). The distilled crude product was dissolved in dichloromethane to form a saturated solution, and *n*-hexane slowly diffused to obtain the final product. The characterization spectra of Ir1-Ir3 are shown in Supplementary Figures S1–S5, and the data are as follows:

[(ppy)_2_Ir(L1)] (Ir1) Yield: 33 mg (85%). Selected FT-IR (KBr): 3050 (C-H), 1730 (C=O), 1,600 (C=N), 1,440 (C-N), 1,140 (C-O). ^1^H NMR (500 MHz, CDCl_3_): *δ* 8.87 (d, *J* = 5.6 Hz, 1H), 8.81 (d, *J* = 5.7 Hz, 1H), 8.12 (s, 1H, HC = N), 7.88 (d, *J* = 8.1 Hz, 1H), 7.75–7.68 (m, 2H), 7.58 (d, *J* = 7.7 Hz, 1H), 7.51 (d, *J* = 8.0 Hz, 1H), 7.28 (dd, *J* = 8.3, 1.4 Hz, 1H), 7.14 (ddd, *J* = 9.6, 7.4, 1.4 Hz, 2H), 7.09–7.05 (m, 1H), 6.92 (dd, *J* = 15.5, 8.0 Hz, 2H), 6.87–6.82 (m, 1H), 6.72 (dd, *J* = 11.2, 4.8 Hz, 2H), 6.50 (td, *J* = 7.4, 1.2 Hz, 1H), 6.45–6.39 (m, 2H), 6.23 (d, *J* = 7.6 Hz, 1H), 6.13 (dd, *J* = 17.4, 4.3 Hz, 3H), 6.07 (s, 1H), 2.26 (d, *J* = 0.9 Hz, 3H, Ar-CH_3_). ^13^C NMR (126 MHz, CDCl_3_): *δ* 168.83, 168.37, 167.29, 160.98, 160.85, 154.35, 152.62, 151.95, 151.13, 150.06, 148.98, 148.39, 144.38, 143.91, 137.12, 136.83, 135.41, 134.66, 133.46, 132.49, 129.48, 129.14, 125.37, 123.87, 123.40, 123.25, 121.75, 121.52, 121.37, 121.15, 119.66, 119.10, 119.08, 117.89, 116.60, 113.72, 113.68, 111.04, 18.58 (Ar-CH_3_). ESI-MS (m/z): Calcd for C_39_H_28_O_3_N_3_Ir: 779.1760; Found: 780.1796 [M + H]^+^. Elem anal. Calcd for C_39_H_28_O_3_N_3_Ir: C, 60.14; H, 3.62; N, 5.39; O, 6.16%. Found: C, 60.42; H, 3.82; N, 5.31; O, 6.08%.

[(ppy)_2_Ir(L2)] (Ir2) Yield: 35 mg (83%). Selected FT-IR (KBr): 3040 (C-H), 1740 (C=O), 1,600 (C=N), 1,430 (C-N), 1,270 (C-F), 1,140 (C-O). ^1^H NMR (500 MHz, CDCl_3_): *δ* 8.86 (d, *J* = 5.6 Hz, 1H), 8.79 (d, *J* = 5.6 Hz, 1H), 8.13 (s, 1H, HC = N), 7.89 (d, *J* = 8.2 Hz, 1H), 7.76–7.70 (m, 2H), 7.58 (d, *J* = 7.8 Hz, 1H), 7.52 (d, *J* = 8.1 Hz, 1H), 7.31–7.28 (m, 1H), 7.18–7.12 (m, 2H), 7.07 (ddd, *J* = 15.8, 7.7, 4.2 Hz, 2H), 6.93 (d, *J* = 7.7 Hz, 1H), 6.85 (dd, *J* = 10.8, 4.0 Hz, 1H), 6.72 (dd, *J* = 10.6, 4.5 Hz, 2H), 6.61 (s, 1H), 6.52 (dd, *J* = 10.5, 4.2 Hz, 1H), 6.46–6.40 (m, 2H), 6.22 (d, *J* = 7.5 Hz, 1H), 6.13 (dd, *J* = 13.2, 8.2 Hz, 3H). ^13^C NMR (126 MHz, CDCl_3_): *δ* 168.80, 168.31, 167.41, 160.71, 158.93, 154.97, 153.45, 150.62, 149.84, 148.85, 148.42, 144.33, 143.84, 137.22, 136.94, 135.41, 134.90, 133.50, 132.50, 129.53, 129.29, 125.51, 124.34, 124.32, 123.92, 123.28, 121.83, 121.62, 121.48, 121.04, 119.97, 119.82, 119.16, 117.92, 114.24, 114.20, 113.89, 111.71, 110.07. ^19^F NMR (471 MHz, CDCl_3_): *δ* -64.85 (s). ESI-MS (m/z): Calcd for C_39_H_25_O_3_N_3_F_3_Ir: 833.1477; Found: 834.1509 [M + H]^+^. Elem anal. Calcd for C_39_H_25_O_3_N_3_F_3_Ir: C, 56.24; H, 3.03; N, 5.05; O, 5.76%. Found: C, 56.41; H, 3.22; N, 4.97; O, 5.69%.

[(ppy)_2_Ir(L3)] (Ir3) Yield: 33 mg (86%). Selected FT-IR (KBr): 3040 (C-H), 1730 (C=O), 1,600 (C=N), 1,430 (C-N), 1,140 (C-O). ^1^H NMR (500 MHz, CDCl_3_): *δ* 8.91 (d, *J* = 5.6 Hz, 1H), 8.81 (d, *J* = 5.6 Hz, 1H), 8.11 (s, 1H, HC = N), 7.89 (d, *J* = 8.1 Hz, 1H), 7.74–7.70 (m, 1H), 7.69–7.63 (m, 1H), 7.58 (d, *J* = 7.8 Hz, 1H), 7.47 (d, *J* = 7.5 Hz, 1H), 7.21–7.09 (m, 4H), 7.05 (dd, *J* = 9.5, 3.4 Hz, 1H), 6.97 (d, *J* = 7.4 Hz, 1H), 6.85 (t, *J* = 7.4 Hz, 1H), 6.75–6.66 (m, 3H), 6.54–6.46 (m, 2H), 6.40 (t, *J* = 7.3 Hz, 1H), 6.31–6.16 (m, 5H). ^13^C NMR (126 MHz, DMSO): *δ* 168.13, 168.03, 166.43, 162.66, 160.32, 152.51, 150.80, 150.63, 150.04, 148.13, 147.93, 145.41, 144.85, 144.41, 138.29, 138.06, 136.74, 134.73, 133.18, 132.56, 128.90, 128.70, 126.50, 124.36, 123.95, 123.34, 122.73, 122.03, 121.87, 121.22, 119.94, 119.72, 119.03, 117.67, 116.36, 115.42, 113.43, 55.38. ESI-MS (m/z): Calcd for C_38_H_26_O_3_N_3_Ir: 765.1603; Found: 766.1639 [M + H]^+^. Elem anal. Calcd for C_38_H_26_O_3_N_3_Ir: C, 59.67; H, 3.43; N, 5.49; O, 6.28%. Found: C, 59.88; H, 3.59; N, 5.39; O, 6.20%.

### 4.3 Synthesis of [(Ppy-CHO)_2_Ir(O^N)] (Ir4-Ir6)

The general synthetic process is as follows: Basic dimer of iridium ([(ppy-CHO)_2_IrCl_2_]_2_, 59 mg, 0.05 mmol), coumarin-salicylaldehyde Schiff base ligands (L1/L2/L3, 28 mg/34 mg/27 mg, 0.10 mmol) and sodium acetate (33 mg, 0.40 mmol) were refluxed in methanol (40 ml) for 72 h, and monitored by thin-layer chromatography (TLC, dichloromethane:methanol = 100:1, v/v). After vacuum distillation, the final complexes were obtained by slow diffusion of *n*-hexane into a saturated dichloromethane solution of the crude products. The characterization spectra of Ir4-Ir6 are shown in Supplementary Figures S1–S5. The data are as follows:

[(ppy-CHO)_2_Ir(L1)] (Ir4) Yield: 35 mg (83%). Selected FT-IR (KBr): 2980 (C-H), 1720 (C=O), 1,600 (C=N), 1,380 (C-N), 1,120 (C-O). ^1^H NMR (500 MHz, CDCl_3_): *δ* 9.59 (d, *J* = 4.0 Hz, 1H, HC = O), 9.57 (s, 1H, HC = O), 8.94 (d, *J* = 5.5 Hz, 1H), 8.88 (d, *J* = 5.1 Hz, 1H), 8.12 (d, *J* = 6.2 Hz, 1H, HC = N), 8.02 (d, *J* = 8.1 Hz, 1H), 7.86 (tdd, *J* = 8.2, 4.4, 1.2 Hz, 2H), 7.71 (t, *J* = 8.8 Hz, 2H), 7.37 (dd, *J* = 7.9, 1.4 Hz, 1H), 7.35–7.32 (m, 1H), 7.32–7.27 (m, 2H), 7.25–7.21 (m, 1H), 7.15 (d, *J* = 8.0 Hz, 2H), 6.99 (dd, *J* = 7.9, 1.4 Hz, 1H), 6.87 (d, *J* = 8.5 Hz, 1H), 6.69 (d, *J* = 8.6 Hz, 1H), 6.64 (d, *J* = 1.4 Hz, 1H), 6.61 (d, *J* = 1.3 Hz, 1H), 6.46 (t, *J* = 7.3 Hz, 2H), 6.22 (d, *J* = 8.4 Hz, 1H), 2.23 (s, 3H, Ar-CH_3_). ^13^C NMR (126 MHz, CDCl_3_): *δ* 193.16 (HC = O), 192.92 (HC = O), 167.12, 166.83, 166.60, 161.67, 160.42, 154.00, 152.82, 151.75, 150.82, 150.58, 150.48, 149.42, 148.90, 138.12, 137.75, 135.81, 135.77, 135.63, 135.37, 134.76, 134.13, 124.94, 124.12, 123.60, 123.54, 121.85, 120.97, 120.82, 120.79, 119.71, 119.24, 116.91, 114.48, 114.15, 110.79, 31.62, 22.69, 18.58 (Ar-CH_3_), 14.16. ESI-MS (m/z): Calcd for C_41_H_28_O_5_N_3_Ir: 835.1658; Found: 836.1729 [M + H]^+^. Elem anal. Calcd for C_41_H_28_O_5_N_3_Ir: C, 58.98; H, 3.38; N, 5.03; O, 9.58%. Found: C, 59.19; H, 3.58; N, 4.92; O, 9.50%.

[(ppy-CHO)_2_Ir(L2)] (Ir5) Yield: 39 mg (87%). Selected FT-IR (KBr): 3060 (C-H), 1750 (C=O), 1,600 (C=N), 1,450 (C-N), 1,280 (C-F), 1,150 (C-O). ^1^H NMR (500 MHz, CDCl_3_): *δ* 9.59 (d, *J* = 4.0 Hz, 1H, HC = O), 9.58 (s, 1H, HC = O), 8.93 (d, *J* = 5.3 Hz, 1H), 8.86 (d, *J* = 5.5 Hz, 1H), 8.16–8.12 (m, 1H, HC = N), 8.04 (d, *J* = 8.0 Hz, 1H), 7.88 (t, *J* = 7.7 Hz, 2H), 7.72 (dd, *J* = 11.4, 8.0 Hz, 2H), 7.38–7.35 (m, 1H), 7.35–7.32 (m, 1H), 7.32–7.27 (m, 2H), 7.21 (d, *J* = 7.1 Hz, 1H), 7.17–7.14 (m, 2H), 7.04–7.00 (m, 1H), 6.99 (dd, *J* = 7.9, 1.4 Hz, 1H), 6.69 (d, *J* = 8.6 Hz, 1H), 6.63 (d, *J* = 5.7 Hz, 2H), 6.49–6.44 (m, 2H), 6.21 (dd, *J* = 7.6, 0.4 Hz, 1H). ^13^C NMR (126 MHz, CDCl_3_): *δ* 193.24 (HC = O), 193.00 (HC = O), 167.20, 166.91, 166.68, 161.75, 160.50, 154.08, 152.90, 151.83, 150.90, 150.66, 150.56, 149.50, 148.98, 138.20, 137.83, 135.89, 135.85 135.71, 135.45, 134.84, 134.21, 125.02, 124.20, 123.68, 123.62, 123.50, 121.93, 121.05, 120.90, 120.87, 119.79, 119.32, 116.99, 114.56, 114.23, 110.87, 31.70, 22.77, 14.24. ^19^F NMR (471 MHz, CDCl_3_): *δ* -64.92 (s). ESI-MS (m/z): Calcd for C_41_H_25_O_5_N_3_F_3_Ir: 889.1376; Found: 890.1455 [M + H]^+^. Elem anal. Calcd for C_41_H_25_O_5_N_3_F_3_Ir: C, 55.40; H, 2.84; N, 4.73; O, 9.00%. Found: C, 55.62; H, 3.05; N, 4.62; O, 8.91%.

[(ppy-CHO)_2_Ir(L3)] (Ir6) Yield: 33 mg (81%). Selected FT-IR (KBr): 1720 (C=O), 1,600 (C=N), 1,380 (C-N), 1,140 (C-O). ^1^H NMR (500 MHz, CDCl_3_): *δ* 9.60 (s, 1H, HC = O), 8.98 (d, *J* = 5.5 Hz, 1H), 8.88 (d, *J* = 5.5 Hz, 1H), 8.14–8.11 (m, 1H, HC = N), 8.06–8.00 (m, 1H), 7.89–7.80 (m, 2H), 7.73 (d, *J* = 8.0 Hz, 1H), 7.65 (d, *J* = 8.1 Hz, 1H), 7.37 (dd, *J* = 8.0, 1.5 Hz, 1H), 7.34–7.30 (m, 1H), 7.22 (dd, *J* = 5.1, 3.8 Hz, 1H), 7.18–7.11 (m, 3H), 7.09–7.06 (m, 1H), 7.01–6.97 (m, 1H), 6.74–6.64 (m, 4H), 6.50–6.39 (m, 2H), 6.28–6.25 (m, 1H), 6.24–6.19 (m, 2H). ^13^C NMR (126 MHz, DMSO): *δ* 193.68 (HC = O), 193.55 (HC = O), 166.29, 166.27, 166.14, 163.06, 160.20, 152.20, 152.00, 151.44, 150.84, 150.70, 148.41, 147.62, 144.07, 139.02, 138.76, 136.81, 135.53, 135.50, 133.31, 132.14, 126.55, 125.15, 124.70, 124.63, 124.53, 124.22, 123.85, 122.80, 121.60, 121.55, 120.82, 117.72, 116.60, 115.58, 113.81, 31.43, 22.54, 14.45. ESI-MS (m/z): Calcd for C_40_H_26_O_5_N_3_Ir: 821.1502; Found: 822.1561 [M + H]^+^. Elem anal. Calcd for C_40_H_26_O_5_N_3_Ir: C, 58.53; H, 3.19; N, 5.12; O, 9.75%. Found: C, 58.81; H, 3.38; N, 5.02; O, 9.67%.

## Data Availability

The original contributions presented in the study are included in the article/Supplementary Material, further inquiries can be directed to the corresponding authors.
