# Supplementary material for: Lysosomal Targeted Cyclometallic Iridium(Ⅲ) Salicylaldehyde-Coumarin Schiff Base Complexes and Anticancer Application
Source: Front Chem. 2022 May 10;10:906954. doi: 10.3389/fchem.2022.906954 (PMC9127163; doi:10.3389/fchem.2022.906954)
Supplement: Supplementary file 1 [file DataSheet1.docx]

Supplementary Material

# Supplementary Data

NMR spectra were obtained on a Bruker DPX 500 spectrometers instrument, with the chemical shifts reported in ppm using tetramethylsilane (TMS) as an internal standard. Mass spectrum (MS) was measured on a Waters Q-TOF Micro^TM^ mass spectrometer. Elemental analysis was performed on a VarioMICRO CHNOS elemental analyzer. The quantum chemical data were calculated using a Inspur's enterprise server. UV-vis spectroscopy was performed on a PERSEE TU-1901 UV spectrometer. Fluorescence spectra were collected by a Hitachi F-4600 fluorescence spectrophotometer, with a 400 V voltage and 5 nm slit width for both excitation and emission. Induction of apoptosis, cell cycle and mitochondrial membrane potential (MMP) determination was carried out by an ACEA Novocyte2040R flow cytometry. Viability assay (MTT) was measured using a Perlong DNM-9606 microplate reader at an absorbance of 570 nm. Cell uptake and cellular localization were carried out on a Carl Zeiss AG */LSM/880NLO two photon laser scanning confocal microscope.

**1.1 NMR spectra**

NMR spectra were acquired in 5 mm NMR tubes at 298 K on Bruker DPX 500 (^1^H NMR: 500.13 MHz; ^13^C NMR: 126 MHz; ^19^F NMR: 471 MHz) spectrometers. ^1^H NMR chemical shifts were internally referenced to CHCl_3_ (7.26 ppm) for chloroform-*d*_1_, and 77.41 ppm, 77.16 ppm, 76.91 ppm for ^13^C NMR. All data was carried out using XWIN-NMR version 3.6 (Bruker UK Ltd.).

**1.2 X-ray crystallography**

All diffraction data were obtained on a Bruker Smart Apex CCD diffractometer equipped with graphite-monochro mated Mo Kα radiation. Absorption corrections were applied using SADABS program. SQUEEZE option was used to remove the non-localized electron density at the final step of structure refinement. The structures were solved by direct methods using SHELXS (TREF) with additional light atoms found by Fourier methods. Complexes were refined against *F*2 using SHELXL, and hydrogen atoms were added at calculated positions and refined riding on their parent atoms. X-ray crystallographic data for **Ir1** are available as Table S1 has been deposited in the Cambridge Crystallographic Data Centre under the accession numbers CCDC 2158102, respectively. X-ray crystallographic data in CIF format are available from the Cambridge Crystallographic Data Centre.

**1.3 UV-vis spectroscopy**

UV-vis spectra of these compounds were recorded by TU-1901 UV spectrophotometer with 1 cm path-length quartz cuvettes (3 mL). Spectra were processed using UV Winlab software. Experiments were carried out at 298 K unless otherwise stated.

**1.4 Reaction with NADH**

The reaction of **Ir1-Ir6** (ca. 1 *µ*M) with NADH (ca. 100 *µ*M) in 10% MeOH/90% H_2_O (*v*/*v*) was monitored by UV-vis at 298 K after various time intervals. TONs were calculated from the difference in NADH concentration after 8 h divided by the concentration of iridium catalyst. The concentration of NADH was obtained using the extinction coefficient ε_339_ = 6220 M^-1^ cm^-1^.

**1.5 Cell culture**

A549 (lung cancer cells) and BEAS-2B (human normal lung epithelial cells) were obtained from Shanghai Institute of Biochemistry and Cell Biology (SIBCB) and were grown in Dubelco's Modified Eagle Medium (DMEM). All media were supplemented with 10% fetal bovine serum, and 1% penicillin-streptomycin solution. All cells were grown at 310 K in a humidified incubator under 5% CO_2_ atmosphere.

**1.6 MTT assay**

After plating 5000 cells per well in 96-well plates, the cells were preincubated in drug-free media at 310 K for 24 h before adding different concentrations of drugs to be tested. To prepare the stock solution of drugs, the solid compound was dissolved in DMSO. This stock was further diluted using cell culture medium until working concentrations were achieved. The drug exposure period was 24 h. Subsequently, 15 *µ*L of 5 mg mL^-1^ MTT solution was added to form a purple formazan. Afterwards, 100 *μ*L of DMSO was transferred into each well to dissolve the purple formazan, and results were measured using a microplate reader (DNM-9606, Perlong Medical, Beijing, China) at an absorbance of 570 nm. Each well was triplicated and each experiment repeated at least three times. IC_50_ values quoted are mean±SEM.

**1.7 Intracellular localization assay**

The slides inoculated with A549 cells were placed in a CO_2_ (5%) incubator at a constant temperature (310 K) for 24 h, and then **Ir2** and **Ir5** (10 *μ*M) were added and cultured for another 2 h under the same conditions. Mito Tracker Deep Red (MTDR, used to calibrate mitochondria) and Lyso Tracker Deep Red (LTDR, used to calibrate lysosomes) were incubated with pretreated cells for 30 min, respectively. After that, MTDR and LTDR staining solution were absorbed, and cold PBS was added for rinsing, and then about 2 mL PBS was added slowly. Cell localization imaging by laser confocal microscopy was observed under dark conditions. **Ir2** and **Ir5** were excited at 405 nm and collected at 430-580 nm. LTDR was excited at 594 nm and collected at 630±30 nm. MTDR was excited at 644 nm and collected at 690±30 nm.

**1.8 Lysosomal damage assay**

A549 cells seeded into six-well plate (Corning) were exposed to **Ir2** and **Ir5** at the indicated concentrations for 6 h. The cells were then washed twice with PBS and incubated with AO (5 *μ*M) at 310 K for 15 min. The cells were washed twice with PBS and visualized by confocal microscopy (LSM/880NLO). Emission was collected at 510±20 nm (green) and 625±20 nm (red) upon excitation at 488 nm.

**1.9 Cellular uptake mechanism assay**

The slides inoculated with A549 cells were placed in a CO_2_ (5%) constant temperature (310 K) incubator for pre-cultivation for 24 h, and then cultured under the following three different conditions: 277 K, 1 h; Culture at 310 K in chloroquine concentration of 50 *μ*M for 1 h. At 310 K, the cells were cultured in CCCP with a concentration of 10 *μ*M for 1 h. The medium was taken out, and **Ir2** and **Ir5** with a concentration of 10 *μ*M were added, respectively, and incubated for 15 min. After rinsing with PBS, the absorption of the complexes was observed by laser confocal microscope. Emission was collected at 430-490 nm after excitation at 405 nm.

**1.10 Induction of apoptosis**

Flow cytometry analysis of apoptotic populations of the cells caused by exposure to **Ir2** were carried out using the Annexin V-FITC Apoptosis Detection Kit (Beyotime Institute of Biotechnology, China) according to the supplier’s instructions. Briefly, A549 and BEAS-2B cells (1.5×10^6^/2 mL per well) were seeded in a six-well plate. Cells were preincubated in drug-free media at 310 K for 24 h, after which **Ir2** were added at concentrations of 0.5×IC_50_, 1.0×IC_50_ and 2.0×IC_50_ of **Ir2** against cells. After 24 h of exposure, cells were collected, washed once with PBS, and suspended in 195 *μ*L of Annexin V-FITC binding buffer, which was then added to 5 *μ*L of Annexin V-FITC and 10 *μ*L of PI, then incubated at room temperature in the dark for 15 min. Subsequently, the buffer placed in an ice bath in the dark. The samples were analyzed by a flow cytometer (ACEA NovoCyte, Hangzhou, China).

**1.11 Cell cycle analysis**

A549 and BEAS-2B cells at 1.5×10^6^ per well were seeded in a six-well plate. Cells were preincubated in drug-free media at 310 K for 24 h, then **Ir2** were added at concentrations of 0.5×IC_50_, 1.0×IC_50_, 2.0×IC_50_ and 3.0×IC_50_. After 24 h of exposure, supernatants were removed by suction and cells were washed with PBS. Finally, cells were harvested using trypsin-EDTA and fixed for 24 h using cold 70% ethanol. DNA staining was achieved by suspending the cell pellets in PBS containing propidium iodide (PI) and RNAse. Cell pellets were washed and suspended in PBS solution before being analyzed in a flow cytometer (ACEA NovoCyte, Hangzhou, China) using excitation of DNA-bound PI at 488 nm, with emission at 585 nm. Data were processed using NovoExpress™ software. The cell cycle distribution was shown as the percentage of cells containing G1, S and G2 DNA as identified by propidium iodide staining.

**1.12 Mitochondrial membrane assay**

Analysis of the changes of mitochondrial potential in cells after exposure to **Ir2** were carried out using the mitochondrial membrane potential assay kit with JC-1 (Beyotime Institute of Biotechnology, Shanghai, China) according to the manufacturer’s instructions. Briefly, 1.5×10^6^ A549 and BEAS-2B cells were seeded in six-well plates left to incubate for 24 h in drug-free medium at 310 K in a humidified atmosphere. Drug solutions, with the concentration changed from 0.5×IC_50_ to 2.0×IC_50_ of **Ir2** against cells, were added in triplicate, and the cells were left to incubate for a further 24 h under similar conditions. Supernatants were removed by suction, and each well was washed with PBS before detaching the cells using trypsin-EDTA. Staining of the samples was done in flow cytometry tubes protected from light, incubating for 30 min at ambient temperature. The samples were immediately analyzed by a flow cytometer (ACEA NovoCyte, Hangzhou, China). For positive controls, the cells were exposed to carbonyl cyanide 3-chlorophenylhydrazone, CCCP (5 *μ*M), for 20 min. Data were processed using NovoExpress™ software.

**1.13 ROS determination**

Flow cytometry analysis of ROS generation in A549 and BEAS-2B cells caused by exposure to **Ir2** was carried out using the Reactive Oxygen Species Assay Kit (Beyotime Institute of Biotechnology, Shanghai, China) according to the supplier’s instructions. Briefly, 1.5×10^6^ cells per well were seeded in a six-well plate. Cells were preincubated in drug-free media at 310 K for 24 h in a 5% CO_2_ humidified atmosphere, and then drugs were added at concentrations of 1.0×IC_50_ and 2.0×IC_50_. After 24 h of exposure, cells were washed twice with PBS and then incubated with the DCFH-DA probe (10 *μ*M) at 310 K for 30 min, and then washed triple immediately with PBS. The fluorescence intensity was analyzed by flow cytometry (ACEA NovoCyte, Hangzhou, China). Data were processed using NovoExpress™ software.

**1.14 Synthesis of Schiff base pro-ligands (L1-L3)**

Synthesis of **L1**: 0.12 g (1.0 mmol) of salicylic aldehyde and 0.18 g (1.0 mmol) of 7-amino-4-methylcoumarin were placed in a 200 mL round-bottom flask, respectively. Anhydrous methanol (40 mL) was added to dissolve them, and 1 drop of formic acid was added to catalyse them. Reflux stirring was performed at 353 K for 12 h until 7-amino-4-methylcoumarin was completely consumed (TLC was used to monitor the reaction). After most of the solvents were removed by rotary evaporation apparatus, they were placed at 257 K for 12 h, filtered, and washed with cold methanol and ether for several times to obtain orange-red ligand **L1**. Yield: 0.23 g (81%). ^1^H NMR (500 MHz, CDCl_3_): δ 8.65 (s, 1H), 7.64 (d, *J* =8.3 Hz, 1H), 7.43 (t, *J* = 7.8 Hz, 2H), 7.23-7.19 (m, 2H), 7.09-6.95 (m, 3H), 6.28 (d, *J* = 0.9 Hz, 1H), 2.46 (d, *J* = 1.0 Hz, 3H).^13^C NMR (126 MHz, CDCl_3_): δ 164.51,161.29, 160.67, 154.46, 152.02, 151.60, 134.11, 132.88, 125.64, 119.44, 118.86,118.55, 118.26, 117.43, 114.52, 108.78, 18.72. ESI-MS (*m/z*): Calcd for C_17_H_12_O_3_N, 278.1; Found: 278.1 [M-H]^–^.

**L2** was synthesized from 7-amino-4-trifluoromethyl coumarin (0.23 g, 1.0 mmol) and salicylaldehyde (0.12 g, 1.0 mmol) in the same way as **L1**. Yield: 0.28 g (84%).^1^H NMR (500 MHz, CDCl_3_): δ 8.66 (s, 1H), 7.78 (dd, *J*= 9.0, 1.6 Hz, 1H), 7.58-7.48 (m, 1H), 7.46 (dd, *J* = 10.9, 4.6 Hz, 2H), 7.06 (d, *J* = 8.6Hz, 1H), 7.05-6.96 (m, 2H), 6.78 (s, 1H), 6.65-6.54 (m, 1H).^13^C NMR (126 MHz, CDCl_3_): δ 165.35, 161.38, 158.77, 155.37, 152.87, 151.22, 134.56, 133.07, 126.43,119.59, 119.04, 118.72, 117.56, 115.12, 115.08, 111.92, 109.51. ESI-MS (*m/z*): Calcd for C_17_H_9_O_3_NF_3_, 332.1; Found: 332.1 [M-H]^-^.

**L3** was synthesized by 6-aminocoumarin (0.16 g, 1.0 mmol) and salicylaldehyde (0.12 g, 1.0 mmol) using the same method as **L1** and **L2**. Yield: 0.23 g (86%). ^1^H NMR (500 MHz, DMSO): δ 12.83 (s, 1H), 9.02 (s, 1H), 8.09 (d, *J* = 9.6 Hz, 1H), 7.80 (d, *J* = 2.5 Hz, 1H), 7.72 (dd, *J* = 8.8, 2.6 Hz, 1H), 7.68 (dd, *J* = 7.7, 1.6 Hz, 1H), 7.50 (d, *J* = 8.8 Hz, 1H), 7.44 (ddd, *J* = 8.3, 7.4, 1.7 Hz, 1H), 7.00 (ddd, *J* = 11.3, 6.1, 2.4 Hz, 2H), 6.57 (d, *J* = 9.5 Hz, 1H). ^13^C NMR (126 MHz, DMSO): δ 164.24, 160.64, 160.29, 152.65, 144.96, 144.44, 133.96, 132.92, 125.51, 121.24, 119.81, 119.78, 119.75, 117.86, 117.37, 117.12. ESI-MS (*m/z*): Calcd for C_16_H_10_O_3_N: 264.1; Found: 264.1 [M-H]^–^.

**1.15 Synthesis of** **[(ppy)_2_IrCl_2_]_2_ (Dimer 1) and [(ppy-CHO)_2_IrCl_2_]_2_ (Dimer 2)**

IrCl_3_·3H_2_O (0.50 g, 1.7 mmol) into a 100 mL Shirek bottle, seal, circulate vacuum and fill with nitrogen 3 times, the last time until the pressure in the bottle is positive, inject 2-phenylpyridine (0.54 g, 3.5 mmol) with a long needle, then inject 30 mL of a mixture of ethylene glycol monoethyl ether and deionized water (3:1, *v/v*), heat and reflux at 110 ℃ for 24 h, and then filter. The filter cake was washed three times with ethanol and acetone to obtain a yellow solid powder. Dissolve it with a small amount of dichloromethane. Add ether to diffuse and purify to obtain a yellow powder ([(ppy)_2_IrCl]_2_, Dimer 1). Yield: 1.24 g (68%). ^1^H NMR (500 MHz, DMSO) δ 9.81 (d, *J* = 5.7 Hz, 2H), 9.54 (d, *J* = 5.6 Hz, 2H), 8.26 (d, *J* = 8.2 Hz, 2H), 8.18 (d, *J* = 8.0 Hz, 2H), 8.09 (t, *J* = 7.9 Hz, 2H), 8.01 (t, *J* = 7.7 Hz, 2H), 7.76 (dd, *J* = 27.3, 7.6 Hz, 4H), 7.57(t, *J* = 6.6 Hz, 2H),7.45 (t, *J* = 6.6 Hz, 2H),6.90 (t, *J* = 7.4 Hz, 2H),6.90 (t, *J* = 7.4 Hz, 2H), 6.84 (t, *J* = 7.4 Hz, 2H), 6.76 (t, *J* = 7.3 Hz, 2H), 6.69 (t, *J* = 7.4 Hz, 2H), 6.25 (d, *J* = 7.6 Hz, 2H), 5.66 (d, *J* = 7.6 Hz, 2H).

Dimer 2 was synthesized using 4-(2-pyridine)-benzaldehyde (0.64 g, 3.5 mmol) and IrCl_3_·3H_2_O (0.50 g, 1.7 mmol) according to the method of [(ppy)_2_IrCl]_2_. Yield: 1.15 g (57%). 1H NMR (500 MHz, DMSO) δ 9.88 (d, *J* = 5.7 Hz, 1H), 9.66 (s, 1H), 9.63 – 9.56 (m, 2H), 8.47 (d, *J* = 8.1 Hz, 1H), 8.39 (d, *J* = 8.0 Hz, 1H), 8.30 – 8.21 (m, 1H), 8.19 – 8.12 (m, 1H), 8.06 (d, *J* = 8.0 Hz, 1H), 8.02 (d, *J* = 8.0 Hz, 1H), 7.77 – 7.71 (m, 1H), 7.67 – 7.61 (m, 1H), 7.44 (dd, *J* = 8.0, 1.4 Hz, 1H), 7.40 (dd, *J* = 7.9, 1.4 Hz, 1H), 6.75 (d, *J* = 1.3 Hz, 1H), 6.14 (d, *J* = 1.3 Hz, 1H).

**1.16 Synthesis of the [(ppy)_2_Ir(O^N)Cl] (Ir7)**

To a 200 mL round bottom flask, 0.50 g (4.09 mmol) of salicylaldehyde and 0.38 g (4.09 mmol) of aniline were added, respectively. To the above flask, 40 mL of anhydrous methanol was added and added dropwise, 1 d formic acid as a catalyst. The mixed system was stirred and refluxed at 70 ℃ for about 36 h until the reaction was complete. Then the above products (0.020 g, 0.10 mmol) reacted with [(ppy)_2_IrCl]_2_ (0.054 g, 0.05 mmol) and sodium acetate (0.033 g, 0.40 mmol) in nitrogen, and finally the control compound was obtained, Scheme S1. The ^1^H NMR and ESI-MS are shown in Figures S15-S16. Yield: 0.046 g (86%). ^1^H NMR (500 MHz, CDCl_3_): δ 8.02 (s, 1H), 7.68 (d, *J* = 7.9 Hz, 2H), 7.36 (dt, *J* = 20.3, 7.7 Hz, 4H), 7.11 (d, *J* = 7.8 Hz, 1H), 6.97 (d, *J* = 8.6 Hz, 1H), 6.45 (t, *J* = 7.3 Hz, 1H), 1.32 (s, 15H). ESI-MS (*m/z*): Calcd for C_23_H_25_ONIr: 523.6; Found: 524.2 [M-Cl]^+^.


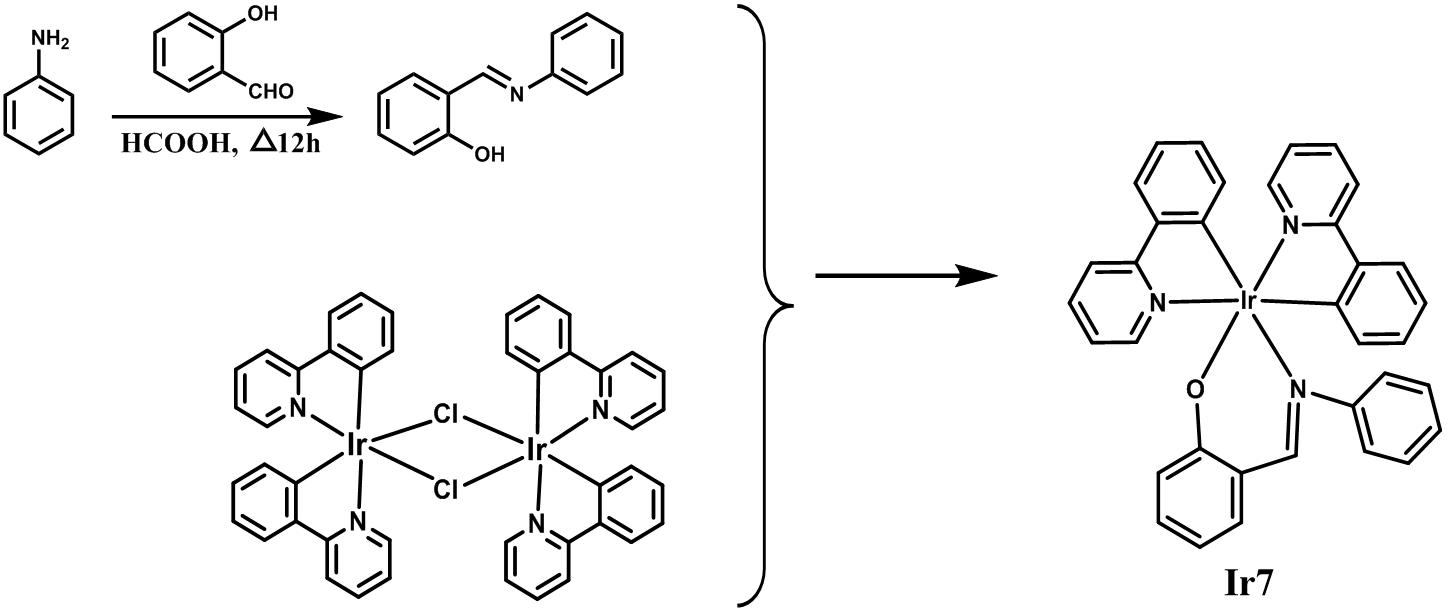


**Supplementary Scheme S1.** Synthesis process of **Ir7**.

# Supplementary Figures and Tables

**
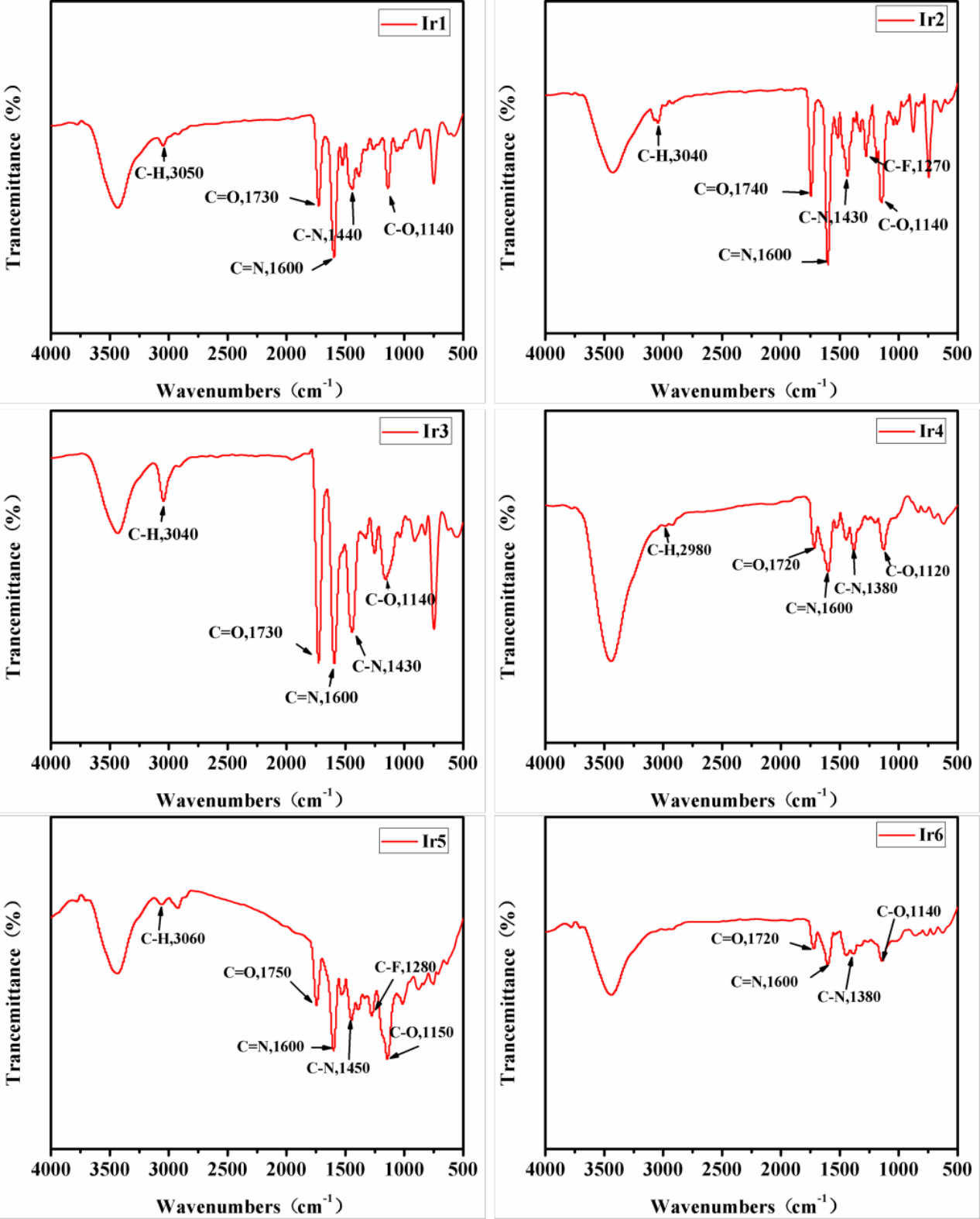
**

**Supplementary Figure S1.** FT-IR spectra of **Ir1-Ir6**.


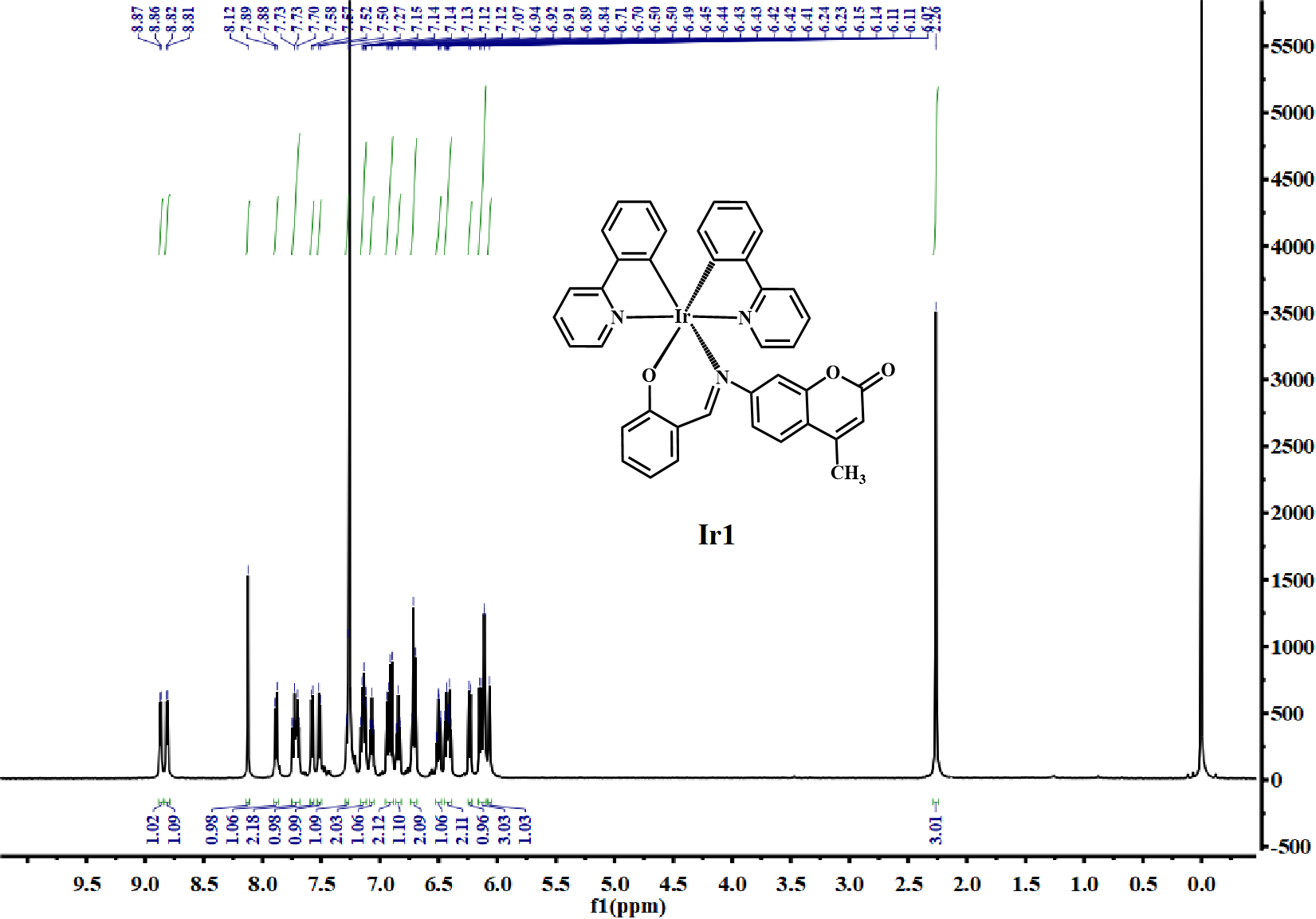

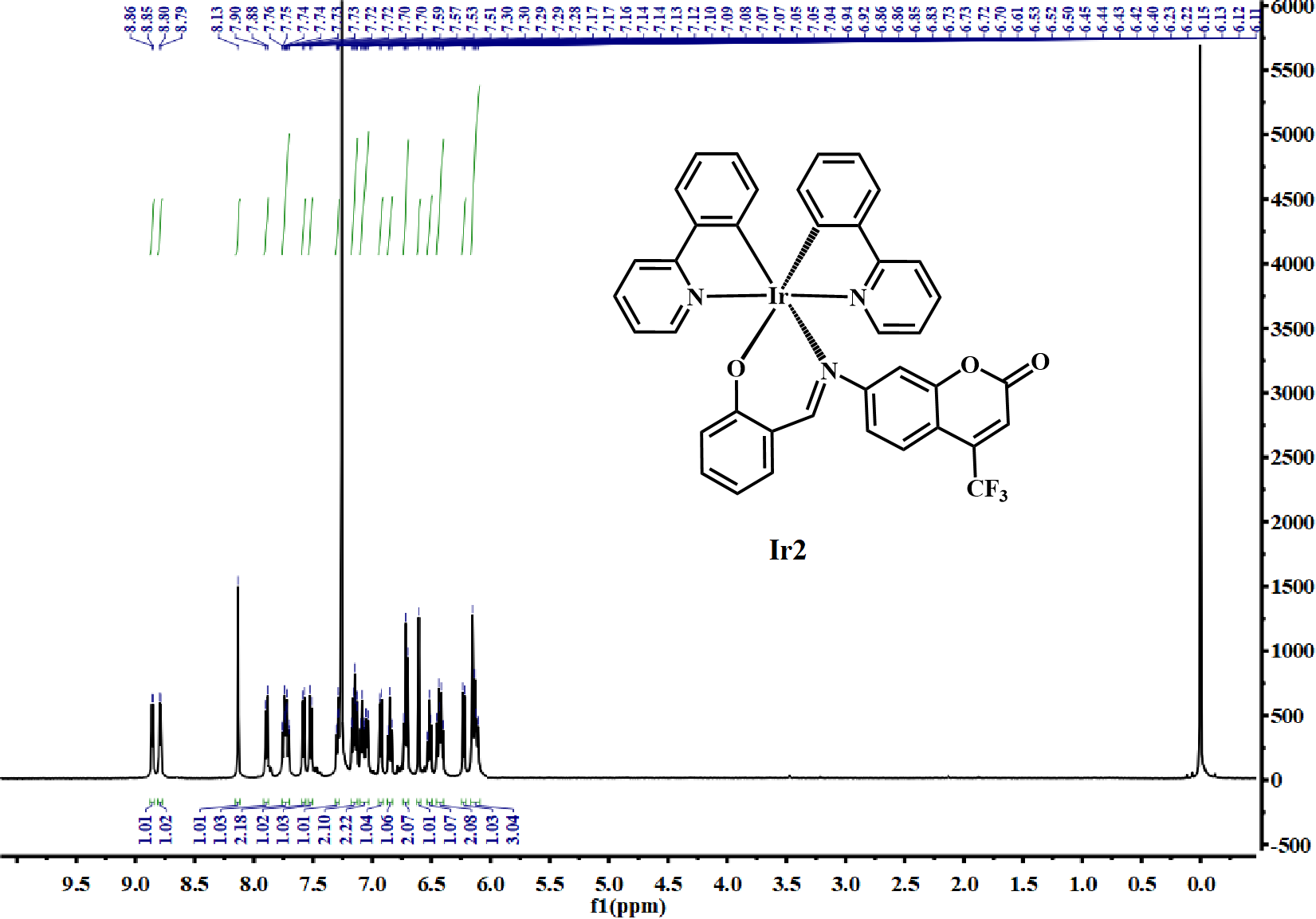

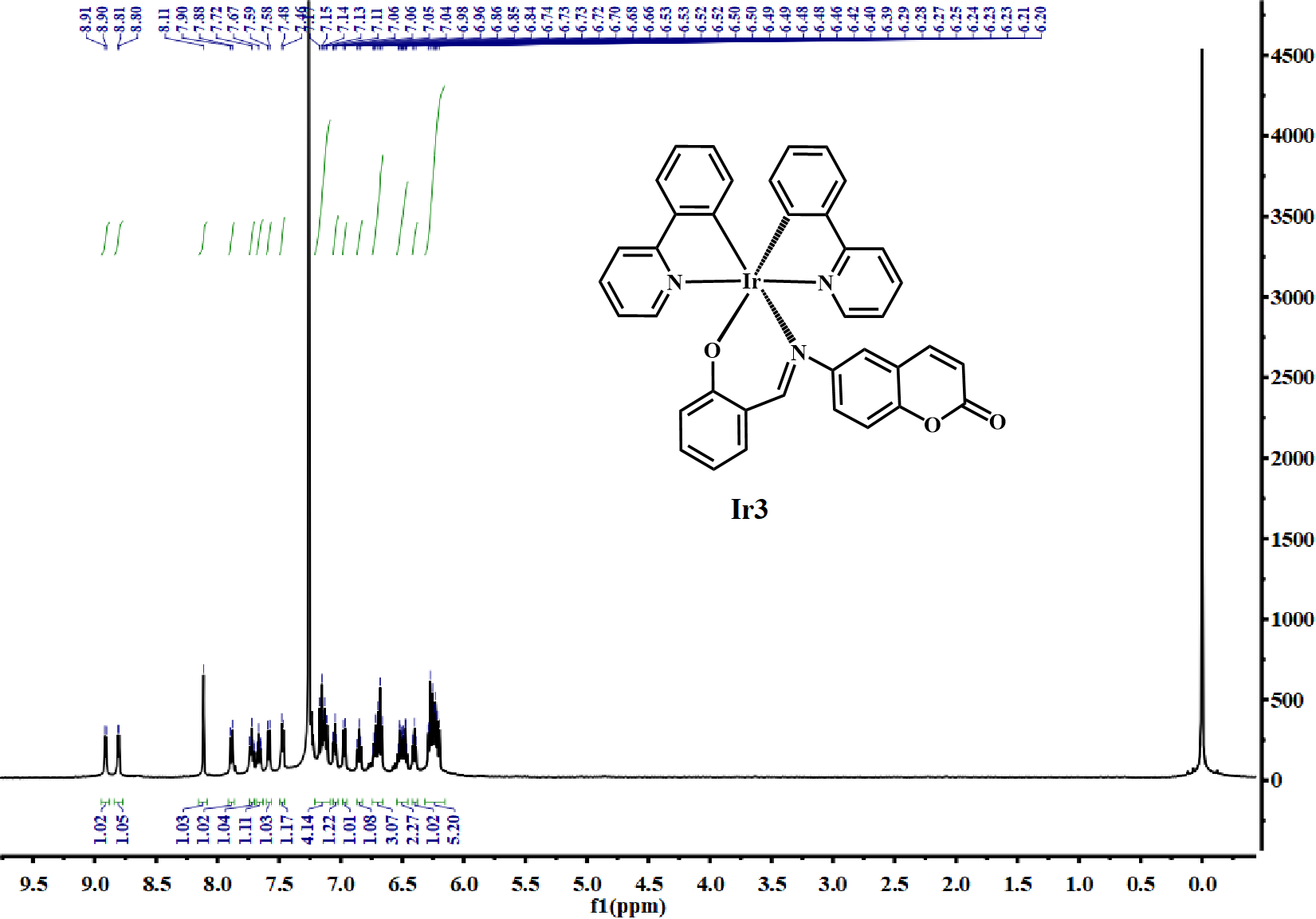

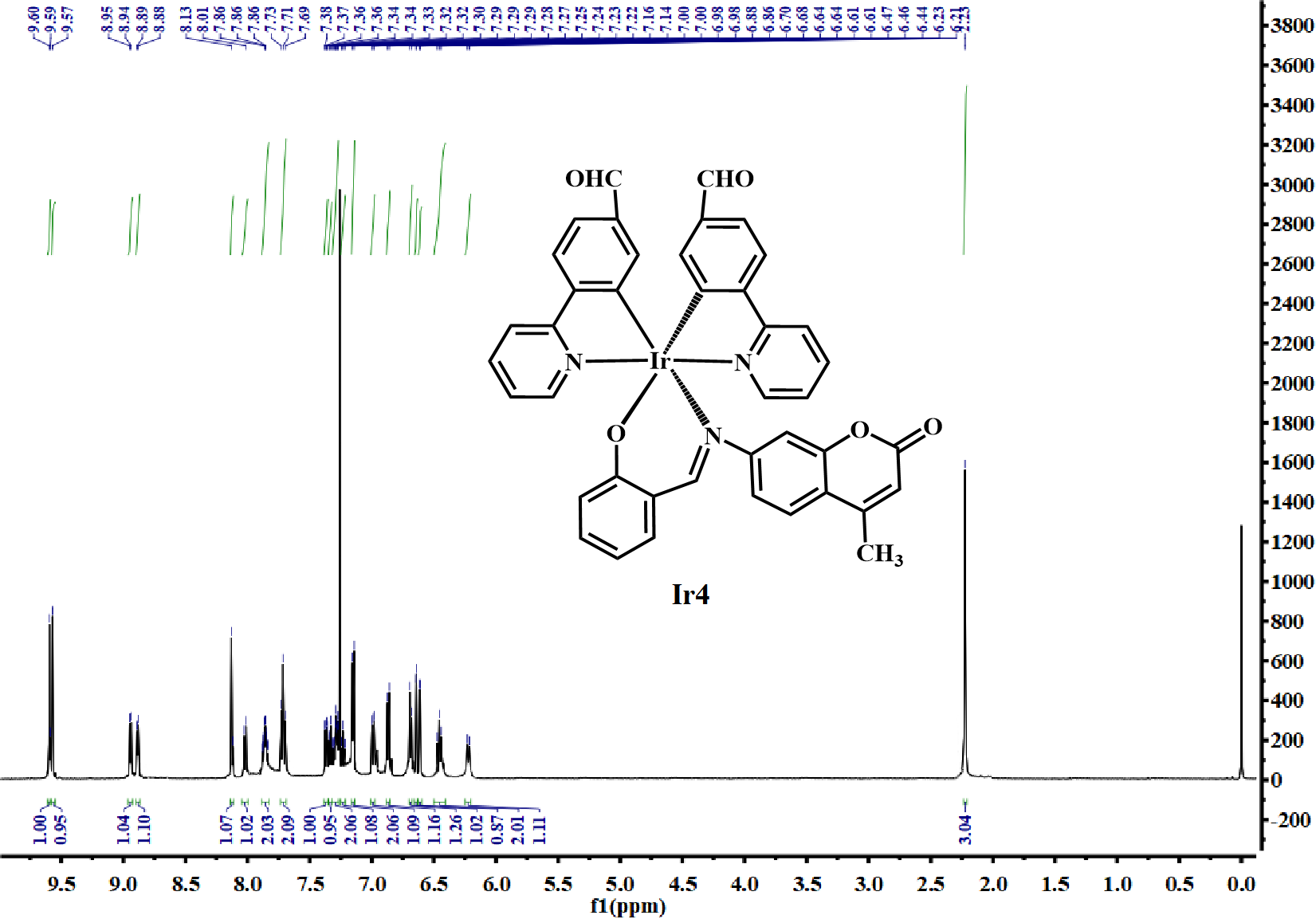

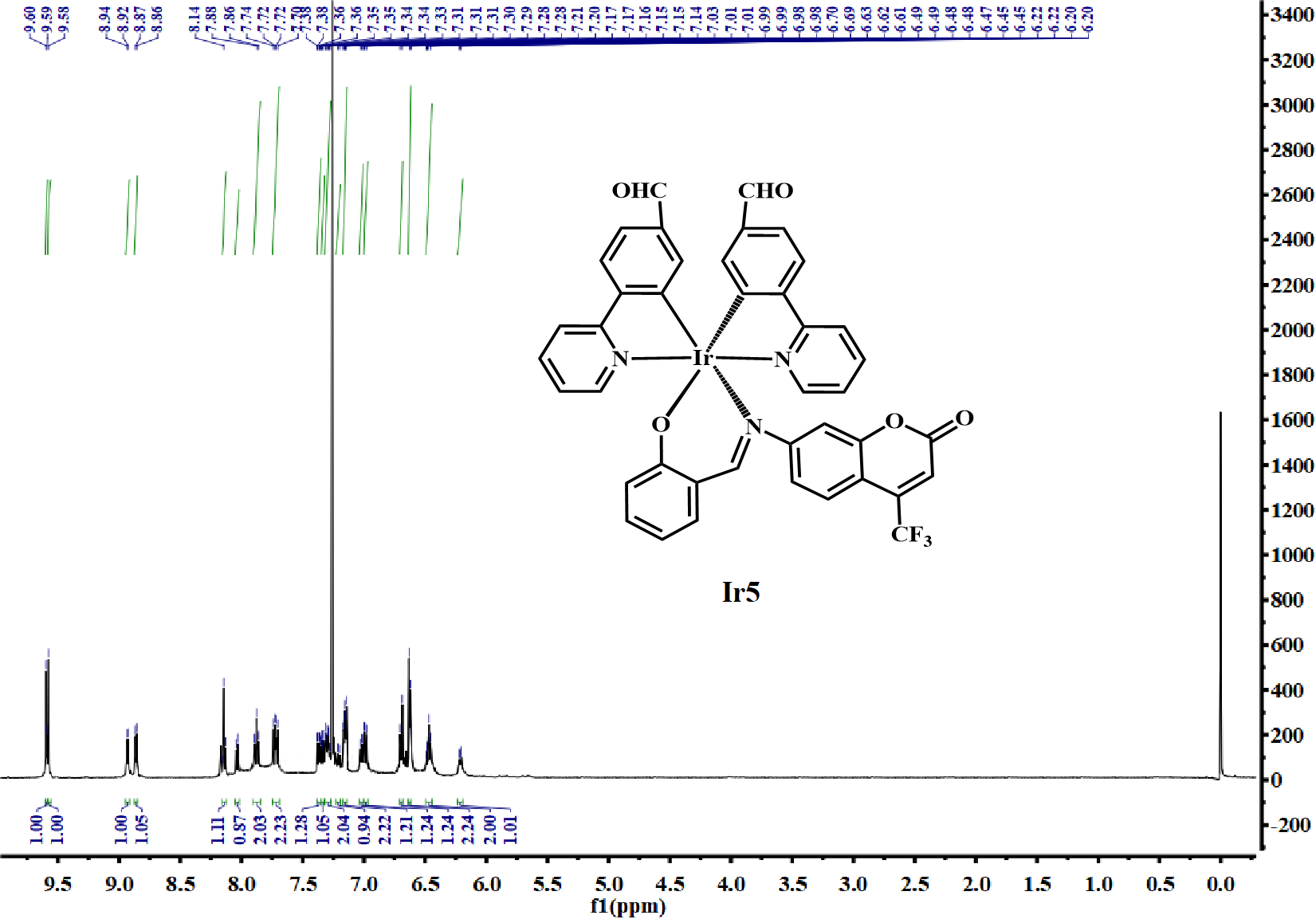

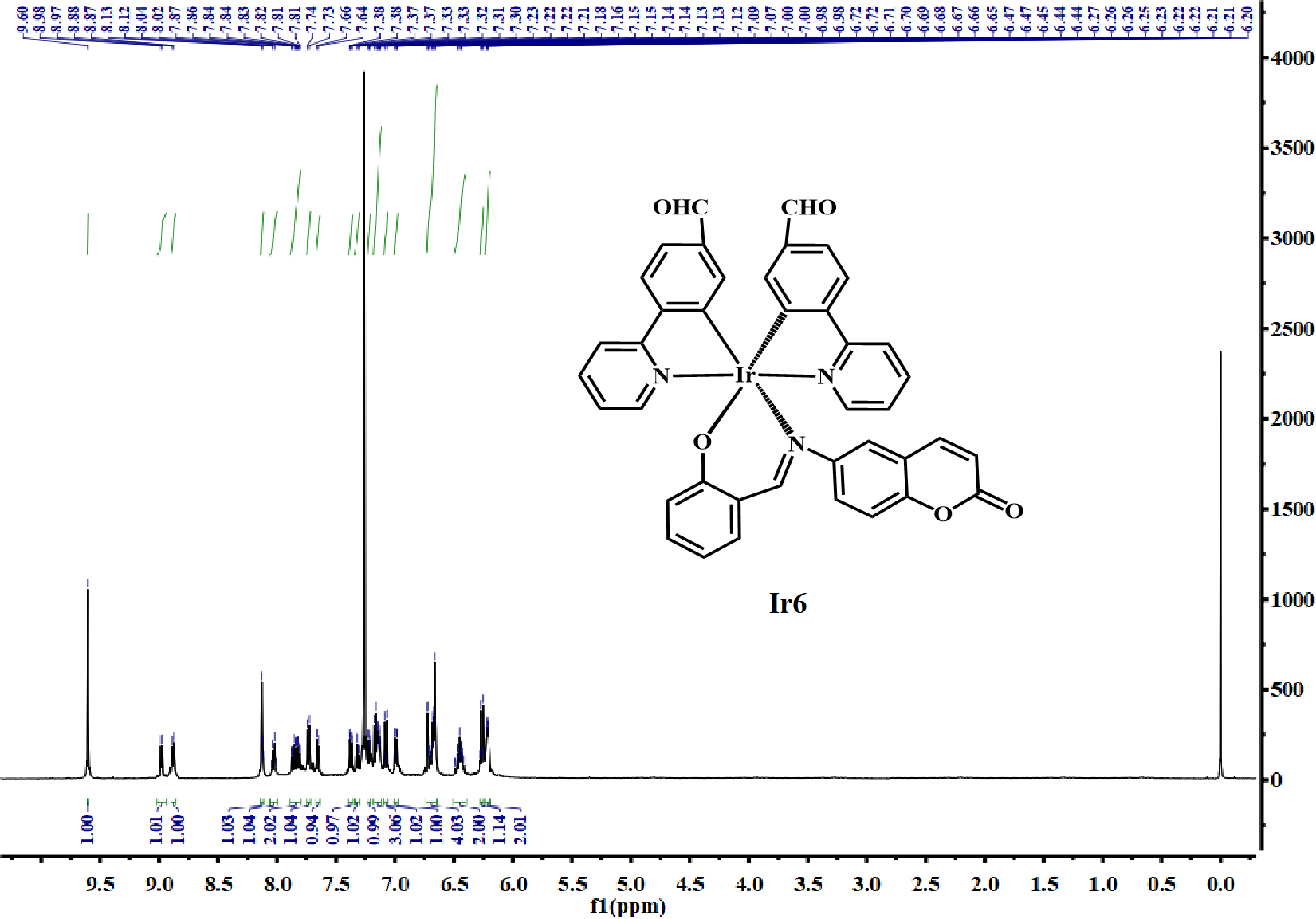
 **Supplementary Figure S2.** ^1^H NMR spectra (500 MHz) of **Ir1-Ir6**.


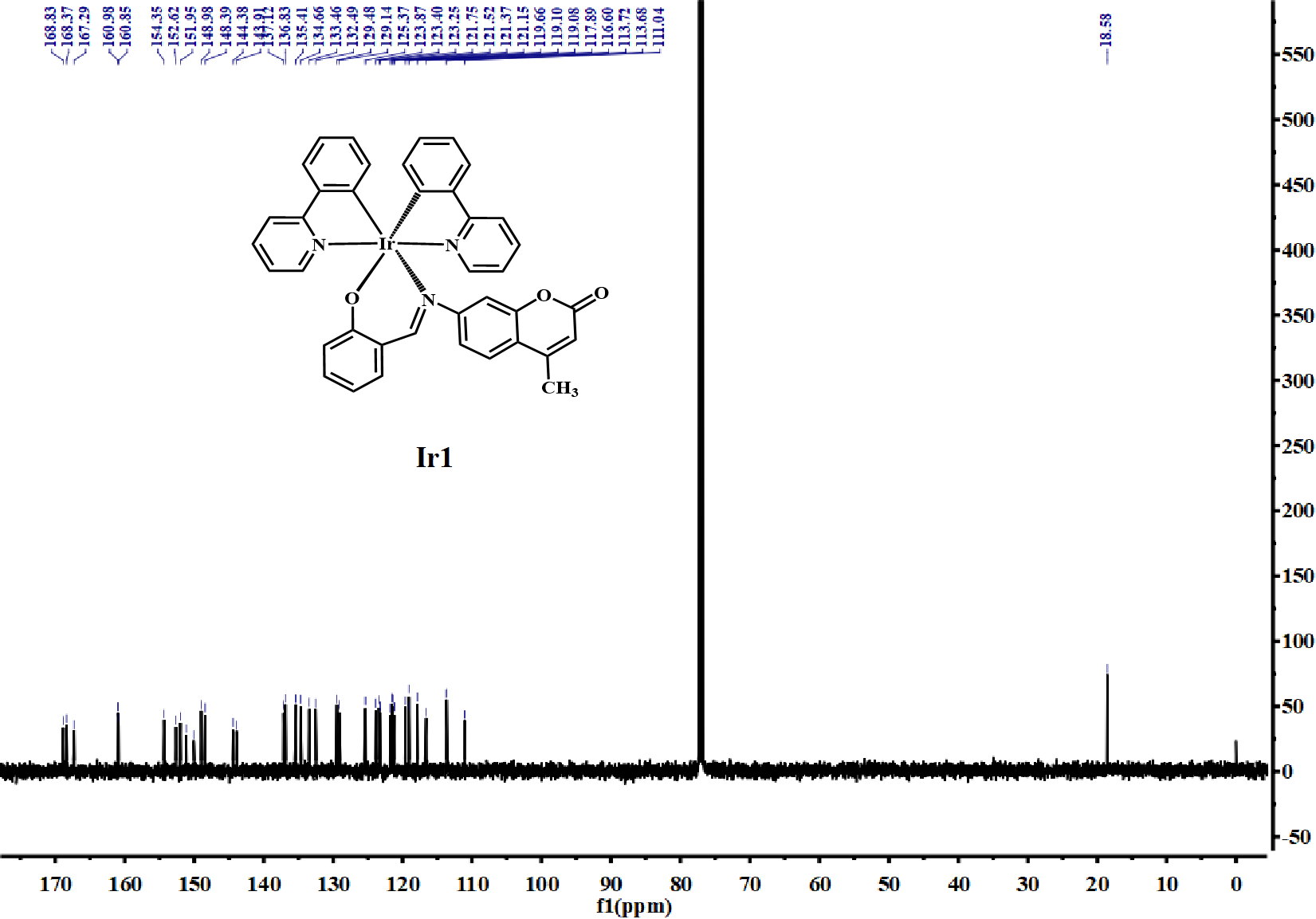

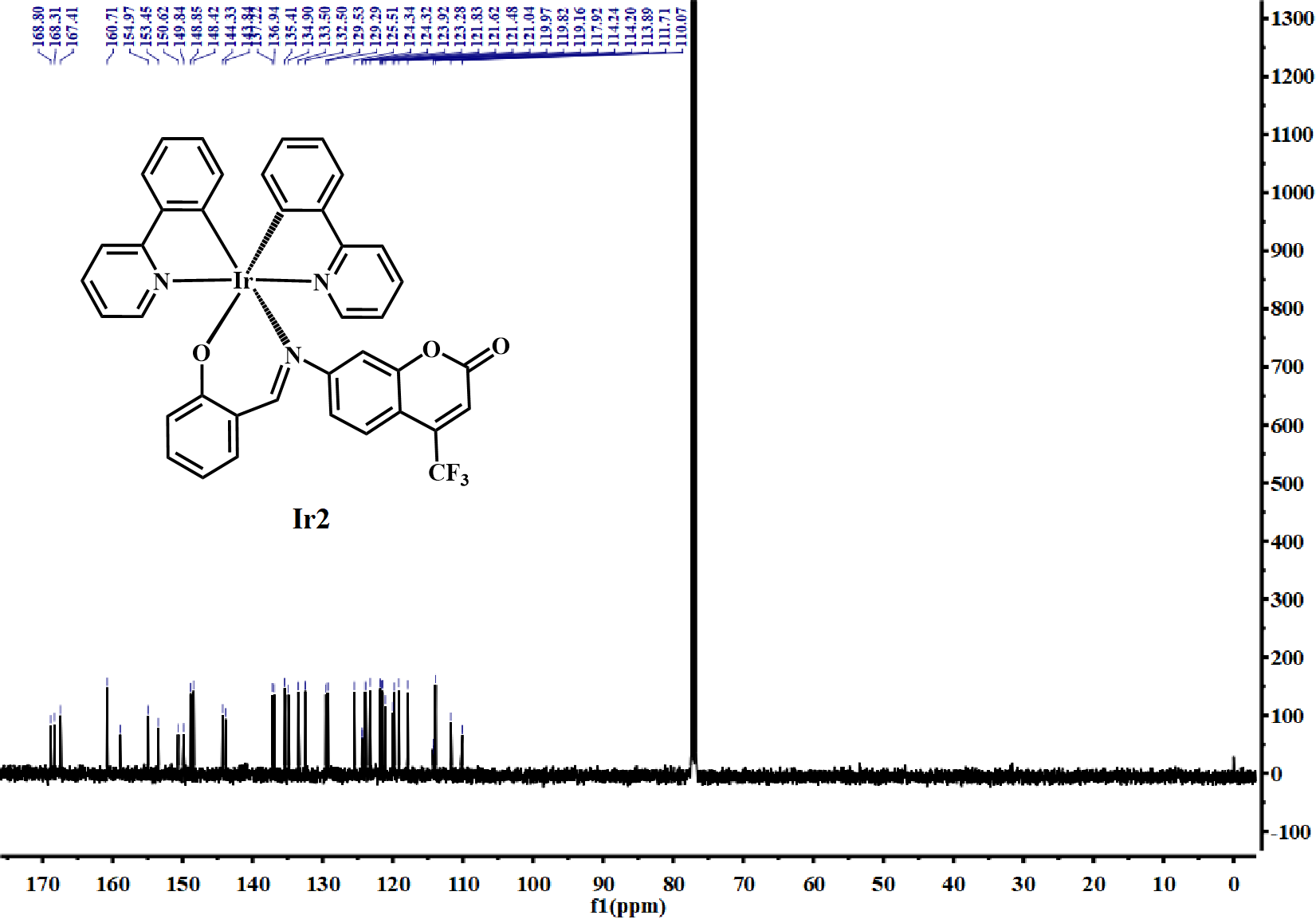

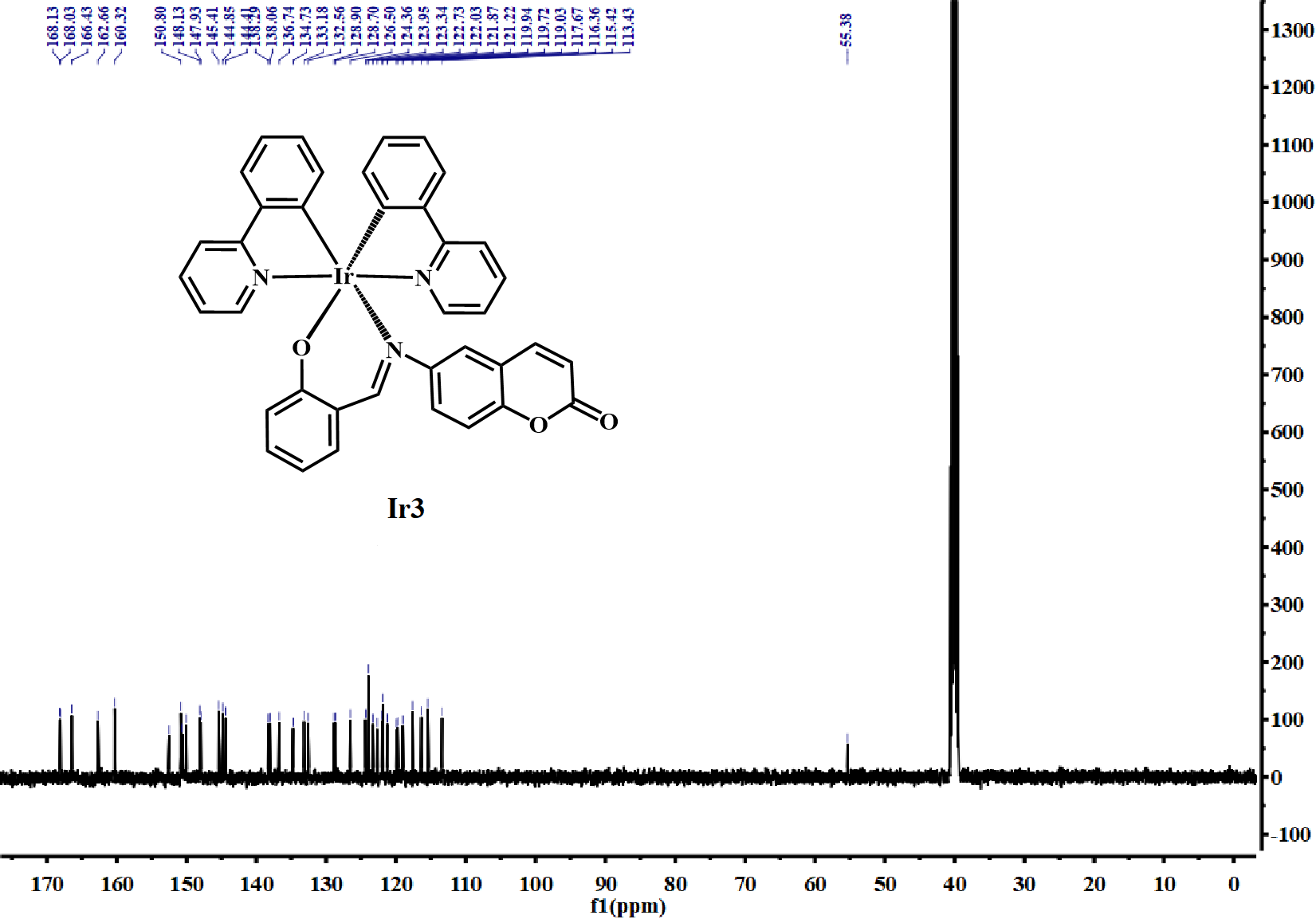

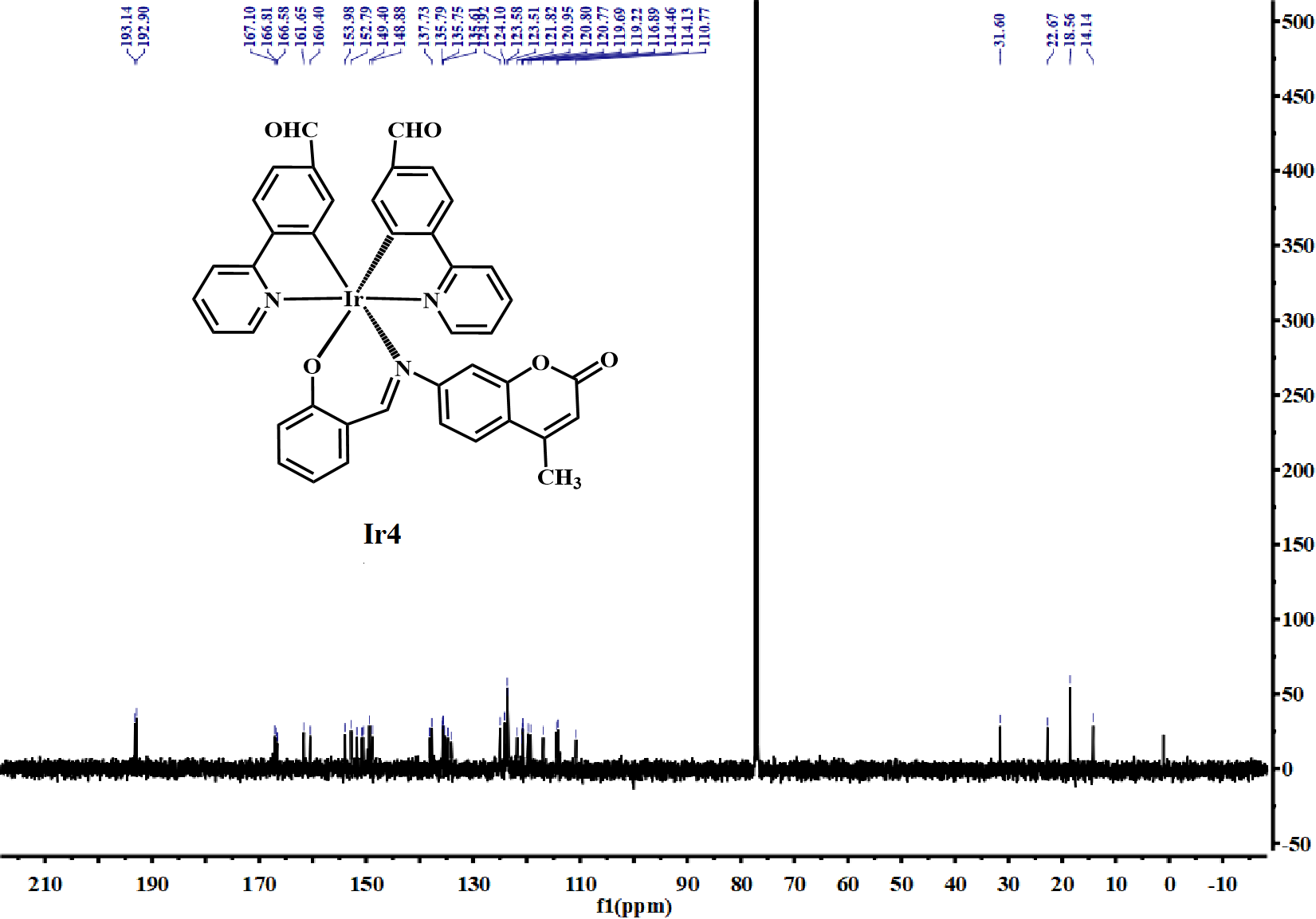

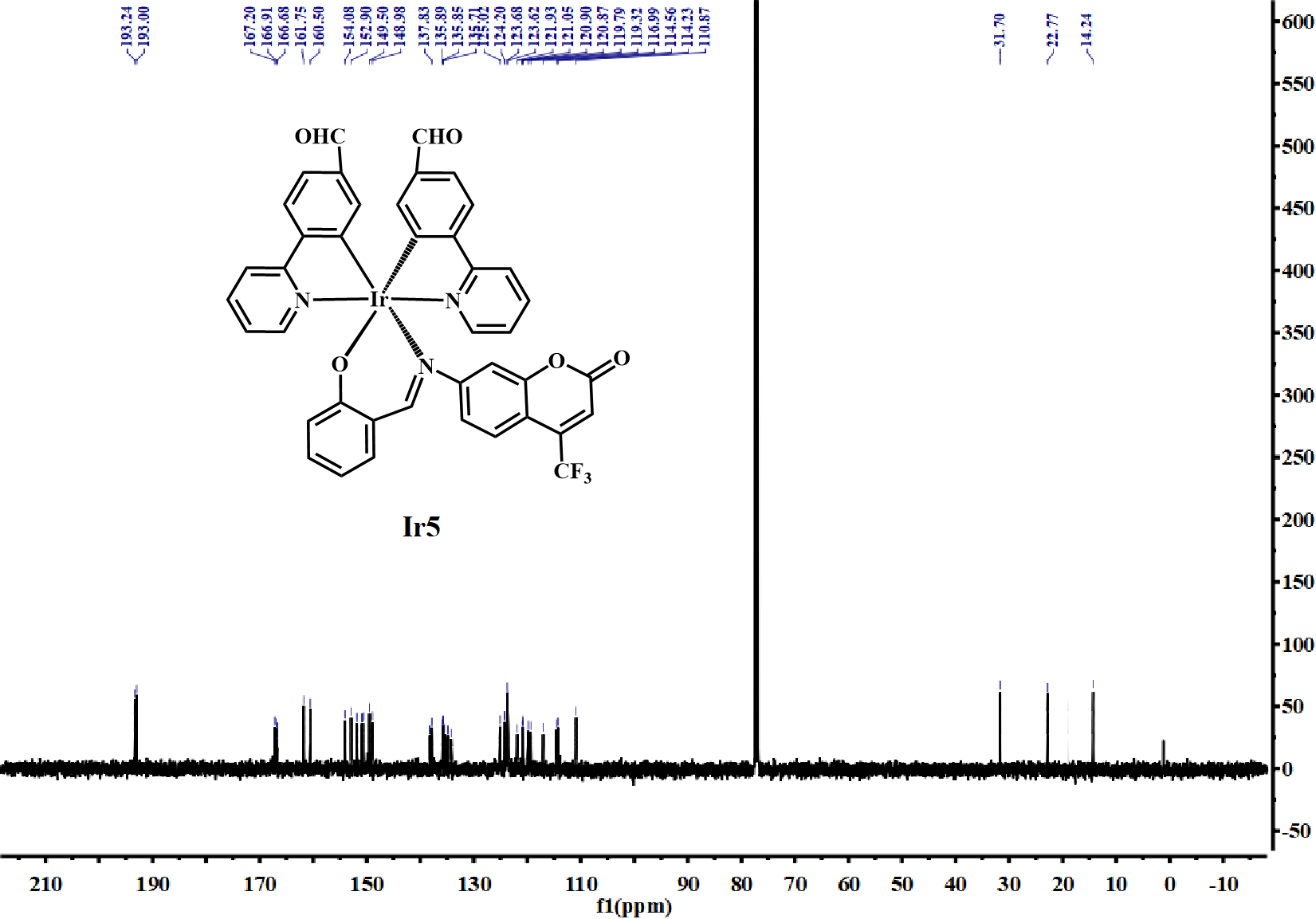

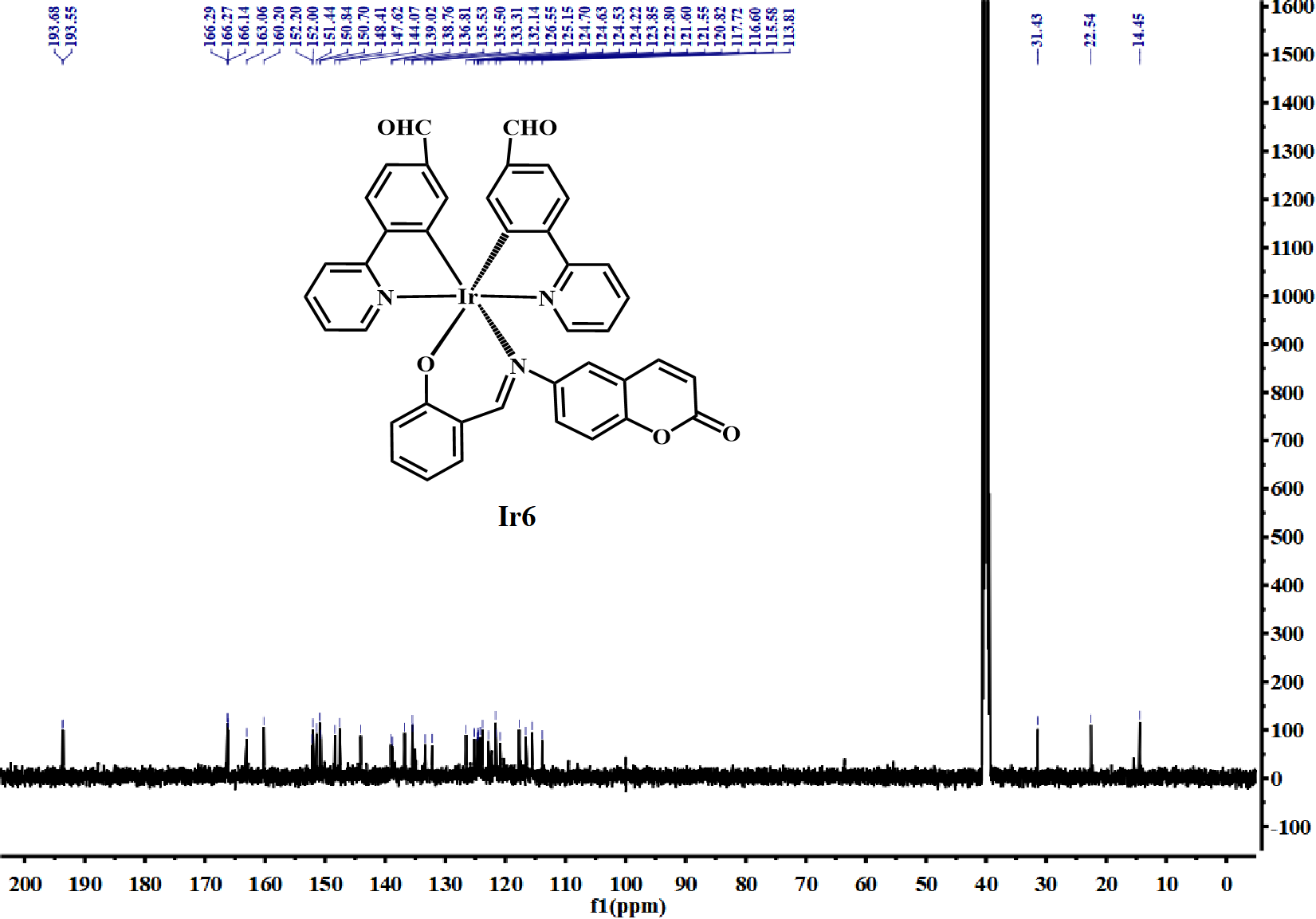
 **Supplementary Figure S3.** ^13^C NMR spectra (126 MHz) of **Ir1-Ir6**.


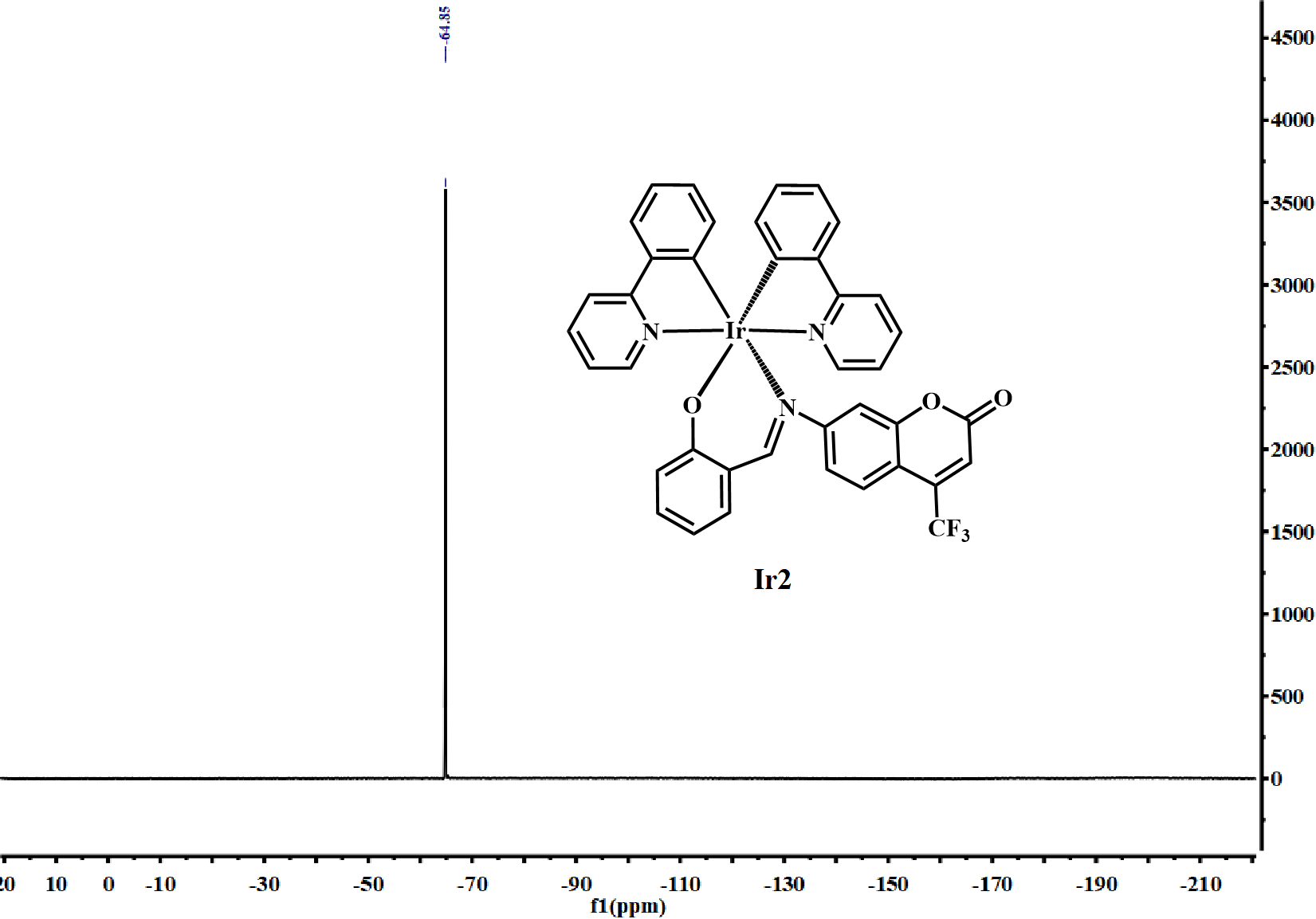

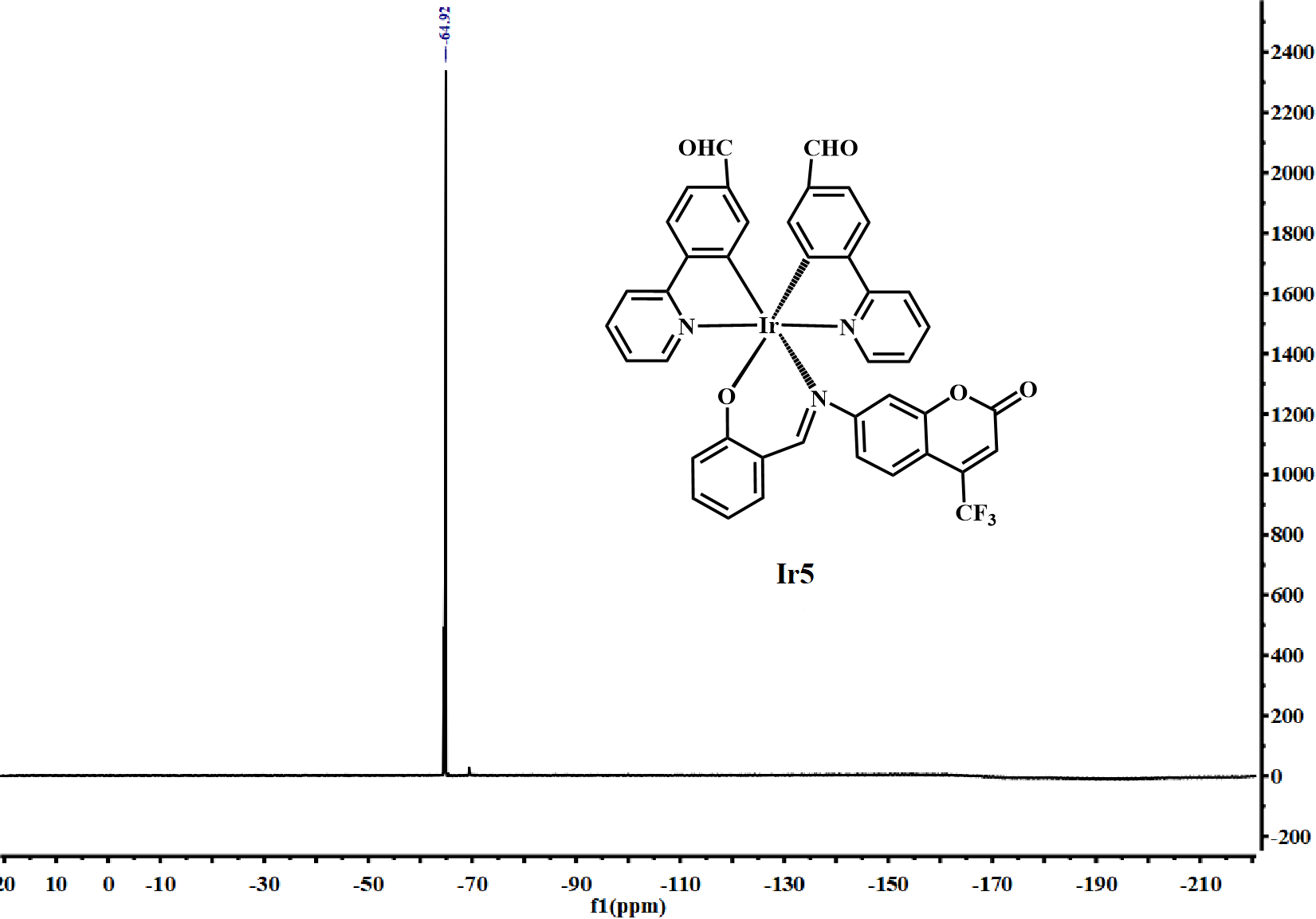
 **Supplementary Figure S4.** ^19^F NMR spectra (471 MHz) of **Ir2 and Ir5**.


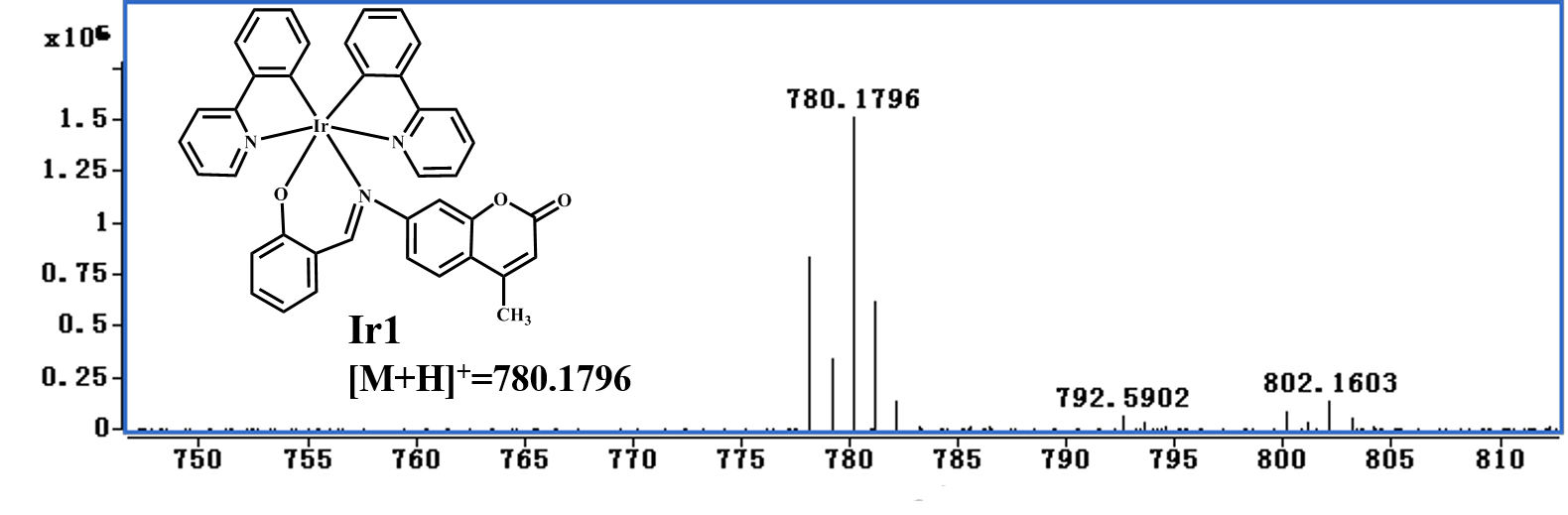

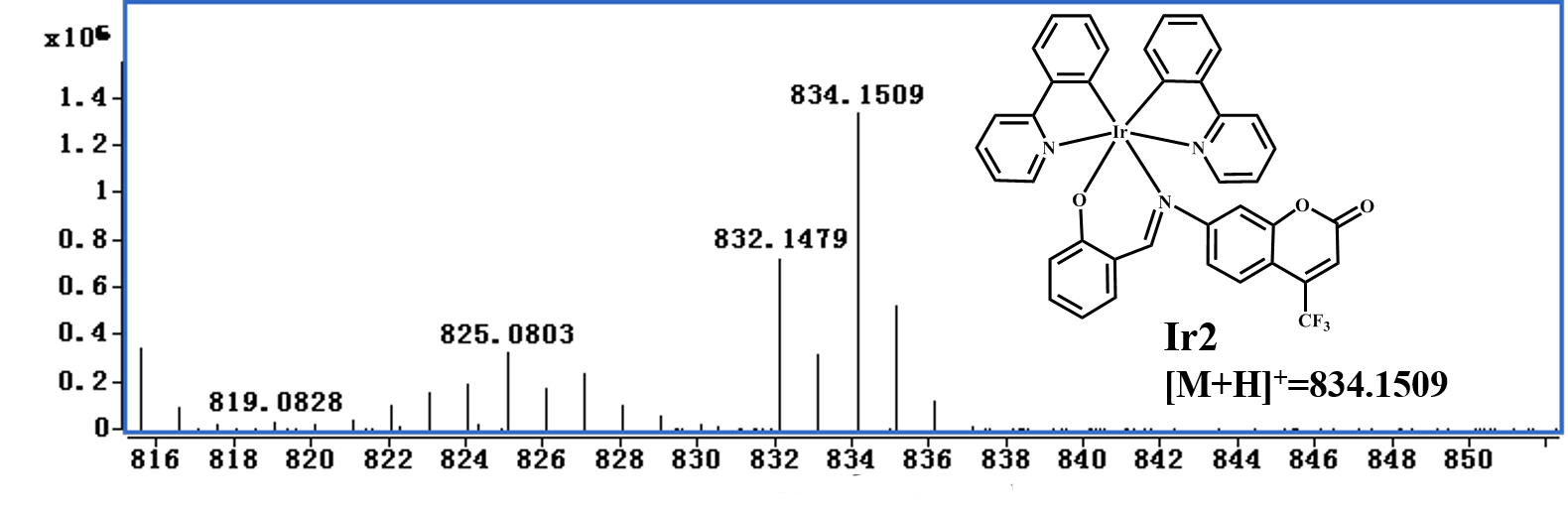

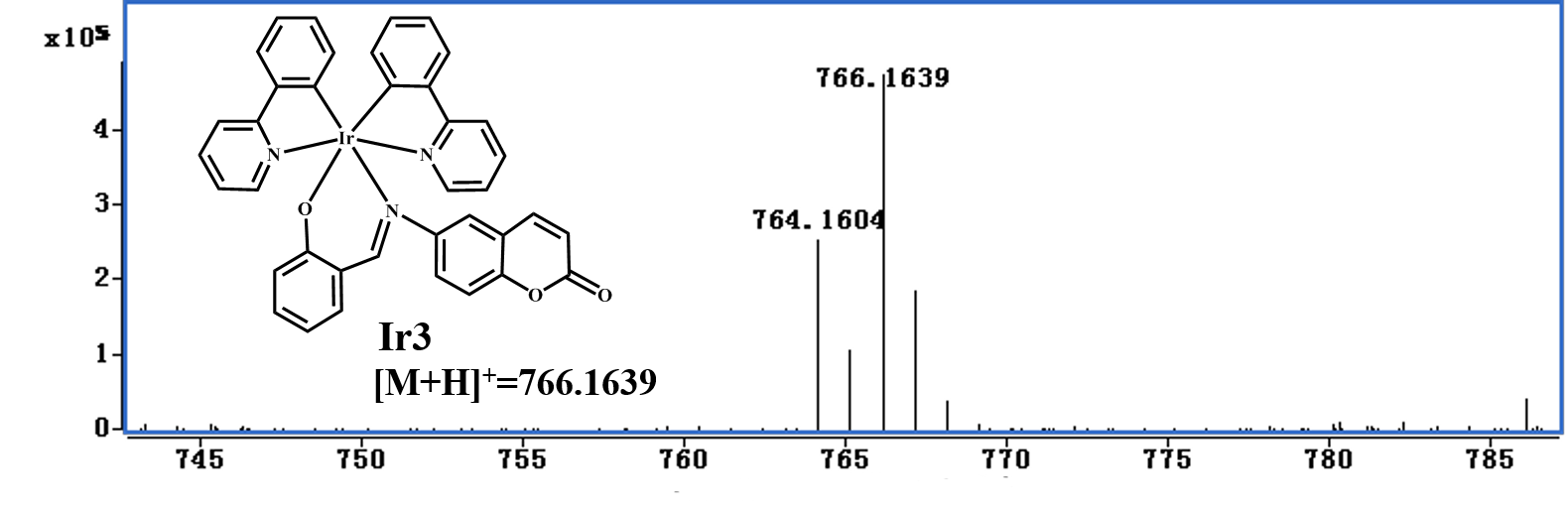


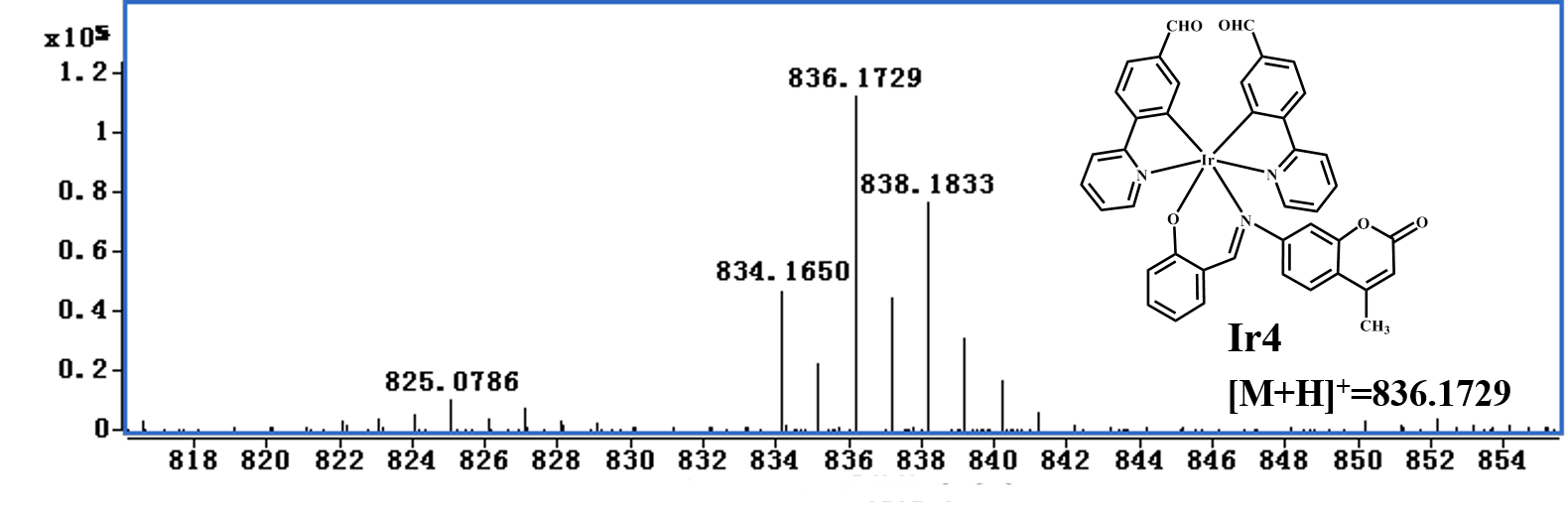


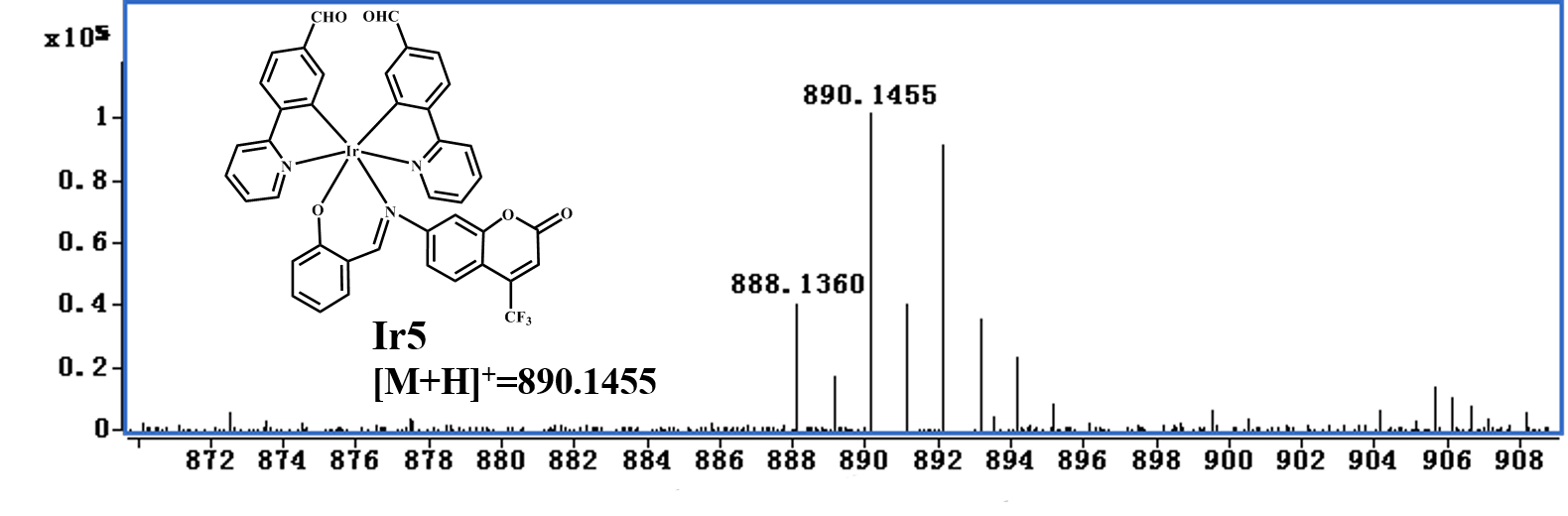


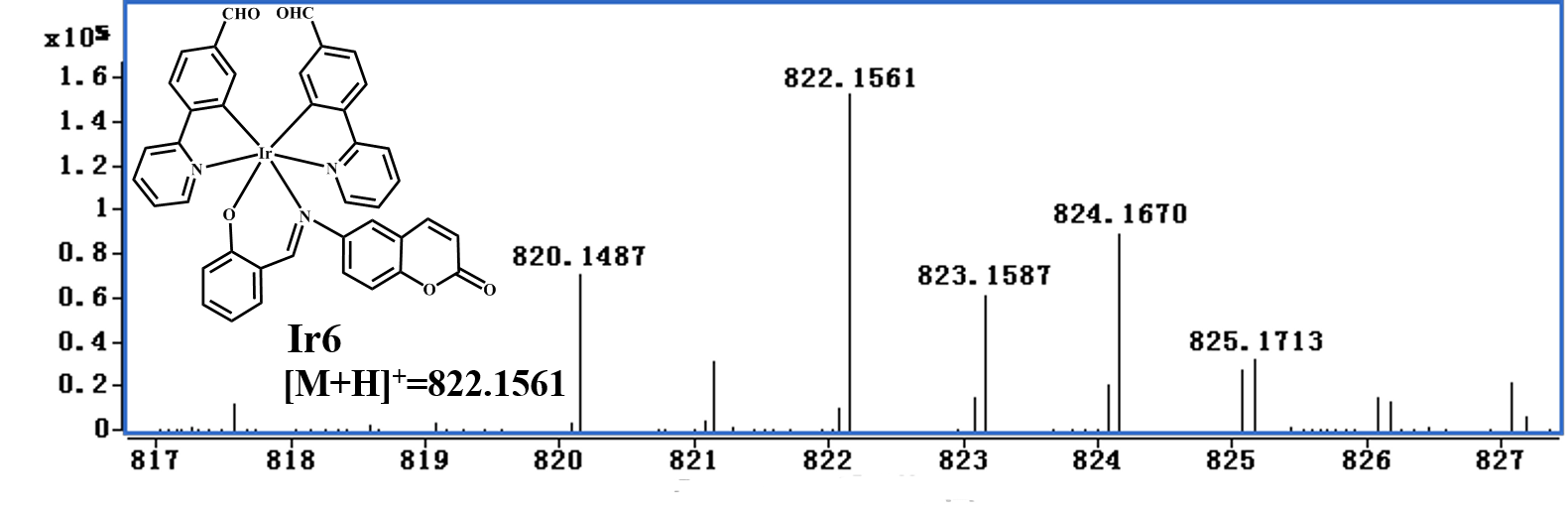


**Supplementary Figure S5.** ESI-MS spectra of **Ir1-Ir6**.

**
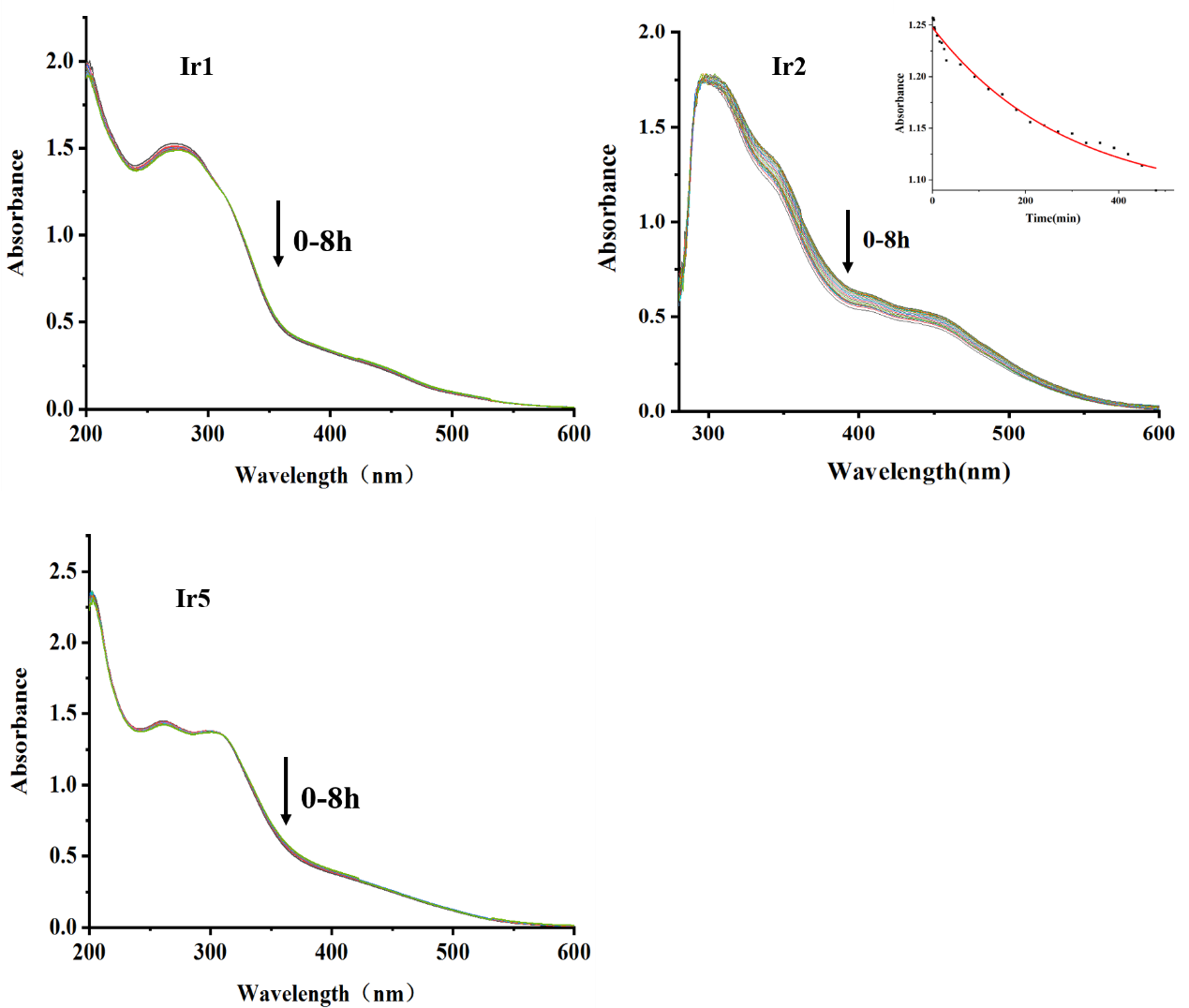
**

**Supplementary Figure S6.** UV-vis spectra of **Ir1**、**Ir2** and **Ir5** in 20% DMSO/80% H_2_O (*v/v*) solution in 8 h at 298 K; Inset: The time-dependent first-order kinetics curve of **Ir2** with water adduct. The arrows show the changes of absorption intensity over 8 h.


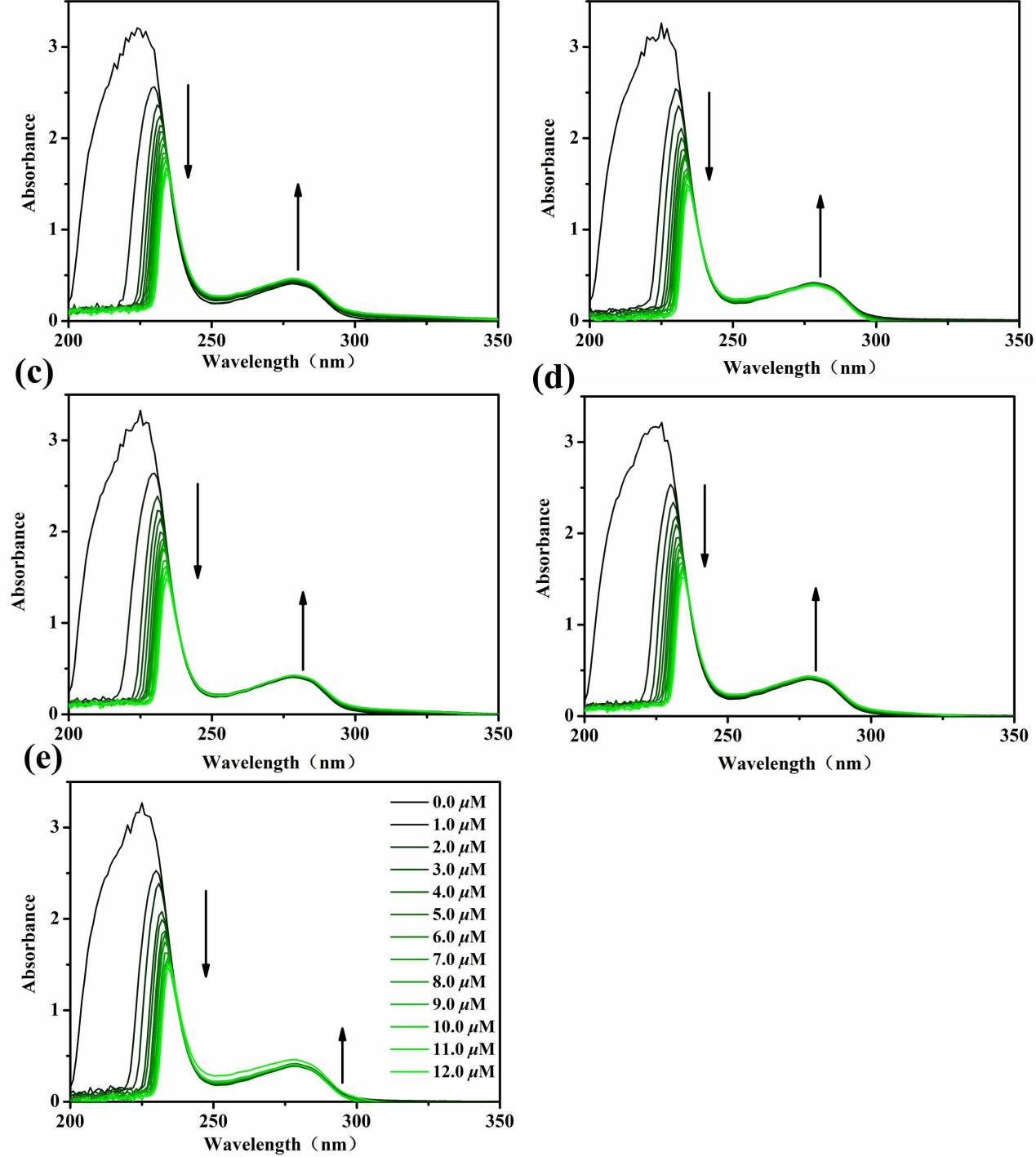


**Supplementary Figure S7.** UV-vis spectra of BSA (10 *μ*M) with **Ir1** (a), **Ir3** (b), **Ir4** (c), **Ir5** (d), **Ir6** (e) (0-12 *μ*M) in Tris-HCl buffer solution at 298 K. The arrows show the changes of absorption intensity with the increase of these complexes.


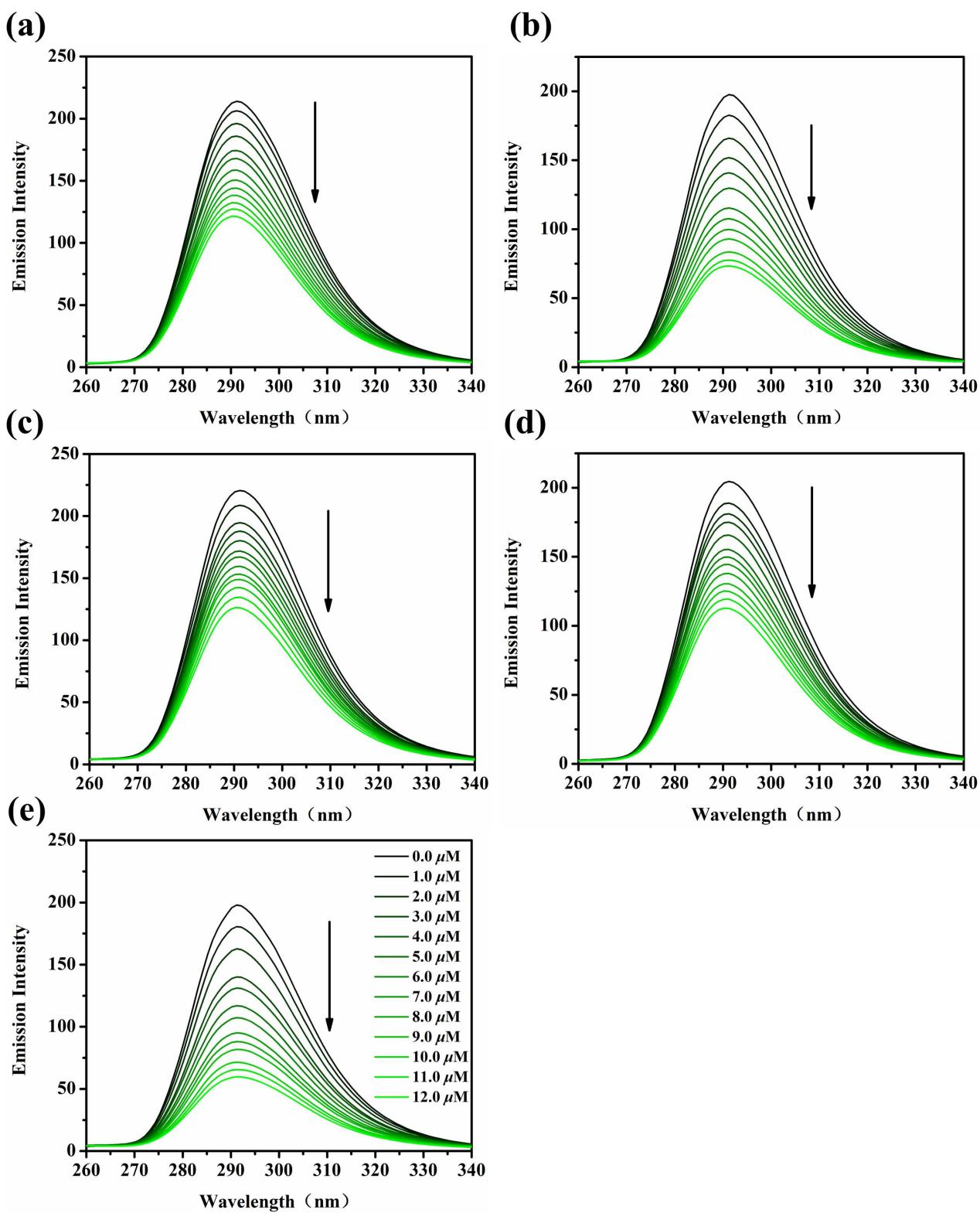


**Supplementary Figure S8.** Synchronous fluorescence spectra of BSA (10 *μ*M) after the addition of **Ir1** (a), **Ir3** (b), **Ir4** (c), **Ir5** (d), **Ir6** (e) (0-12 *μ*M) with a wavelength difference of *Δλ*=15 nm. The arrows showed the changes with the increase of these complexes.


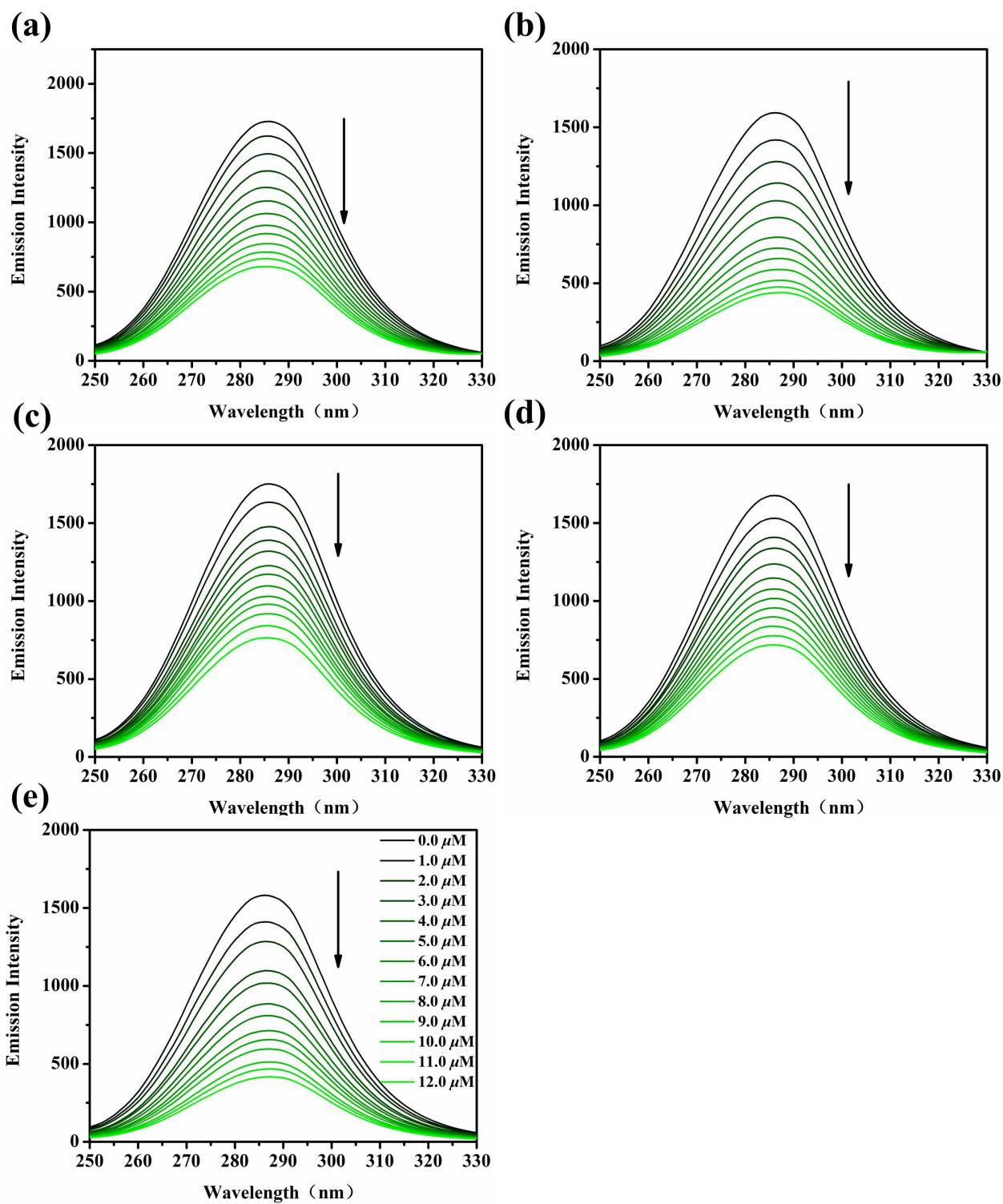


**Supplementary Figure S9.** Synchronous fluorescence spectra of BSA (10 *μ*M) after the addition of **Ir1** (a), **Ir3** (b), **Ir4** (c), **Ir5** (d), **Ir6** (e) (0-12 *μ*M) with a wavelength difference of *Δλ*=60 nm. The arrows showed the changes with the increase of these complexes.


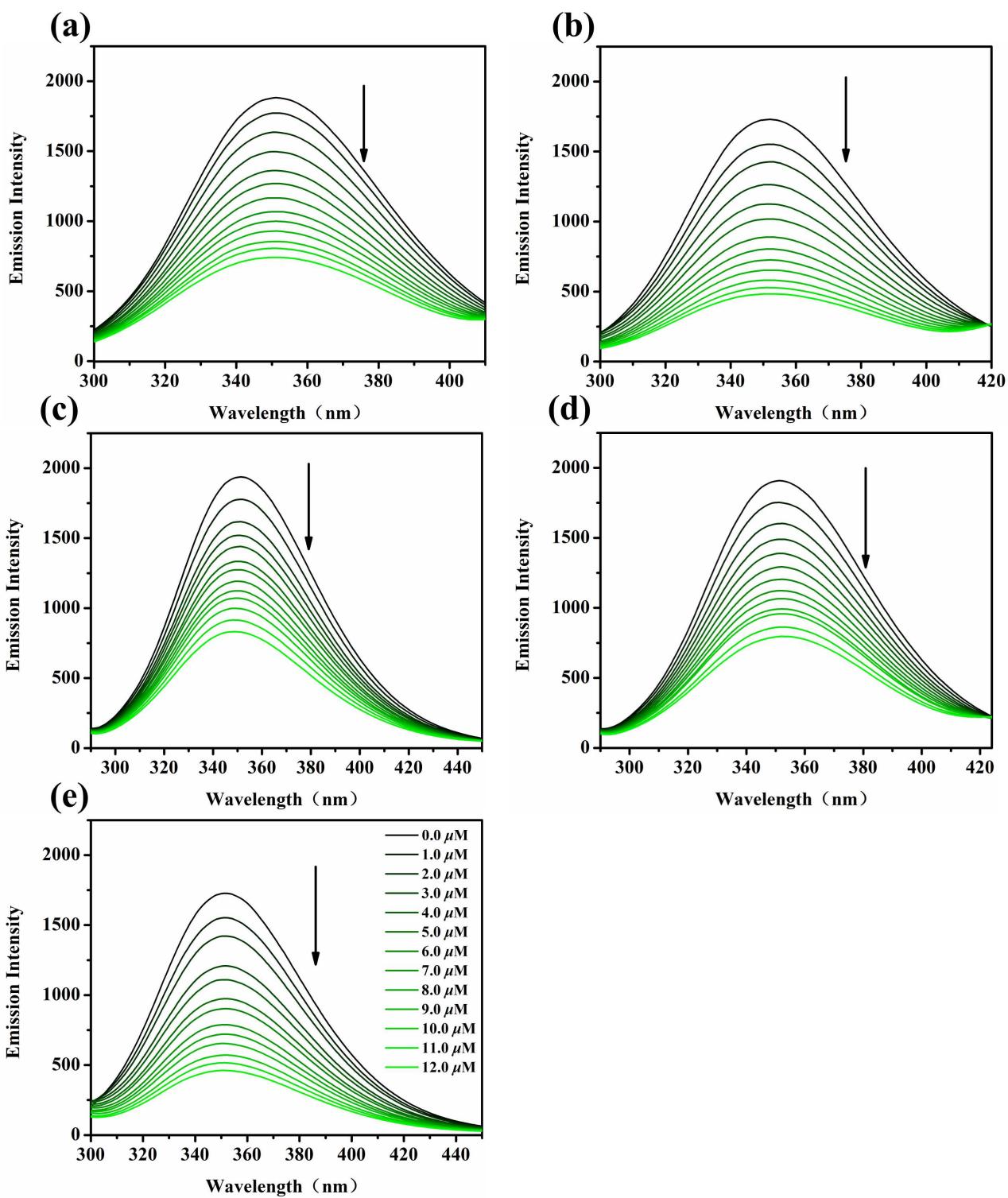


**Supplementary Figure S10.** Fluorescence spectra of BSA (10 *μ*M) with **Ir1** (a), **Ir3** (b), **Ir4** (c), **Ir5** (d), **Ir6** (e) (0-12 *μ*M) in Tris-HCl buffer solution at 298 K. The arrows showed the changes with the increase of these complexes.


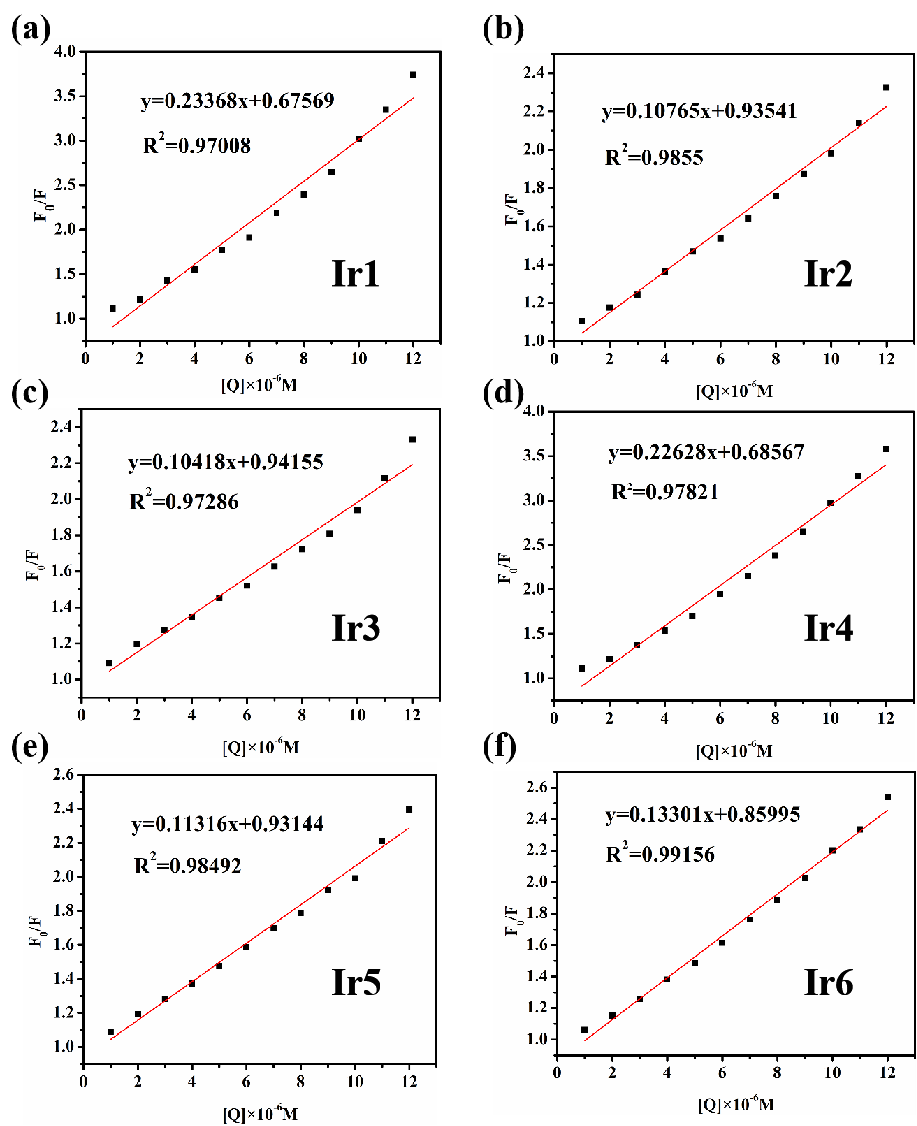


**Supplementary Figure S11.** The relationship between the concentration of **Ir1-Ir6** F_0_/F.


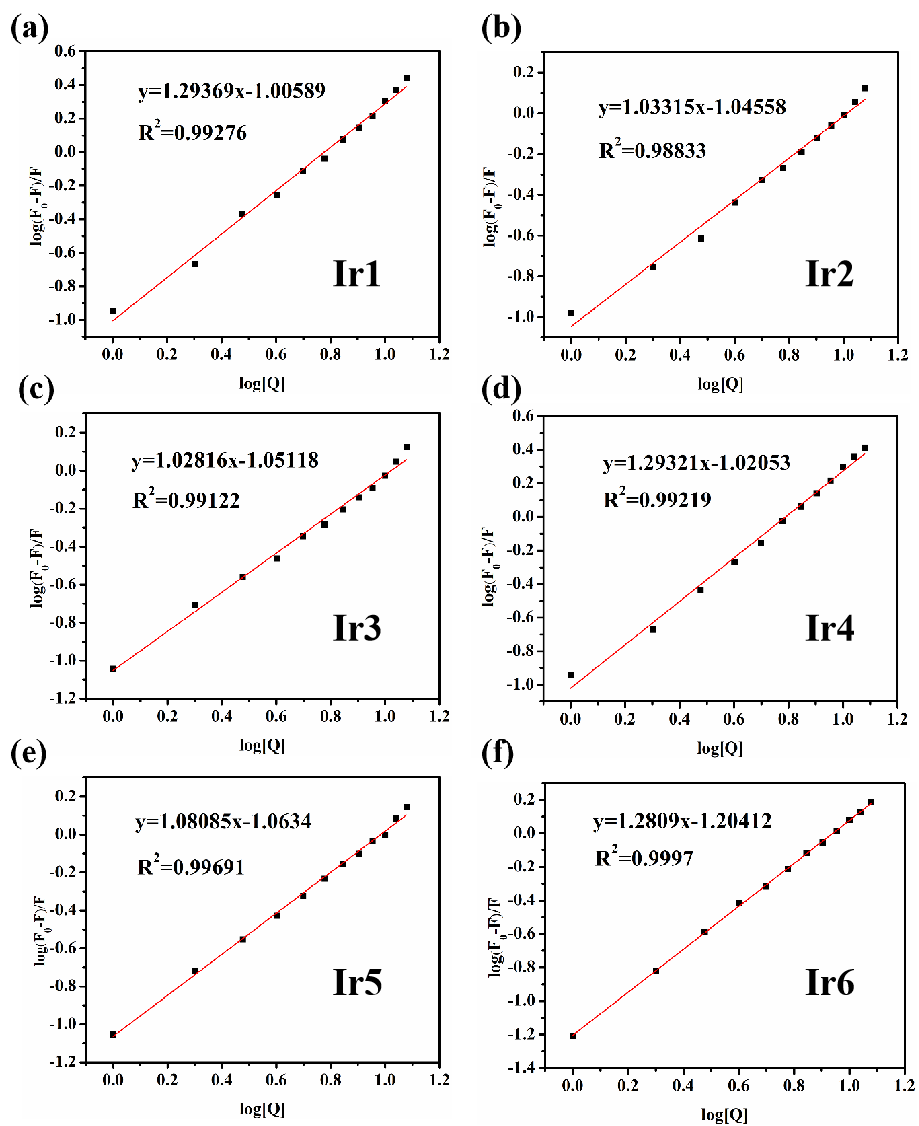


**Supplementary Figure S12.** The graph of **Ir1-Ir6** log[(F_0_-F)/F] and log[Q].


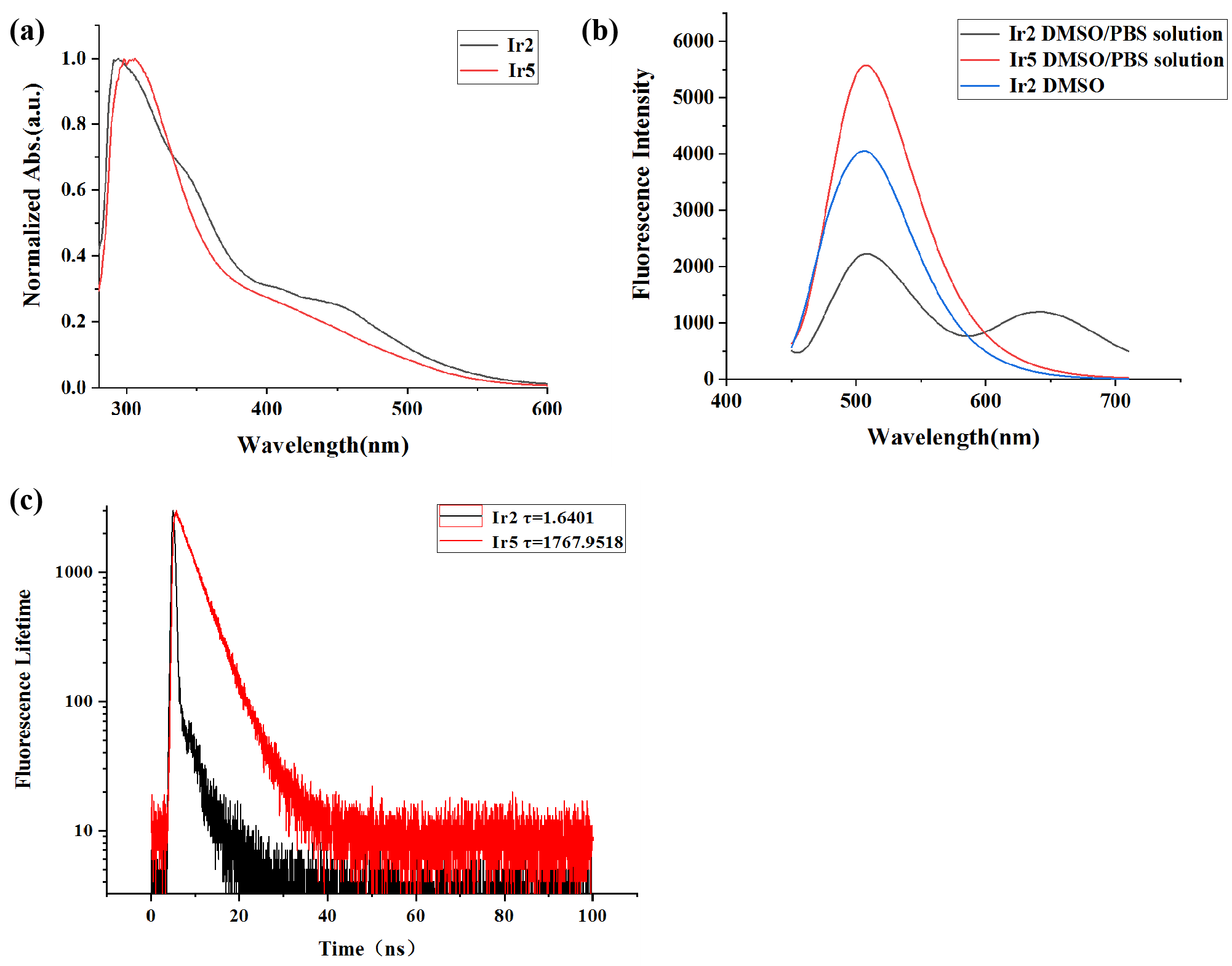


**Supplementary Figure S13.** **(a)** UV-vis spectra of **Ir2** and **Ir5** (20 *μ*M) in 20% DMSO/80% PBS buffer solution (*v/v*). **(b)** Fluorescence spectra of **Ir2** and **Ir5** (20 *μ*M) in DMSO/PBS buffer solution and **Ir2** (20 *μ*M) in pure DMSO. **(c)** Fluorescence lifetime of **Ir2** and **Ir5** (20 *μ*M) in 20% DMSO/80% PBS buffer solution (*v/v*).

**
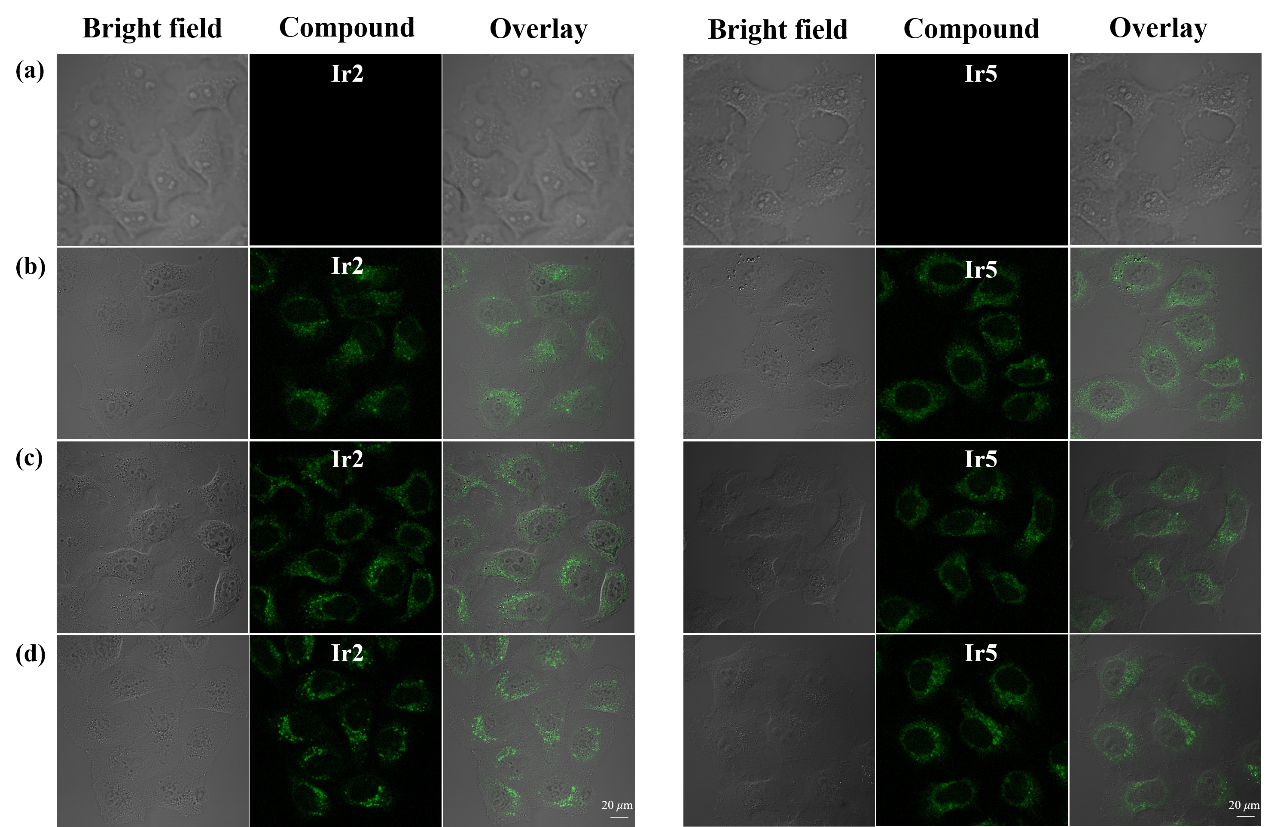
**

**Supplementary Figure S14.** Confocal images of A549 cells after hatched in **Ir2** and **Ir5** (10 *μ*M) under different conditions. The cells were treated with (a) at 277 K for 1 h; (b) at 310 K for 1 h; (c) exposed to CCCP (10 *μ*M) at 310 K for 1 h; (d) exposed to chloroquine (50 *μ*M) at 310 K for 1 h. (*λ_ex_* = 405 nm, *λ_em_* = 430-490 nm). Scale bars: 20 *μ*m.

**
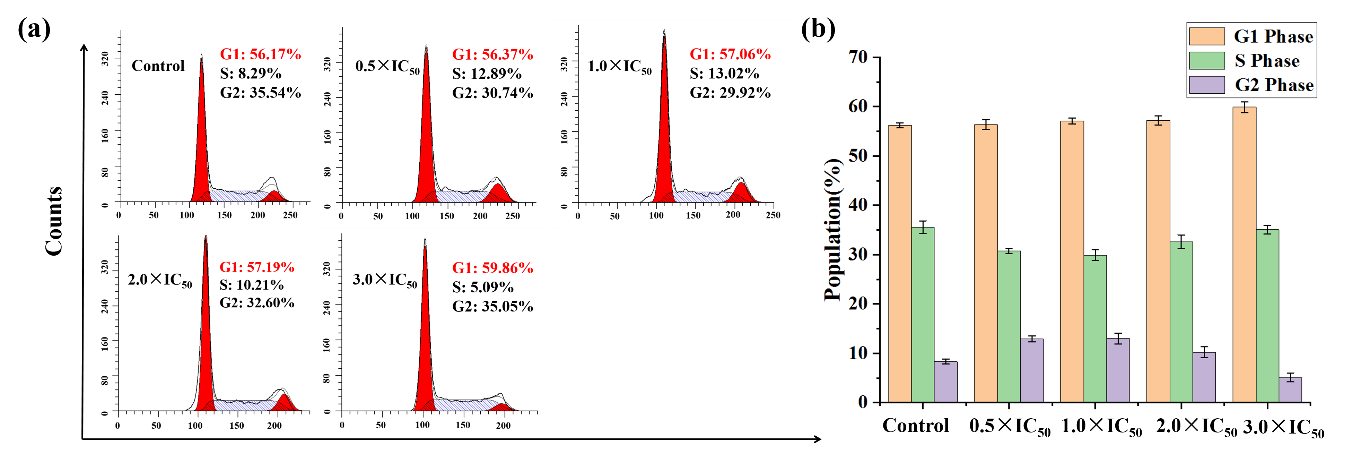
**

**Supplementary Figure S15. (a)** Diagrams of BEAS-2B cells growth cycle distribution stained by PI/RNase after hatched with Ir2 for 24 h. **(b)** Distribution of BEAS-2B cells cycle analysis after hatched with Ir2 for 24 h.


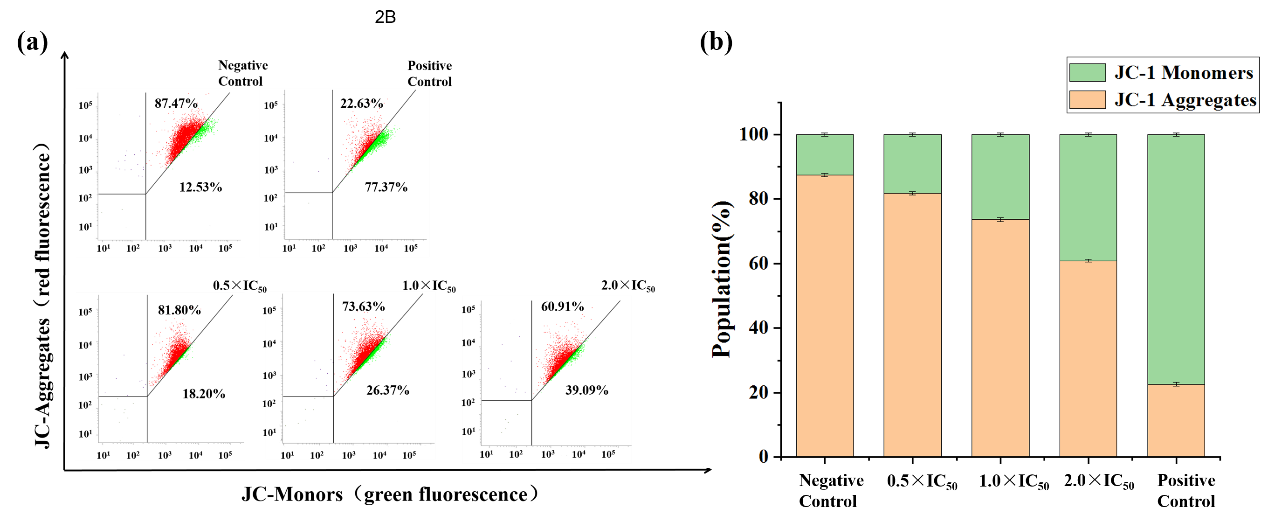


**Supplementary Figure S16. (a)** Changes in MMP of BEAS-2B cells induced by **Ir2**. Data are quoted as mean ± SD of two replicates. **(b)** **Ir2** induced BEAS-2B cell MMP histogram.

**
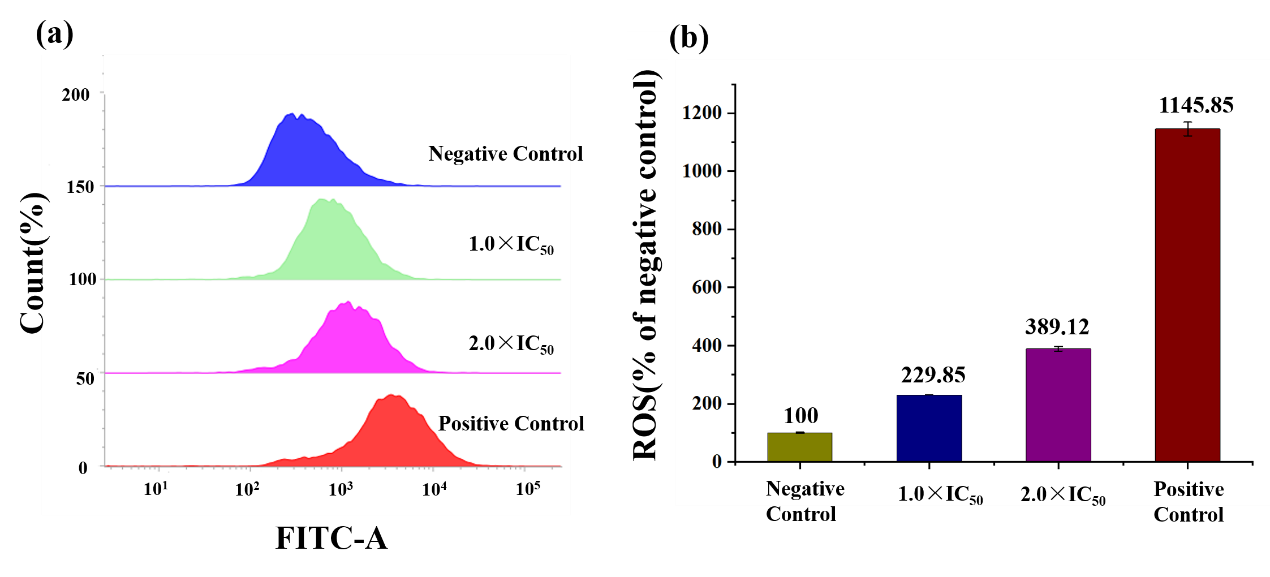
**

**Supplementary Figure S17. (a)** ROS induction in BEAS-2B cells treated with **Ir2** at the 1.0×IC_50_ and 2.0×IC_50_ for 24 h. **(b)** Histograms of ROS levels in BEAS-2B cells.

**
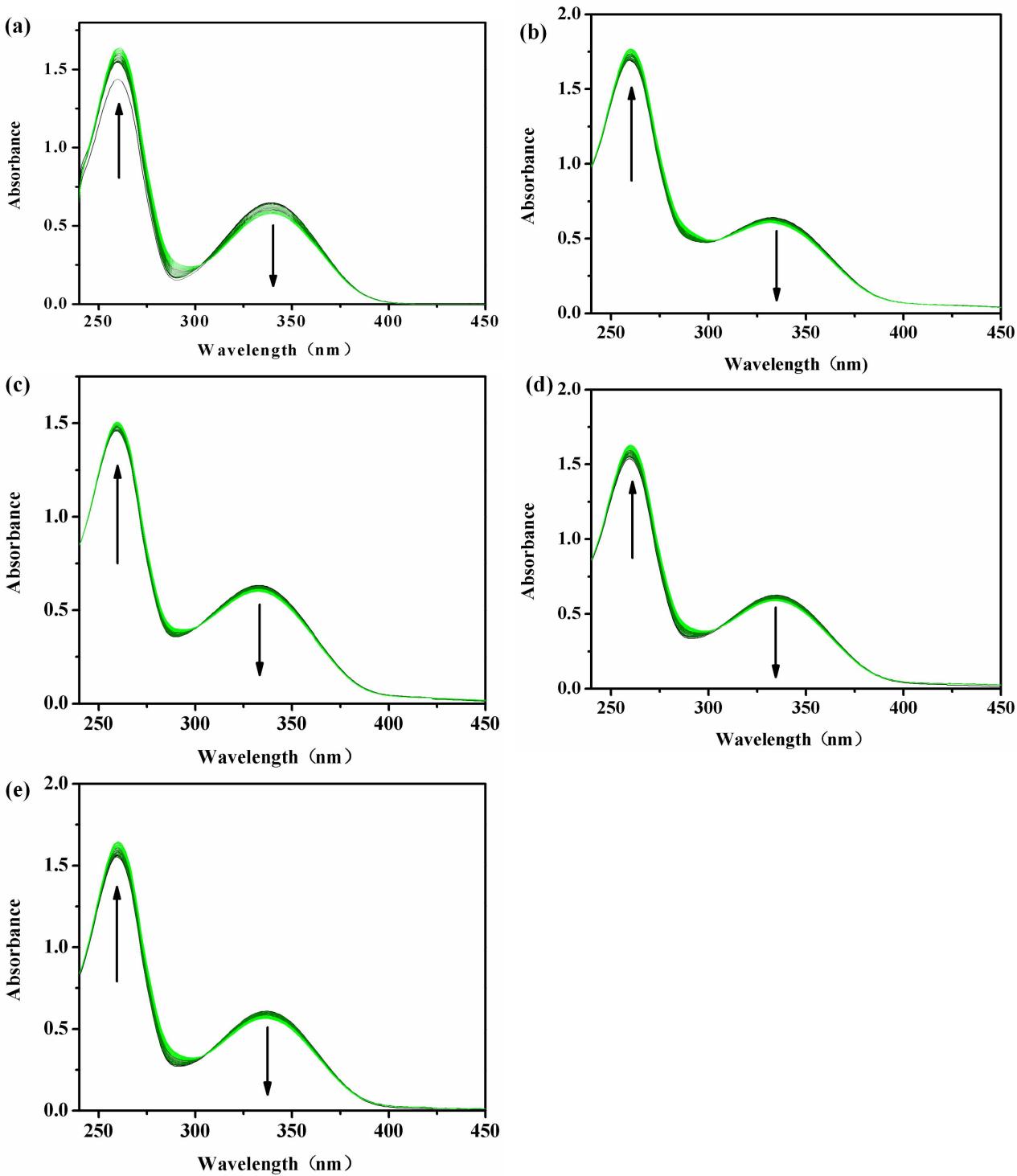
**

**Supplementary** Figure S18. UV-vis spectra of of NADH (100.0 *µ*M) catalyzed by **Ir1** (a), **Ir3** (b), **Ir4** (c), **Ir5** (d), **Ir6** (e) (1.0 *µ*M) in 10% MeOH/90% H_2_O (*v:v*) at 298 K over 8 h.

**Supplementary Table S1**. Crystallographic data for **Ir1**.

| Formula | C_39_H_28_IrN_3_O_3_ | Volume (Å^3^ ) | 3785.0(6) |
| --- | --- | --- | --- |
| MW | 778.87 | *Z* | 4 |
| Crystal size (mm) | 0.43×0.21×0.18 | density (calc) (Mg·m^-3^) | 1.655 |
| *λ*(Å) | 0.71073 | abs coeff (mm^−1^) | 3.853 |
| Temperature (K) | 298 | F(000) | 1872 |
| Crystal system | Monoclinic | *θ* range (deg) | 2.36 to 25.02 |
| Space group | P2(1)/c | index ranges | -10≤h≤10, -21≤k≤15, -28≤l≤25 |
| a (Å) | 8.6897(8) | reflns collected | 18981 |
| b (Å) | 18.3288(17) | indep reflns | 6673 [R(int) = 0.1111] |
| c (Å) | 23.923(2) | data / restraints / params | 6673 / 6 / 470 |
| *α* (°) | 90 | final R indices [I > 2σ(I)] | R1 = 0.0566, wR2 = 0.1462 |
| *β* (°) | 96.598(2) | GOF largest diff peak and hole | 1.031 2.563 and -1.496 |
| *γ* (°) | 90 |  |  |

**Supplementary Table S2**. Selected distances (Å) and angles (°) between atoms for **Ir1**.

| Ir-N_1_ | 2.141(7) |
| --- | --- |
| Ir-N_2_ | 2.045(7) |
| Ir-N_3_ | 2.035(8) |
| Ir-O_1_ | 2.151(6) |
| N_1_-Ir-C_24_ | 173.6(3) |
| N_1_-Ir-C_35_ | 96.2(3) |
| N_1_-Ir-N_3_ | 91.9(3) |
| N_1_-Ir-N_2_ | 95.5(3) |
| O_1_-Ir-C_24_ | 89.4(3) |
| O_1_-Ir-C_35_ | 175.0(3) |
| O_1_-Ir-N_3_ | 95.3(3) |
| O_1_-Ir-N_2_ | 91.4(3) |
| O_1_-Ir-N_1_ | 86.6(3) |

**Supplementary Table S3.** IC_50_ values of reported cyclometallic Ir^III^ complexes against A549 and BEAS-2B cells.

| **Complexs** | **IC_50_ (*****μ*M)** | | **Ref.** |
| --- | --- | --- | --- |
|  | **A549 cell** | **BEAS-2B cell** |  |
| 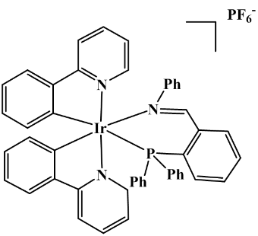 | 3.8 ± 0.2 | / | **(Xu et al., 2020)** |
| 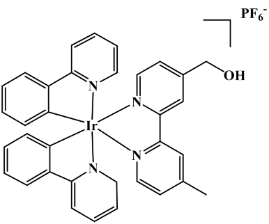 | 13.8±1.1 (48 h) | / | **(Ye et al., 2017)** |
| 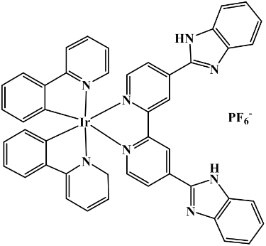 | 0.69±0.1 (48 h) | / | **(Li et al., 2020)** |
| 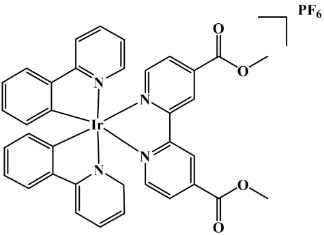 | >100 | / | **(Wang et al., 2016)** |
| 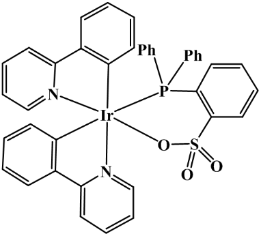 | >100 | / | **(Du et al., 2019)** |
| 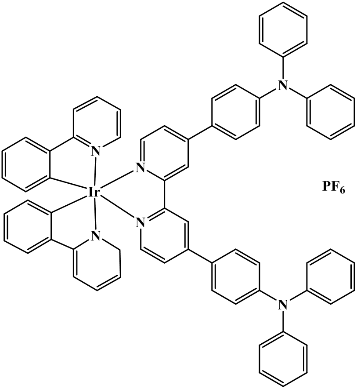 | 80.91±8.65 | >100 | **(Liu et al., 2019)** |
| 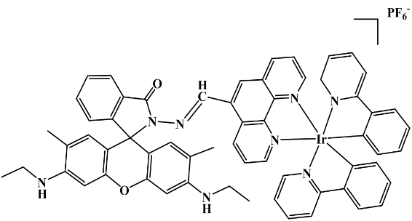 | 4.6±1.1 | 11.7±0.7 | **(Ma et al., 2019)** |

**Supplementary Table S4.** Flow cytometry analysis to determine the percentages of apoptotic cells, using Annexin V-FITC/PI staining, after exposing A549 cells to **Ir2**.

|  | **Concentration** | **Percentage (%)** | | | |
| --- | --- | --- | --- | --- | --- |
|  |  | **Viable** | **Early apoptosis** | **Late apoptosis** | **Non-viable** |
| **Control** |  | 94.10±0.44 | 0.39±0.65 | 4.85±0.49 | 0.18±0.54 |
| **Ir2** | **0.5×IC_50_** | 72.70±0.41 | 2.48±0.51 | 18.80±0.31 | 6.02±0.41 |
|  | **1.0×IC_50_** | 68.30±0.64 | 5.24±0.64 | 21.50±0.33 | 4.96±0.43 |
|  | **2.0×IC_50_** | 14.70±0.72 | 9.81±0.43 | 52.50±0.43 | 22.99±0.52 |

**Supplementary Table S5.** Flow cytometry analysis to determine the percentages of apoptotic cells, using Annexin V-FITC/PI staining, after exposing BEAS-2B cells to **Ir2**.

|  | **Concentration** | **Percentage (%)** | | | |
| --- | --- | --- | --- | --- | --- |
|  |  | **Viable** | **Early apoptosis** | **Late apoptosis** | **Non-viable** |
| **Control** |  | 94.00±1.60 | 2.55±1.62 | 2.34±1.74 | 1.15±0.91 |
| **Ir2** | **0.5×IC_50_** | 88.60±1.80 | 5.78±1.43 | 2.70±1.96 | 2.94±0.63 |
|  | **1.0×IC_50_** | 83.30±1.42 | 9.23±1.92 | 4.00±0.96 | 3.47±0.42 |
|  | **2.0×IC_50_** | 79.20±2.01 | 6.40±0.92 | 6.61±1.55 | 7.08±0.51 |

**Supplementary Table S6.** Cell cycle analysis carried out by flow cytometry using PI staining after exposing A549 cells to complex Ir2.

|  | **Concentration** | **Percentage (%)** | | |
| --- | --- | --- | --- | --- |
|  |  | **G_0_/G_1_ phase** | **S phase** | **G_2_/M phase** |
| **Control** |  | 56.27±0.43 | 24.19±0.44 | 19.54±0.61 |
| **Ir2** | **0.5×IC_50_** | 59.81±0.48 | 28.23±0.42 | 11.97±0.58 |
|  | **1.0×IC_50_** | 61.77±0.46 | 24.57±0.49 | 13.67±0.39 |
|  | **2.0×IC_50_** | 65.96±0.52 | 21.43±0.55 | 12.62±0.48 |
|  | **3.0×IC_50_** | 70.81±0.62 | 18.39±0.48 | 10.80±0.49 |

**Supplementary Table S7.** Cell cycle data of BEAS-2B cells treated with **Ir2** by flow cytometry.

|  | **Concentration** | **Percentage (%)** | | |
| --- | --- | --- | --- | --- |
|  |  | **G_0_/G_1_ phase** | **S phase** | **G_2_/M phase** |
| **Control** |  | 56.17±0.51 | 8.29±1.32 | 35.54±0.52 |
| **Ir2** | **0.5×IC_50_** | 56.37±1.21 | 12.89±0.52 | 30.74±0.63 |
|  | **1.0×IC_50_** | 57.06±0.62 | 13.02±1.13 | 29.92±1.12 |
|  | **2.0×IC_50_** | 57.19±0.93 | 10.21±1.42 | 32.60±1.11 |
|  | **3.0×IC_50_** | 59.86±1.11 | 5.09±0.92 | 35.05±0.92 |

**Supplementary Table S8.** The mitochondrial membrane polarization of A549 cells induced by **Ir2**.

|  | **Concentration** | **Percentage (%)** | |
| --- | --- | --- | --- |
|  |  | **JC-1 Aggregates** | **JC-1 Monomer**s |
| **Negative Control** |  | 87.47±0.54 | 12.53±0.49 |
| **Positive Control** |  | 22.63±0.55 | 77.37±0.55 |
| **Ir2** | **0.5×IC_50_** | 81.80±0.53 | 18.20±0.50 |
|  | **1.0×IC_50_** | 73.63±0.58 | 26.37±0.53 |
|  | **2.0×IC_50_** | 60.91±0.51 | 39.09±0.57 |

**Supplementary Table S9.** The mitochondrial membrane polarization of BEAS-2B cells induced by **Ir2**.

|  | **Concentration** | **Percentage (%)** | |
| --- | --- | --- | --- |
|  |  | **JC-1 Aggregates** | **JC-1 Monomers** |
| **Negative Control** |  | 90.37±0.42 | 9.63±0.45 |
| **Positive Control** |  | 11.60±0.51 | 88.40±0.33 |
| **Ir2** | **0.5×IC_50_** | 86.56±0.44 | 13.44±0.43 |
|  | **1.0×IC_50_** | 71.24±0.43 | 28.76±0.41 |
|  | **2.0×IC_50_** | 50.13±0.47 | 49.87±0.33 |

**Reference**

Du, Q., Zhao, L., Guo, L., Ge, X., Zhang, S., Xu, Z., et al. (2019). Lysosome-targeted Cyclometalated Iridium (III) Anticancer Complexes Bearing Phosphine-Sulfonate Ligands. *Applied Organometallic Chemistry* 33(2)**,** e4746. Doi: <https://doi.org/10.1002/aoc.4746>.

Li, Y., Liu, B., Xu, C.-X., He, L., Wan, Y.-C., Ji, L.-N., et al. (2020). Mitochondria-targeted phosphorescent cyclometalated iridium(III) complexes: synthesis, characterization, and anticancer properties. *JBIC Journal of Biological Inorganic Chemistry* 25(4)**,** 597-607. Doi: https://doi.10.1007/s00775-020-01783-2.

Liu, X., Hao, H., Ge, X., He, X., Liu, Y., Wang, Y., et al. (2019). Triphenylamine-appended cyclometallated iridium(III) complexes: Preparation, photophysical properties and application in biology/luminescence imaging. *Journal of Inorganic Biochemistry* 199**,** 110757. Doi: <https://doi.org/10.1016/j.jinorgbio.2019.110757>.

Ma, W., Ge, X., Guo, L., Zhang, S., Li, J., He, X., et al. (2019). Bichromophoric anticancer drug: Targeting lysosome with rhodamine modified cyclometalated Iridium(III) complexes. *Dyes and Pigments* 162**,** 385-393. Doi: <https://doi.org/10.1016/j.dyepig.2018.10.019>.

Wang, F.-X., Chen, M.-H., Hu, X.-Y., Ye, R.-R., Tan, C.-P., Ji, L.-N., et al. (2016). Ester-Modified Cyclometalated Iridium(III) Complexes as Mitochondria-Targeting Anticancer Agents. *Scientific Reports* 6(1)**,** 38954. Doi: https://doi.10.1038/srep38954.

Xu, Z., Yang, Y., Jia, X., Guo, L., Ge, X., Zhong, G., et al. (2020). Novel cyclometalated iridium(iii) phosphine-imine (P^N) complexes: highly efficient anticancer and anti-lung metastasis agents in vivo. *Inorganic Chemistry Frontiers* 7(5)**,** 1273-1283. Doi: 10.1039/C9QI01492F.

Ye, R.-R., Cao, J.-J., Tan, C.-P., Ji, L.-N., and Mao, Z.-W. (2017). Valproic Acid-Functionalized Cyclometalated Iridium(III) Complexes as Mitochondria-Targeting Anticancer Agents. *Chemistry – A European Journal* 23(60)**,** 15166-15176. Doi: <https://doi.org/10.1002/chem.201703157>.

**
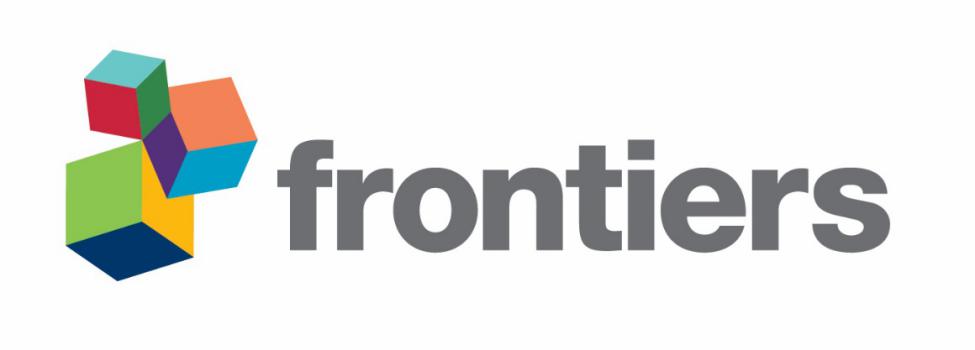
**
