# Supplementary material for: Lysosomal Targeted Cyclometallic Iridium(Ⅲ) Salicylaldehyde-Coumarin Schiff Base Complexes and Anticancer Application
Source: Front Chem. 2022 May 10;10:906954. doi: 10.3389/fchem.2022.906954 (PMC9127163; doi:10.3389/fchem.2022.906954)
Supplement: Supplementary file 2 [file Image1.pdf]

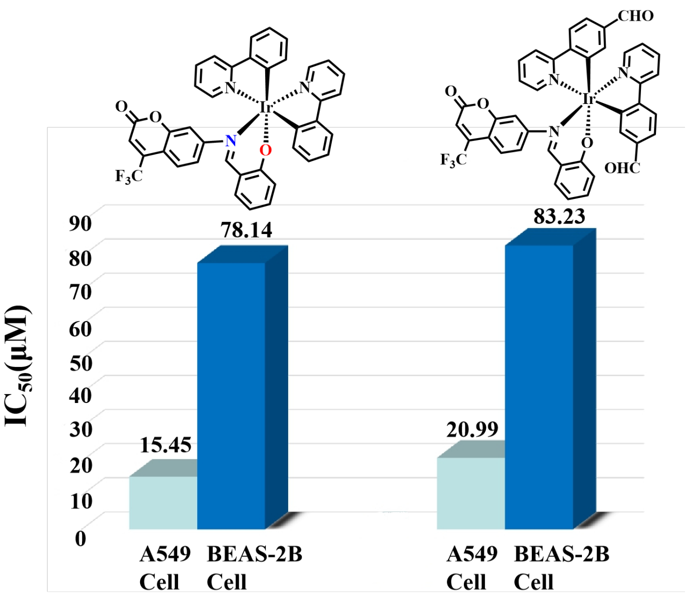


Cyclometallic iridium(III) salicylaldehyde-coumarin Schiff base complexes exhibited favorable cytotoxicity toward A549 cancer cells and BEAS-2B normal cells.
